# Monitoring Reaction Intermediates to Predict Enantioselectivity Using Mass Spectrometry**

**DOI:** 10.1002/anie.202205720

**Published:** 2022-06-03

**Authors:** Roelant Hilgers, Sin Yong Teng, Anamarija Briš, Aleksandr Y. Pereverzev, Paul White, Jeroen J. Jansen, Jana Roithová

**Affiliations:** ^1^ Institute for Molecules and Materials Radboud University Heyendaalseweg 135 6525 AJ Nijmegen The Netherlands; ^2^ Laboratory of Food Chemistry Wageningen University & Research Bornse Weilanden 9 6708 WG Wageningen The Netherlands

**Keywords:** Asymmetric Catalysis, Ion Mobility Spectrometry, Kinetics, Organocatalysis, Reactive Intermediates

## Abstract

Enantioselective reactions are at the core of chemical synthesis. Their development mostly relies on prior knowledge, laborious product analysis and post‐rationalization by theoretical methods. Here, we introduce a simple and fast method to determine enantioselectivities based on mass spectrometry. The method is based on ion mobility separation of diastereomeric intermediates, formed from a chiral catalyst and prochiral reactants, and delayed reactant labeling experiments to link the mass spectra with the reaction kinetics in solution. The data provide rate constants along the reaction paths for the individual diastereomeric intermediates, revealing the origins of enantioselectivity. Using the derived kinetics, the enantioselectivity of the overall reaction can be predicted. Hence, this method can offer a rapid discovery and optimization of enantioselective reactions in the future. We illustrate the method for the addition of cyclopentadiene (CP) to an α,β‐unsaturated aldehyde catalyzed by a diarylprolinol silyl ether.

## Graphical abstract

Diastereomeric intermediates can be separated by ion mobility mass spectrometry. Monitoring of the kinetics of the intermediates allows the prediction of the overall reaction enantioselectivity. The experiments can be performed in a small scale and do not require an isolation of the products.

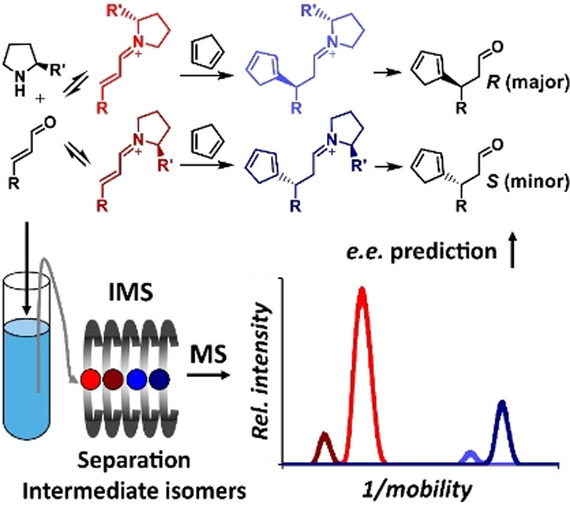

## Introduction

Asymmetric catalysis is of major importance in many fields of chemistry.^[1]^ For decades, enzymes and transition‐metal complexes have been employed as asymmetric catalysts.[^1d^, ^2^] In the past years, organocatalysts such as chiral secondary amines[^1a^, ^3^] have become important, culminating in the Nobel Prize awarded to MacMillan and List in 2021.^[4]^ Traditionally, the development of new enantioselective reactions relies on the optimization of reaction conditions and catalysts, which is typically done by analyzing the yields and enantioselectivities of many parallel reactions. This approach is laborious and requires relatively large amounts of chemicals.[^3a^, ^3b^, ^5^] Here, we propose a quicker, simpler and more economical approach to analyze enantioselectivity of a reaction based on ion mobility spectrometry‐mass spectrometry.

The efficient development of new reactions requires solid mechanistic understanding.^[6]^ In enantioselective reactions it is particularly important to understand how the two (or more) diastereomeric intermediates react, via competing pathways, to one of the two product enantiomers. However, experimental tracking of such competing reaction paths is challenging, because the intermediates are often low in abundance and thus elusive. Real‐time monitoring of reactions by NMR (nuclear magnetic resonance) spectroscopy or other spectroscopic techniques is possible, but signals of reactive intermediates tend to be often very minor compared to the highly abundant reactants and products. Chromatographic approaches are usually not suitable because the reactive intermediates do not ‘survive’ the relatively long chromatographic separations. To obtain mechanistic insights, scientists often study the intermediates by stabilizing them using modified reaction conditions or by tuning the electronic properties of the intermediates. Consequently, the intermediates become stable and their kinetics do not truly correspond to the reactive intermediates or they may even crystallize out of the reaction mixture.^[7]^ Additionally, rationalization by computational chemistry is often used to gain deeper insights into the structure and reactivity of the intermediates. These approaches, however, do not necessarily reveal all reaction details and, even worse, can be driven by confirmation bias. To overcome this, the reaction kinetics of diastereomeric intermediates should be monitored under the operating reaction conditions.^[8]^

**Figure 1:**
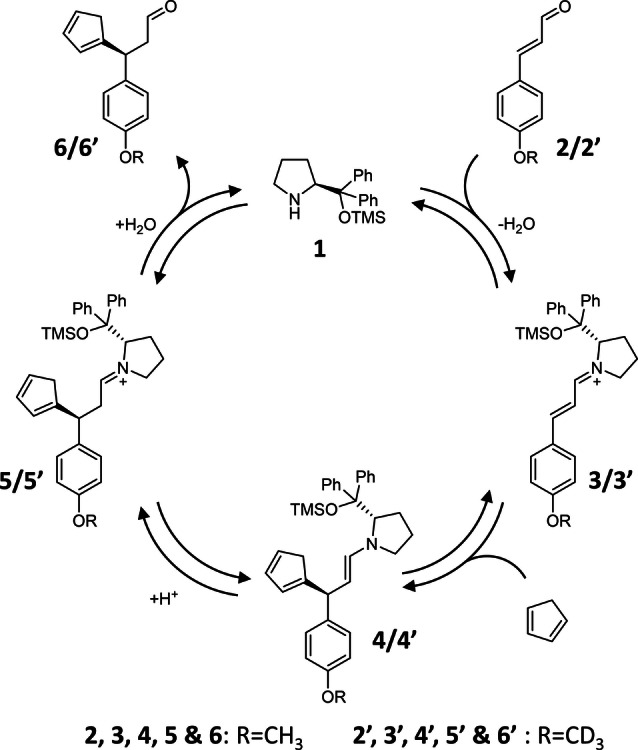
Suggested reaction mechanism for the enantioselective addition of cyclopentadiene (CP) to an α,β‐unsaturated aldehyde, based on Gotoh et al.^[12a]^ Intermediates **3**, **4** and **5** and product **6** are formed as multiple isomers, but we show only the dominant isomeric structure here. In the delayed reactant labelling experiments (see below), we track kinetics of the reaction steps by adding D_3_‐labelled reactant (**2′**) with a reaction time delay of 10 min.

A well‐established technique for the investigation of reaction intermediates is electrospray ionization‐mass spectrometry (ESI‐MS).^[9]^ The advantage of ESI‐MS over other popular analytical techniques consists in a parallel monitoring of analytes according to their specific mass‐to‐charge ratios (*m*/*z*) and with unprecedented sensitivity.^[10]^ The advent of ion mobility (IM)‐mass spectrometry enabled the individual detection of isomers, including diastereomers.^[11]^ Thus, ESI‐IM‐MS opens a way to monitor diastereomeric reaction intermediates in enantioselective reactions. In this paper, we demonstrate the power of the ESI‐IM‐MS approach for tracking diastereomeric reaction pathways of the asymmetric addition of cyclopentadiene (CP) to *p*‐methoxycinnamaldehyde catalyzed by a diaryl prolinol silyl ether (Figure 1). This reaction was selected because organocatalyzed additions to (substituted) cinnamaldehyde are well‐documented in the literature,[^5b^, ^12^] and the intermediates were expected to be easily detectable using ESI‐MS.^[13]^

## Results and Discussion

### Detection and Characterization of Intermediates

**Figure 2:**
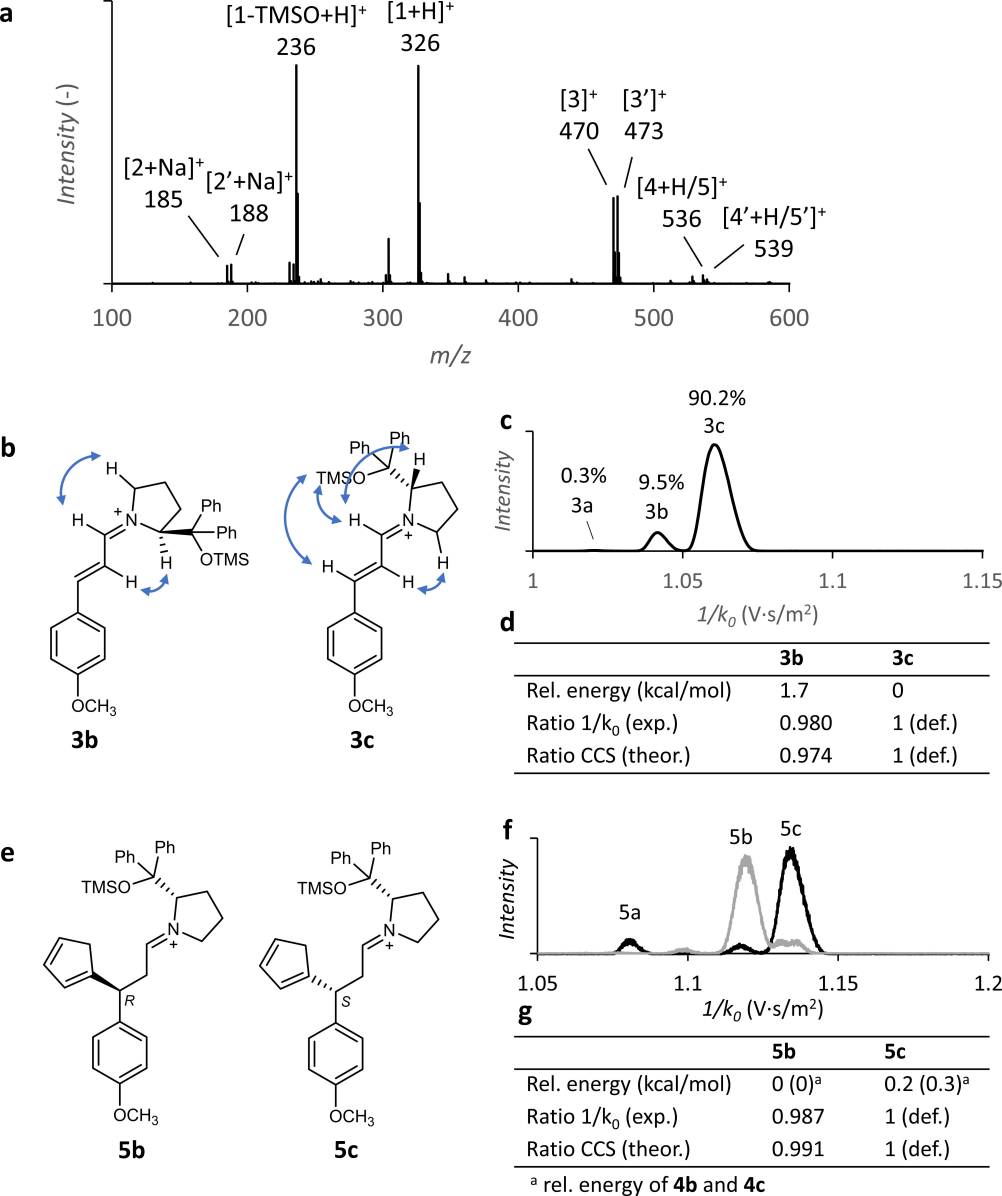
a) ESI‐MS spectrum of the reaction mixture (0.1 mM catalyst **1**, 2 mM reactant **2** and 30 mM cyclopentadiene) after 25 min total reaction time. Isotopically labeled reactant **2′** (2 mM) was added after elapsing of 10 min reaction time. b) Structure annotations of the of iminium isomers **3 b** and **3 c**, with diagnostic NOESY correlations shown as blue arrows. Detailed NMR results (^1^H NMR, TOCSY and NOESY) can be found in the Supporting Information. c) Mobilogram of the ions with *m*/*z* 470, the numbers refer to the peak areas. d) Relative energies and collisional cross sections of **3 b** and **3 c**, e) Structure annotations of ions **5 b** and **5 c**. f) Mobilograms of the ions with *m*/*z* 536 generated obtained from the reaction mixture of **1** and **2** (i.e. forward reaction, in black) and from the mixture of **1** and the product isomers **6** (reverse reaction, in gray). Both mobilograms display the ion mobility distribution after approximately 15 min reaction time. g) Relative energies and collisional cross sections of **5 b** and **5 c**; values in brackets refer to the relative energies of neutral enamines **4 b** and **4 c**.

The ESI‐MS spectrum of the reaction mixture (Figure 2a) shows all expected reaction components: protonated catalyst **1** (*m*/*z* 326), sodiated aldehyde **2** (*m*/*z* 185), the primary iminium intermediate **3** (*m*/*z* 470) as well as the secondary iminium intermediate **5** (*m*/*z* 536). The detected iminium ions **5** may correspond to the iminium ions present in solution, but they can be also formed by protonation of enamines **4** upon ESI. In addition, we have detected ions with *m*/*z* 236, corresponding to the elimination of TMSOH from the protonated catalyst, which presumably occurs during the ionization process (see Supporting Information for explanation).

Separation of the detected ions based on their ion mobilities reveals three isomeric forms of both the primary and secondary iminium intermediates (**3 a**–**3 c** and **5 a**–**5 c** in Figure 2). In the reaction studied here, the formation of the iminium ions **3** has been shown to be the rate‐determining step;^[12a]^ therefore, the **3 a** : **3 b** : **3 c** ratios could directly serve as an initial estimate for the enantioselectivity of the reaction. In order to assess the ionization efficiencies of the isomers of **3 a**–**c**, we obtained a quantitative NMR spectrum of the mixture of the **1** and **2** in the presence of acid. We could identify iminium ions **3 b** and **3 c** and their relative abundance matched the relative abundance of the peak areas of the **3 b** and **3 c** isomers in the mobilogram of ions from the same solution (see Figure S2). This experiment demonstrates that different isomers of the iminium ions have equal ionization efficiencies and thus the isomer ratios can directly be obtained from the mobilogram. Integration of the peaks in the mobilogram resulted in a **3 a** : **3 b** : **3 c** ratio of 0.3 % : 9.5 % : 90.2 % (Figure 2c). A batch control experiment run under the same reaction conditions was performed to quantify the enantiomeric outcome of the addition reaction in order to compare it to the ratio of intermediates **3 a**–**c** detected by ESI‐IM‐MS. The reaction resulted in 88.5 % of the major product enantiomer (*R*) and 11.5 % of the minor product enantiomer (*S*), which is indeed in a good agreement with the ratio of the two main isomers **3 c** and **3 b**. This indicated that the detected intermediate ratios directly determine the stereoselectivity of the reaction (see Supporting Information for the details). However, to make the prediction quantitative and applicable to other types of reactions,^[14]^ we must know the kinetics along the entire reaction pathways. In this case, we must determine which isomer of **3** reacts to form which isomer of **4** and **5** and what are the kinetics along these competing reaction paths. To this end, we first characterized the individual intermediate isomers in more detail.

### Characterization of the Isomers of Intermediate 3

The condensation of secondary amine catalysts with α,β‐unsaturated aldehydes has been reported to result in iminium isomers with *trans* and *cis* C=N bonds, the former typically being the major isomer.[^7a^, ^7b^] To verify that the two most abundant isomers of **3** (i.e. **3 c** and **3 b**) indeed correspond to the *trans* and the *cis* isomers, we performed NMR experiments with an equimolar mixture of catalyst **1** and compound **2** (omitting CP). From the spectra, two iminium isomers could be distinguished with a ratio (≈0.05 : 1) similar to that of **3 b** and **3 c** in the mobilograms (see Figure S2). Based on the unique NOESY (Nuclear Overhauser Effect Spectroscopy) correlations of these isomers, we confirmed that **3 b** and **3 c** correspond to the *cis* and the *trans* isomers, respectively, of the iminium ion **3** (Figure 2b). We further confirmed the identity of **3 b** and **3 c** by finding a good agreement between the ratio of their theoretical CCSs (Collision Cross Sections) and the ratio of their inverse ion mobilities (1/*k*_0_), as CCS and 1/*k*_0_ are linearly correlated (Figure 2d).^[15]^ Despite multiple NMR and IRPD (Infrared Photodissociation) spectroscopy experiments^[16]^ (see Supporting Information), the exact structure of the low‐abundant isomer **3 a** could not be elucidated. Nevertheless, its identity is of low importance for the current study because the expected amount of product formed via **3 a** is negligible (see below).

### Characterization of the Isomers of Intermediates 4 and 5

The ions of *m*/*z* 536 could correspond to protonated enamines **4** and/or iminium ions **5**. Protonation of the enamines during ESI could occur at the nitrogen atom or at the β‐carbon atom. However, the IR photodissociation spectrum of the ions with *m*/*z* 536 does not show any N−H stretching vibration (no band above 3100 cm^−1^; see Figure S3) suggesting that N‐protonated enamines do not form. This is further supported by DFT (Density Functional Theory) calculations which indicate that C‐protonation is favored by 10–12 kcal mol^−1^ (Table S2). Hence, intermediates **4** and **5** are collectively detected as iminium ions **5** in ESI‐MS experiments.

To find out which isomer of the iminium ions **5** reacts to the major product, we have isolated the isomeric mixture of product **6** after the completion of the reaction and mixed it with catalyst **1** to re‐form the iminium ions **5**. Since product **6** is a mixture of *R* (major) and *S* (minor) enantiomers, the reverse reaction is expected to produce at least two isomers of **5**: a major isomer with the cyclopentadienyl group linked in the *R*‐configuration, and a minor isomer with the cyclopentadienyl group linked in the *S*‐configuration. The ESI‐IM‐MS analysis clearly shows that iminium **5 b** is the most abundant isomer formed in the reverse reaction, and that **5 c** is formed in a lower amount, suggesting that **5 b** and **5 c** report on the *R* and *S*‐isomers of intermediates **4**/**5**, respectively (Figure 2f in gray). Further evidence for this assignment was provided by the excellent agreement between the ratio of the theoretical CCSs and experimental inverse mobilities of **5 b** and **5 c** (Figure 2g). The isomer **5 a** was not detected in the reverse reaction. We hypothesize that **4 a**/**5 a** are formed from **3 a**, and correspond to a side product that was removed during the purification of product **6**. The rate of the reverse conjugate addition is negligible because we detected only traces of iminium intermediates **3** in the reverse reaction after long reaction times.

**Figure 3:**
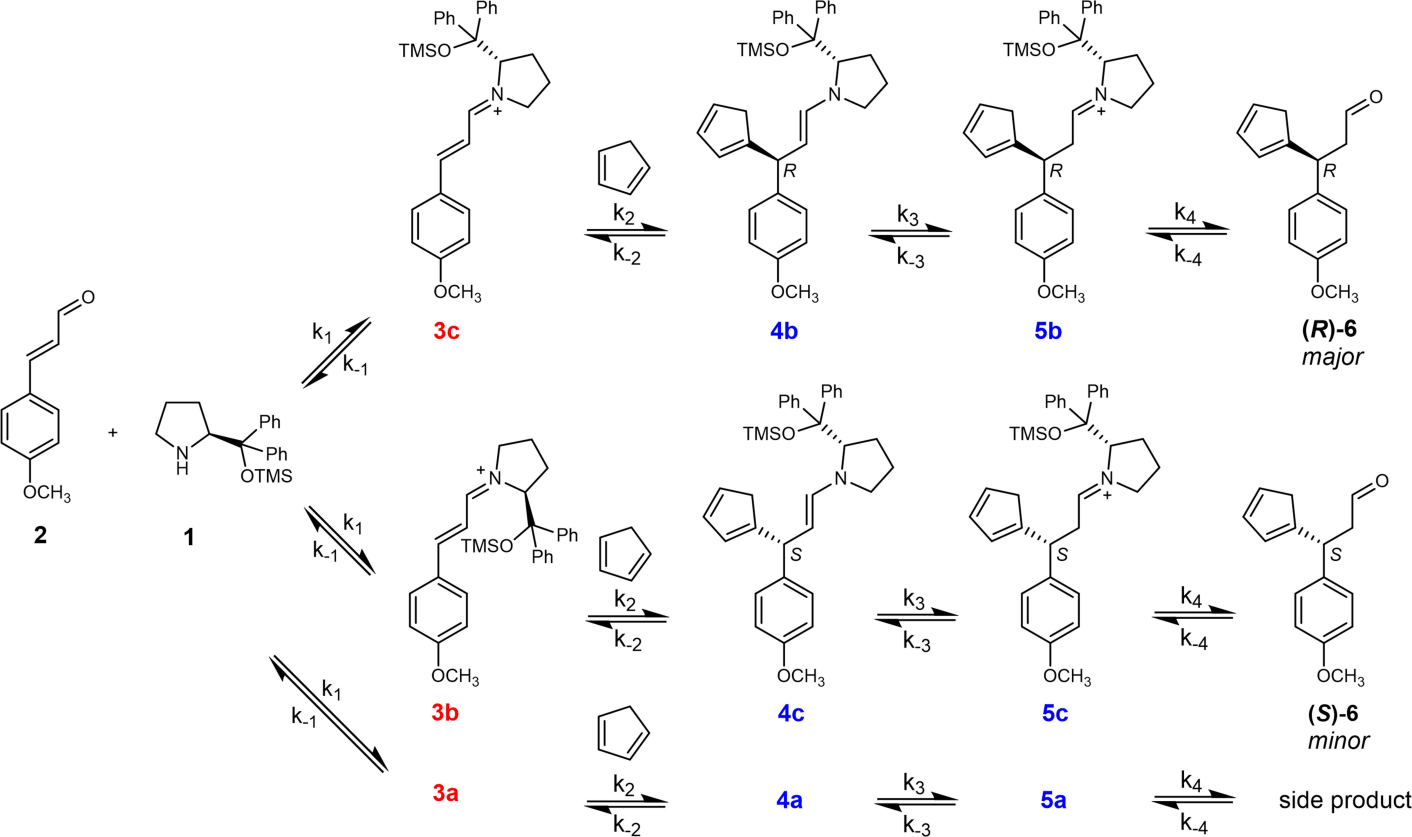
Proposed reaction pathways based on NMR, IRPD and ESI‐IM‐MS experiments described in the main text. The exact structures of **3 a** and **4 a**/**5 a** are unknown and are therefore not shown.

The above‐described assignments allow us to identify the reaction pathways of the individual isomeric intermediates (Figure 3). The reactant and catalyst condense to mainly form iminium ions **3 c** and **3 b**, which have C=N bonds in the *trans*‐ and *cis‐*configurations, respectively. The bulky catalyst side chain forces the CP to attack from the least sterically hindered side of the iminium intermediate.[^7a^, ^17^] Accordingly, **3 c** reacts via **4 b** and **5 b** to yield the *R*‐enantiomer of product **6**, and **3 b** reacts via **4 c** and **5 c** to yield the *S*‐enantiomer of **6**. Isomer **3 a** probably reacts via **4 a**/**5 a** to a side product.

### Determination of the Rate Constants for the Individual Isomeric Intermediates

Although the ratio of the primary intermediate abundances (**3 c** : **3 b**) gives a good estimate for the enantioselectivity in this particular reaction, it cannot be taken as a general rule. The enantioselectivity can be affected by the equilibria involving all the other intermediates along the reaction pathways.^[18]^ Hence, for a complete and correct evaluation we need to know the rate constants indicated in Figure 3 for all competing pathways. To determine these rate constants, we need to monitor the concentration changes of the intermediates in time. In general, the ion intensities in ESI‐MS spectra do not necessarily correlate with the concentrations of the analytes in solution.^[19]^ However, we have developed a method to overcome this problem by using Delayed Reactant Labeling (DRL).^[20]^

**Figure 4:**
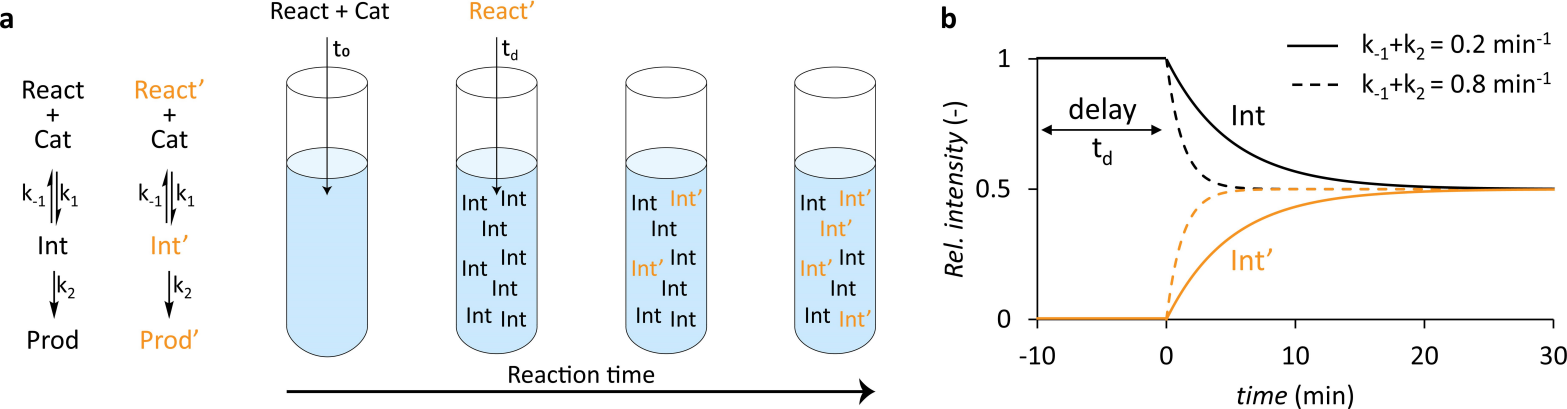
A schematic example of a delayed reactant labeling (DRL) experiment. In (a) the equations are shown for a simple two‐step reaction of the unlabeled (black) and isotopically labeled (yellow) reactant along with a schematic representation of the ratio of labeled/unlabeled intermediates in solution at various stages of the DRL experiment. In (b) a graph is displayed showing theoretical outcomes of a DRL experiment with a time delay of 10 min. Increased values of *k*_−1_ and *k*_2_ result in a shorter lifetime of the intermediate, and thereby in the increased slopes in the DRL curves. For intermediates displaying steady‐state kinetics, depletion rate constants (*k*_−1_+*k*_2_) can be derived using Equation (1).

The key point of DRL is that one of the reactants is added as a mixture of unlabeled and isotopically labeled molecules and that one of them is added with a certain time delay. The ratio between labeled and unlabeled intermediates is then monitored over time. For intermediates displaying steady‐state kinetics, this ratio reflects the depletion rate of these intermediates.^[20]^ The principle behind DRL and a theoretical outcome of such an experiment are illustrated in Figure 4. For a simple 2‐step reaction, in which the intermediate displays steady‐state kinetics, the sum of *k*_−1_ and *k*_2_ can be obtained by fitting the data using Equation 1:

(1) $$\left[\mathrm{Int}{}^{'}\right]{}_{t}=\left[\mathrm{Int}{}^{'}\right]{}_{\mathrm{eq}}\;\left(1-e{}^{-(k{}_{-1}+k{}_{2})\;t}\right)$$

in which [Int′]_t_ and [Int′]_eq_ are the relative concentrations of the labeled intermediate at time t and after equilibrium formation.

**Figure 5:**
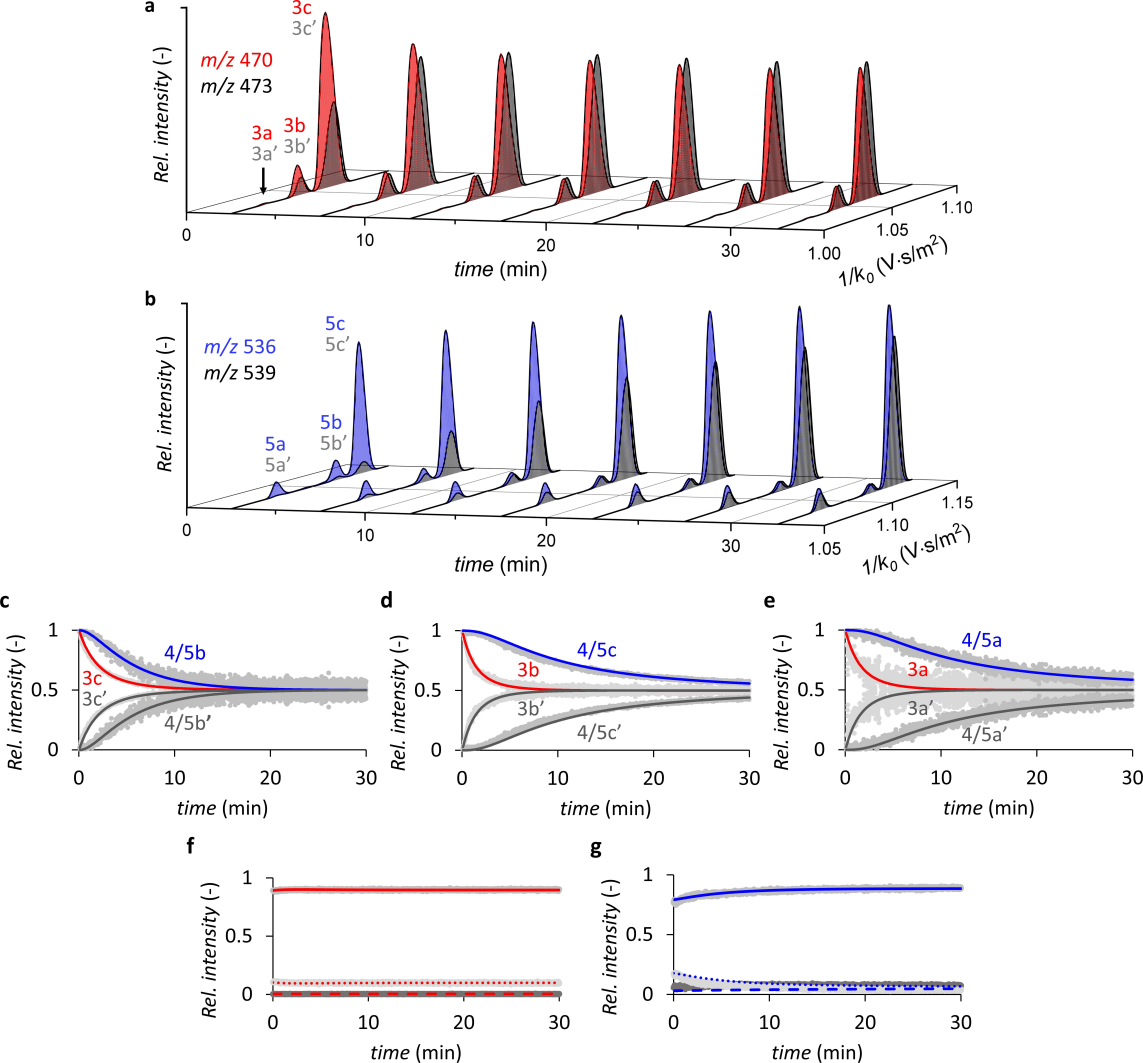
a), b) TIC‐normalized extracted ion mobilograms illustrating the time evolution of individual intermediate isomers for **3** & **3′** (a), and **5** & **5′** (b). c)–e) Time evolution of relative ion intensities of labeled vs. unlabeled intermediates in the delayed reactant labeling experiment for the three pathways shown in Figure 3. Experimental relative ion intensities are shown in light gray. Relative intensities obtained by modelling (see Table 1 for rate constants) are shown for ions **3** (red lines) and **3′** (dark gray) and **5** (blue) & **5′** (dark gray). f), g) Time evolution of the relative ion intensities of the three isomers of intermediate **3** (f) and intermediate **5** (g). Both graphs display the experimental relative ion intensity of isomers a (dark grey), isomers b (light gray) and isomers c (gray), as well as the relative ion intensity obtained by modelling: isomer a (dashed line), isomer b (dotted line) and isomer c (solid line). The blue curves were constructed by summing the predicted concentrations of enamine **4** and iminium ion **5**, using a correction factor of 0.025 (a fitting parameter) to account for the lower ionization efficiency of the enamine.

To monitor the kinetics of the reaction studied here, we have added D_3_‐labeled *p*‐methoxy‐cinnamaldehyde (**2′**) to the reaction mixture with a delay of 10 min. The relative abundances of the isotopically labeled intermediates (**3′** and **5′**) gradually increased over time for all isomers (Figures 5a and b), confirming that they are formed in solution (i.e. not during ESI).^[21]^ All isomers of **3** display steady‐state kinetics, as demonstrated by i) the rapid establishment of a 1 : 1 equilibrium of **3** and **3′** (corresponding to the 1 : 1 concentrations of **2** and **2′**) after addition of **2′** (Figures 5c–e) and ii) the fact that the TIC‐normalized intensities of **3** and **3′** (TIC=total ion current) remain essentially constant after reaching the equilibrium conditions (Figure 5a). Thus, we derived the degradation rate of all isomers of **3** (*k*_−1_+*k*_2_) by fitting the DRL curves using Equation (1). To determine the individual contributions of *k*_−1_ and *k*_2_, the experiment was repeated in absence of CP, so that *k*_2_ equals 0. In this experiment, the iminium ions **3** did not reach steady‐state concentrations within the experimental time; using the Euler method, *k*_−1_ was estimated to be approximately 0 (Figures S5&S6). This indicates that, under the experimental conditions used, the hydrolysis rates (*k*_−1_) of **3 a**–**c** are negligible compared to their reaction rates with CP (*k*_2_) (Table 1).

**Table 1:** Left panel: Rate constants for individual isomeric intermediates obtained by simultaneously fitting the relative intensities of labeled and unlabeled intermediates (Figures 5c–e) and relative intensities of intermediate isomers (Figures 5f and g).^[a]^ Right panel: Comparison between the enantiomer ratio of product **6** predicted by the DRL experiment and experimentally determined by chiral HPLC.

Pathway	*k*_1_ [M^−1^ min^−1^]	*k*_−1_ [min^−1^]	*k*_2_ [min^−1^]	*k*_−2_ [min^−1^]	*k*_3_ [min^−1^]	*k*_−3_ [min^−1^]	*k*_4_ [min^−1^]	Predicted [%] of product **6** ^[b,c]^	Experimental [%] of product **6** ^[d]^
**3 c→4/5 b**	1.50	0.0	0.43	0.025	0.23	0.0	8.0	86.7±1.3 (*R*)	88.5±0.2 (*R*)
**3 b→4/5 c**	0.25	0.0	0.64	0.035	0.35	0.0	0.05	13.3±1.3 (*S*)	11.5±0.2 (*S*)
**3 a→4/5 a**	0.01	0.0	0.57	0.030	0.30	0.0	0.03	–	–

[a] The values for the duplicate experiment are largely similar and are shown in Table S3. [b] Only the products formed via **3 b** and **3 c** are considered, since the product formed via **3 a** is only formed in negligible amounts (0.6 % of the total product) and its identity is unknown. [c] Average and standard deviation were obtained from two independent DRL experiments. [d] Average and standard deviation were obtained from two chiral HPLC runs after derivatization of product **6** (see Supporting Information).

The determination of the rate constants of intermediates **4** and **5** is somewhat more challenging. As there is a lag phase in the formation of **4′** and **5′** (Figures 5c–e), their relative intensities cannot be fitted by using equation 1. Additionally, since enamines **4** are detected as iminium ions **5** in the ESI‐MS experiments, it is impossible to record the intensity of intermediates **4** and **5** separately. Nonetheless, by simultaneously fitting the relative ion intensities of **5′** (Figures 5c–e) and the isomer ratios (Figures 5f and g), separate rate constants could be derived for intermediates **4** and **5** (Table 1). In fact, without considering separate kinetics for **4** and **5**, we were unable to obtain a reasonable fit of the experimental data, which indicates that both species are necessary to explain the obtained variations in the concentrations.

From the fitted data of the DRL experiments, several direct insights into the reaction can be obtained. Firstly, *k*_2_ of isomer **3 b**, reacting to the minor *S*‐product, was found to be higher than that of **3 c**, which reacts to the major *R*‐product (Table 1). This, obviously, reduces the overall enantiomeric excess (*ee*) of the reaction. The faster reaction could be a result of the steric strain in the **3 b** intermediate that can be released after the formation of enamine **4 b** (see structures and relative energies in Figure 2). Remarkably, the opposite (i.e., higher *k*_2_ for the major isomer) has often been suggested for similar iminium‐catalyzed reactions to explain the higher *e.e*. as compared to the *trans*/*cis* ratio of the primary iminium intermediates.[^4a^, ^7a^] Additionally, notable differences in the kinetics for the different isomers of intermediates **4** and **5** were observed. A relatively rapid 1 : 1 equilibrium of the labeled/unlabeled intermediates was obtained for intermediates **4 b**/**5 b** on the favored pathway, whereas no equilibrium was obtained for intermediates **4 a**/**5 a** and **4 c**/**5 c**. Accordingly, the experimental TIC‐normalized intensity profiles show that isomers **4 a**/**5 a** and **4 c**/**5 c** slowly accumulate in solution whereas **4 b**/**5 b** reach a constant steady‐state concentration (Figure S7). By fitting the data, the individual concentration profiles were obtained for the enamines (**4**) and iminium ions (**5**), which indicate that there is a buildup of iminium ions **5 a** and **5 c** in solution, whereas **5 b** is rapidly hydrolyzed to the product (Figure S8). Such differences in iminium hydrolysis rates have not been described by earlier investigations, and illustrate the level of experimental details that can be obtained using the reported method. In fact, in the original mechanism suggested by Gotoh et al., separate protonation and hydrolysis steps were not even considered.^[12a]^ Nevertheless, the large difference in hydrolysis rates (*k*_4_) is expected to have a negligible effect on the overall enantioselectivity of the reaction studied here. Assuming that CP addition is practically irreversible (see above) and that the values of *k*_−4⋅_[**6**] are close to zero (at short reaction times, the concentrations of the products are negligible), the *ee* is governed by the ratio **3 c** : **3 b** (*trans*/*cis*) and their reaction rates with CP (*k*_2_). We note that the large difference in the rate of hydrolysis between the isomers of intermediate **5** could play a small role in the enantioselectivity when reactions are incomplete, depending on the catalyst concentration (Figure S9).

In order to verify that the method is also suitable to explore the effect of the reaction conditions on the kinetics of isomeric intermediates, the DRL experiment was repeated at a lower temperature (25 °C). Qualitatively, the results were similar to those obtained at 45 °C albeit all intermediates displayed slower kinetics (Figure S11 and Table S4).

### Prediction of Enantioselectivity by Ion Mobility‐Mass Spectrometry

Combining all individual rate constants of the asymmetric reaction allows us to predict the enantiomer ratio of the final product. By using the rate constants of two duplicate DRL experiments (Table 1 and Table S3), we predicted that 86.7 % of the product would occur as the *R*‐enantiomer. The products synthetized under the same conditions contained 88.5 % of the *R*‐enantiomer as determined by chiral HPLC (Table 1). Although, in this case, the accuracy of this prediction is similar to that of the direct estimation from the ratio of iminium ions **3 b** : **3 c**, we note that the DRL approach is the correct one, and is also applicable to reactions where direct estimation from intermediate isomer ratios cannot be used. We emphasize that the excellent ESI‐IM‐MS‐based prediction can be obtained from a ≈30 min sub‐mg scale experiment. By incorporating predictive machine learning methods, this approach can open a perspective for rapid screening of possible new enantioselective reactions and for optimization of the reaction conditions for enantioselective syntheses. This all without the need to perform multiple parallel syntheses, purifications and HPLC analyses.

### Limitations of the Current Method

As indicated above, the novel DRL method provides quantitative insights into the reaction at a level that is unattainable with other methods. Nevertheless, there are some limitations that deserve to be discussed. In an ideal case, when all intermediates can be detected by ESI‐MS (as in the present case), the limitations concern the determination of the initial *k*_1_ rate constants. It is possible to determine a ratio between the *k*_1_ values of the three individual pathways. This ratio, after all, determines to a large extent the isomer ratio of intermediates **3** (Figure 5f). Exact values can, however, not be obtained, as the *k*_1_ constants do not affect the relative intensity evolution of the labeled and unlabeled intermediates. For the same reason, the method is not able to provide values of *k*_−4_ related to the formation of the iminium ions from the product. The presented experiments are performed at low conversion yields, therefore we can assume that *k*_−4_[Product **6**] is close to 0 and *k*_−4_ does not play an important role. The *k*_1_ values could be determined from the reaction monitoring of the interaction of **2** with the catalyst by e.g. NMR spectroscopy.

Another limitation is associated with the ESI‐MS method alone, as not all intermediates can be easily detected by ESI‐MS. Organocatalytic reactions catalyzed by secondary amines such as the one presented here are especially suited because the intermediates can be easily detected as iminium ions or protonated amines. We expect that a similar approach will be possible also for many organometallic reactions for which many studies demonstrated a successful detection of intermediates.^[22]^ If the intermediates were neutral, but could be detected as a charged species (e.g., protonated/deprotonated), the analysis would be still possible. However, for reactions where enantioselectivity is induced by ion pairing^[23]^ or formation of weakly bound complexes,^[24]^ this will probably not be a suitable target for the mass‐spectrometry approach.

## Conclusion

We demonstrated that ion mobility mass spectrometry can be used to predict enantioselectivity in asymmetric reactions. The experiments are quick and can be performed at a (sub‐)mg scale. The analysis of the reaction is based on the determining of the rate constants associated with diastereomeric reaction intermediates along the reaction paths using the delayed reactant labeling approach. We illustrated this method for the organocatalytic Michael addition of cyclopentadiene to *p*‐methoxycinnamaldehyde. We believe that this method can become a useful tool to track the reaction pathways of asymmetric reactions, and can be used to rapidly screen for optimal reaction conditions without the need to perform multiple syntheses. In present, the data require a kinetic modelling that can, however, be machine‐learned when a database of various reactions is available.

## Conflict of interest

The authors declare no conflict of interest.

1

## Supporting information

As a service to our authors and readers, this journal provides supporting information supplied by the authors. Such materials are peer reviewed and may be re‐organized for online delivery, but are not copy‐edited or typeset. Technical support issues arising from supporting information (other than missing files) should be addressed to the authors.

Supporting Information

Supporting Information

## Data Availability

The data that support the findings of this study are available in the Supporting Information of this article.
