# Supplementary material for: Monitoring Reaction Intermediates to Predict Enantioselectivity Using Mass Spectrometry
Source: Angew Chem Int Ed Engl. 2022 Jun 3;61(36):e202205720. doi: 10.1002/anie.202205720 (PMC9544535; doi:10.1002/anie.202205720)
Supplement: Supplementary file 1 — Supporting Information [file ANIE-61-0-s001.pdf]

Angewandte Chemie
Eine Zeitschrift der Gesellschaft Deutscher Chemiker

Supporting Information

Monitoring Reaction Intermediates to Predict Enantioselectivity Using Mass Spectrometry

*R. Hilgers, S. Yong Teng, A. Briš, A. Y. Pereverzev, P. White, J. J. Jansen, J. Roithová**

Contents

Preparation of reaction mixtures	**S3**
Experimental procedures	**S4**
Synthesis of compound 6	**S6**
Derivatization of compound 6 to compound 7	**S6**
Characterization of intermediate 3 (IRPD, NMR and DFT)	**S7**
Experiments on the mass spectrometric detection of intermediate 4	**S10**
Discussion on degradation of catalyst **1**	**S12**
Analytical fitting of $k_{-1}+k_2$	**S13**
Results of the delayed reactant labeling experiment without cyclopentadiene	**S14**
TIC-normalized intensity profiles of isomeric intermediates in the DRL experiment	**S15**
Duplicate of delayed reactant labeling experiment at 45°C	**S16**
Delayed reactant labeling experiment at 25 °C	**S18**
Description and results of Monte-Carlo simulations	**S19**
NMR spectra	**S24**
Cartesian coordinates of DFT-optimized structures	**S32**
References	**S49**

Preparation of reaction mixtures

Reaction mixtures for delayed reactant labeling (DRL) experiments

Reaction mixtures for delayed reactant labeling (DRL) experiments were prepared by mixing stock solutions of (*S*)-(+)-α,α-diphenyl-2-pyrrolidinemethanol trimethylsilyl ether (catalyst **1**), *p*-methoxycinnamaldehyde (compound **2**) and cyclopentadiene in a glass vial to obtain concentrations of 0.167 mM, 3.33 mM and 50 mM, respectively. The reaction mixture was then stirred for 10 min at 45°C, after which *p*-methoxycinnamaldehyde-d_3 (compound **2'**) was added to obtain the final concentrations of 0.1 mM (catalyst **1**), 2 mM (both compounds **2** and **2'**) and 30 mM (cyclopentadiene). Upon addition of compound **2'**, the reaction mixture was infused into the ESI source of the mass spectrometer via a silica capillary by applying a slight overpressure of N_2 (approximately 0.2 bar).

Reaction mixtures for ESI-TIMS-TOF-MS and Helium-tagging photodissociation spectroscopy

Reaction mixtures without cyclopentadiene (CP)

To investigate the isomers of intermediate 3 in detail, experiments were done in absence of CP. Hereto, compound 1 and 2 were mixed in methanol at concentrations of 0.1 mM and 2 mM, respectively. The effect of formic acid on the isomer distribution was checked by preparation of identical samples in presence of 100 mM formic acid. Samples were analyzed after stirring for approximately 1 h at room temperature.

Reaction mixtures with CP

Compound 1, 2 and CP were mixed in methanol at concentrations of 0.1 mM, 2 mM and 50 mM, respectively. The mixtures were stirred for approximately 10 min at room temperature prior to analysis.

Reaction mixtures for studying the reverse reaction

Compound 1 and 6 were mixed in methanol at concentrations of 0.1 mM and 2 mM, respectively. The mixtures were stirred for approximately 10 min at room temperature prior to analysis.

Reaction mixtures for NMR spectroscopy

Reaction mixtures without CP

To investigate the configuration and conformation of the three isomers of intermediate 3, reaction mixtures were prepared without CP. In a glass vial, compound 1 and 2 were mixed at equimolar concentration (75 mM) in ACN-d_3 in presence of 1 M formic acid-d_2. The resulting mixture was left to react at room temperature for 2 or 6 days, prior to NMR analysis. The reaction was performed in acetonitrile instead of methanol, to prevent any C/D-exchange. To allow a fair comparison between the NMR spectra and

extracted-ion mobilograms, the NMR sample was also analyzed using ESI-TIMS-TOF-MS after 100-fold dilution in ACN containing 1 M formic acid.

Experimental procedures

ESI-TIMS-TOF-MS

Trapped ion mobility spectrometry-mass spectrometry experiments were performed by using a TIMS-TOF MS (Bruker Daltonics Inc., Billerica, MA). Ions were generated by electrospray ionization (ESI), with the following settings: capillary voltage 3.5 kV, end plate offset 500 V, dry gas 3.5 L/min, dry temperature 200 °C and nebulizer gas 0.3 bar. TIMS was operated in ultra mode at a range of 0.93-1.26 $V{\cdot}s/m^2$ with an accumulation time of 10 ms. The RF funnel 1 and 2 were set at 400 vpp, multipole RF at 500 vpp and the deflection delta voltage was 70 V. The quadrupole ion energy was 5 eV, transfer time 50 µs and pre-pulse storage time 15 µs.

NMR experiments

NMR experiments were conducted on a Bruker Avance III 400 MHz or Bruker Avance III 500 MHz spectrometer. Depending on the solvent of the sample, the residual solvent peak of chloroform (δ_H = 7.26) or formic acid (δ_H = 8.03) was used as the internal reference.

Helium-tagging photodissociation spectroscopy

Infrared photodissociation spectroscopy (IRPD) experiments were performed by helium-tagging method using the ISORI instrument.[1] The ions were generated under exactly the same conditions as above. The ions of interest were mass-selected and guided with a quadrupole bender and an octopole to a wire quadrupole ion trap operating at 3 K. The ions were trapped using helium buffer gas and after the collisional cooling, the ions formed complexes with helium atoms. The ions in the trap were then irradiated with tunable IR light from OPO. After irradiation, the ions were extracted from the trap, mass-analyzed by a quadrupole and detected by a Daly-type detector. The IRPD spectrum is constructed as $1\text{-}N_i(\nu)/N_{io}$, where $N_i(\nu)$ is a number of helium complexes detected after IR irradiation as a wavenumber ν and N_{io} is a number of helium complexes without irradiation (measured in alternative cycles with IR light blocked).

Chiral HPLC

Chiral HPLC analysis was performed using a Shimadzu LC-20 HPLC system. To determine the enantiomeric excess of the reaction, aldehydes **6** must have been reduced to the corresponding alcohols **7**. To this end, product **6** was dissolved in isopropanol at a concentration of 4 mg/mL and filtered over a 0.22 µm filter. The sample (5 µL) was injected onto a Phenomenex Lux Cellulose-1 column operated at 35 °C.

Heptane/isopropanol (97:3) was used as eluent at a flow rate of 0.5 ml/min. UV detection at 220 and 254 nm was used to monitor the separation of the *R* (t_r =25.0 min) and *S* (t_r =23.6 min) enantiomer of compound **7**.

Computational details

For DFT optimizations, the preferred conformers of all isomers were identified by conformational analysis performed with the PM6 method. The lowest energy conformations were further optimized using the B3LYP functional with the D3 dispersion correction and the 6-31G** basis set as implemented in Gaussian 16.[2] For calculations of IR spectra, scaling factors of 0.985 and 0.952 were used in the region below and above 2000 cm^{-1}, respectively. Calculations of CCS of DFT-optimized structures were performed by using collidoscope.[3]

Determination of rate constants by fitting data of DRL experiments

Summed rate constants for the depletion of intermediates **3**/**3'** (i.e. k_{-1}+k_2) were obtained by analytical fitting the relative intensity of ion **3'** using equation S1[4]:

$$[\mathbf{3'}]_t = [\mathbf{3'}]_{eq}\,(1\text{-}e^{-(k_{-1}+k_2)\cdot t}) \qquad \text{(S1)}$$

In which $[\mathbf{3'}]_t$ is the relative concentration of **3'** (with respect to the sum of **3** and **3'**) at time 't' and $[\mathbf{3'}]_{eq}$ is the relative concentration of **3'** after reaching the equilibrium between labeled and unlabeled intermediates. Note that equation 1 is only valid for intermediates displaying steady-state kinetics. To determine separate values for k_{-1} and k_2, a DRL experiment was performed in the absence of CP which showed that k_{-1} is negligible, therefore we used k_{-1}=0 in further fitting.

For the determination of all other rate constants, the relative ion intensities and isomer ratios were derived using the Euler numerical integration method (see rate equations S1-S6 and the numerical model in an excel sheet in the Supplementary Information). Fitting of the experimental data was performed by adjusting all rate constants in the model except k_{-1} and k_{-4} which were set to zero and k_2, which was obtained analytically from steady state approximation modelling. Monte-Carlo simulations were performed to validate and to refine the fitting (see the description and the statistical models and the fitting procedure in the Supplementary Information). The Monte-Carlo simulations confirmed the validity of the fits and led to minor changes of the rate constants. The values obtained using the manual fitting are presented in the figures and tables of the main article, the Monte-Carlo results are shown in the Supporting Information.

Rate equations used for the determination of rate constants

To determine the rate constants of the individual pathways of the asymmetric reaction, rate equations S2-S7 were used. During fitting of the data with the Euler method, the values of k_{-1} and k_2 were fixed to the outcome obtained via equation 1. Note that in the equations below, the kinetics of the intermediates and product are shown for isomers a as an example. For isomers b and c, individual rate constants were determined using their corresponding concentrations. As CP is added in excess, it is not included in the rate equations.

$$d[1]/dt = -(k_{1a}+ k_{1b}+ k_{1c})[1][2] + k_{-1a}[3a] + k_{-1b}[3b] + k_{-1c}[3c] - k_{-4a}[6a][1] - k_{-4b}[6b][1] - k_{-4c}[6c][1] + k_{4a}[5a] + k_{4b}[5b] + k_{4c}[5c] \quad (S2)$$

$$d[2]/dt = -(k_{1a}+ k_{1b}+ k_{1c})[1][2] + k_{-1a}[3a] + k_{-1b}[3b] + k_{-1c}[3c] \quad (S3)$$

$$d[3a]/dt = k_{1a}[1][2] - k_{-1a}[3a] + k_{2a}[3a] \quad (S4)$$

$$d[4a]/dt = k_{2a}[3a] + k_{-3a}[5a] - k_{3a}[4a] \quad (S5)$$

$$d[5a]/dt = k_{3a}[4a] - k_{4a}[5a] + k_{-4a}[1][6a] \quad (S6)$$

$$d[6a]/dt = k_{4a}[5a] - k_{-4a}[1][6a] \quad (S7)$$

Synthesis of compound 6

Compound 6 was synthesized based on the procedure reported by Gotoh et al.[5] In a round bottom flask compound 1 (0.4 mmol) and compound 2 (4 mmol) were mixed in 8 mL of methanol. After 1 minute of stirring, 12 mmol of cyclopentadiene was added. The reaction was then left stirring under argon for 20 h at 45 °C. The resulting mixture was purified by silica gel column chromatography (AcOEt/hexane 1:20) to obtain product 6. ^{1}H NMR (400 MHz, $CDCl_3$): δ 2.76-2.83 (1H, m), 2.85-2.89 (0.4H, m), 2.89-2.94 (0.6H, m), 2.98-3.02 (1H, m), 2.98-3.02 (0.6H, m), 3.02-3.07 (0.4H, m), 3.78 (3H, s), 4.23-4.33 (1H, m), 6.07-6.10 (0.6H, m), 6.23-6.26 (0.4H, m), 6.26-6.29 (0.4H, m), 6.29-6.33 (0.6H, m), 6.39-6.43 (1H, m), 6.80-6.87 (2H, m), 7.08-7.15 (2H, m), 9.70-9.71 (0.4H, t, J = 2.1), 9.71-9.73 (0.6H, t, J = 2.1).

Derivatization of compound 6 to compound 7 for chiral HPLC

As direct chiral HPLC analysis of compound **6** did not result in sufficient separation, it was derivatized to compound **7** using the procedure reported by Gotoh et al.[1] Hereto, compound **6** (95 mg, 0.41 mmol) was mixed with $NaBH_4$ (1.43 mmol) in methanol (4.8 mL) at 0°C. The mixture was stirred for 20 min at the same temperature, and then quenched by addition of phosphate buffer (0.1 M, pH 7). Subsequently, the organic materials were extracted with ethyl acetate, dried over Na_2SO_4, and concentrated under reduced pressure. The crude mixture (93 mg), was dissolved in ethyl acetate (4.8 mL) containing 10% Pd/C, and

stirred overnight under H_2 atmosphere. The reaction mixture was then filtered over a Celite pad and concentrated under reduced pressure. The residue was purified by preparative thin-layer chromatography (ethyl acetate/heptane 1:3) to yield product 7 with an overall yield of 42%. ^{1}H NMR (400 MHz, $CDCl_3$): δ 0.91-1.03 (1H, m), 1.14-1.81 (7H, m), 1.86-2.12 (3H, m), 2.32-2.42 (1H, m), 3.33-3.52 (2H, m), 3.79 (3H, s), 6.82 (2H, d, *J* = 8.6 Hz), 7.07 (2H, d, *J* = 8.6). e.e. (chiral HPLC): 77 ± 0.2%.

Characterization of intermediate 3 (IRPD, NMR and DFT)

In order to characterize the structures of the isomers of intermediate 3, we first recorded IRPD spectra in order to investigate whether isomerization of the C=C bond would occur in solution. Hereto, IRPD spectra of two reaction mixtures were recorded: a mixture of catalyst 1 and compound 2 (without CP) in methanol, and a similar mixture containing 100 mM formic acid. From ESI-IM-MS experiments, it was observed that the addition of formic acid promoted the formation of isomer 3a (Figure S1a), which could be used as a tool for its identification. The IRPD spectra were compared to the theoretical IR spectra of DFT-optimized structures of four potential double C=C-C=N bond isomers (i.e. E-E, E-Z, Z-E and Z-Z). The theoretical spectra are very similar for the iminium ions with E,E and E,Z configurations, but show that isomerization of the C=C bond (to Z-configuration) would result in several shifts in the IR spectrum, especially around 1600 cm^{-1} (Figure S1b). Since the IRPD spectra of both reaction mixtures (i.e. with and without formic acid) are essentially identical (Figure S1c), we concluded that isomerization of the C=C bond does not occur to any notable extent.

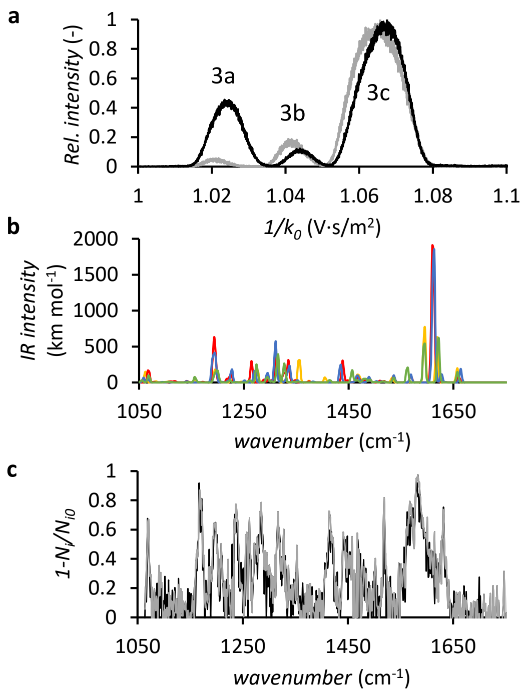

Figure S1 **a**, Extracted-ion mobilograms (*m/z* 470) obtained by ESI-TIMS-TOF-MS analysis of the same solutions as used for the IRPD experiments. The mobilograms of reactions mixtures with and without formic acid (100 mM) are shown in black and gray, respectively. **b**, Theoretical IR spectra of intermediate 3 with E-E (red), E-Z (blue), Z-E (green) and Z-Z (yellow) C=C-C=N configurations. **c**, IRPD spectra of intermediate 3 (*m/z* 470) in reaction mixtures of catalyst 1 and compound 2 in presence (black) and absence (grey) of formic acid.

For further characterization of the isomers of intermediate 3, ^{1}H, TOCSY and NOESY spectra were recorded of a reaction mixture containing equimolar amounts of catalyst 1 and compound 2. As the intensity of iminium ions was very limited in reaction mixtures without additives (data not shown), deuterated formic acid was added to promote iminium ion formation. In the resulting ^{1}H spectrum, three well-resolved peaks were observed in the region around 5-5.5 ppm, where the N-C-H proton of the iminium ions is expected to show up. As not all signals in the ^{1}H spectrum were fully resolved, 1D TOCSY spectra were recorded by selecting the chemical shifts of the N=C-H and N-C-H protons (Figure S20-S25). By combining the ^{1}H and TOCSY spectra with 1D and 2D NOESY spectra, two isomers of iminium ion 3 could be annotated (Figure S2). ESI-IM-MS analysis of the reaction mixture showed a good agreement between the ratio of 3b:3c in the extracted-ion mobilogram and the ratio of the two fully annotated iminium ions in NMR. A third iminium ion was observed as well (δ_H = 5.11 in Fig S2a), but its relative intensity in the NMR spectrum did not match with the relative intensity of 3a in the mobilogram. After a prolonged reaction time (6 days total reaction time), this third iminium ion was substantially increased in abundance. As none of the integrals of the chemical shifts around 0 ppm (i.e. TMS protons) matched with a 9-fold of the integrals of the protons of the third isomer, we concluded that the third iminium ion in the NMR spectrum corresponds to a structure with a hydrolyzed TMS ether, rather than an isomer of 3b and 3c.

a

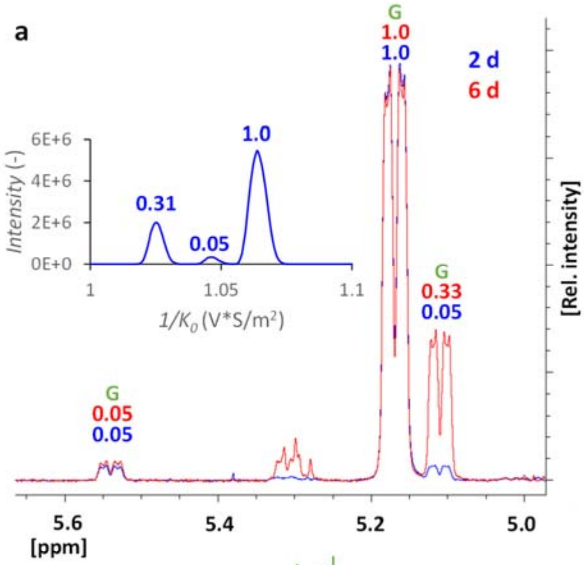

b

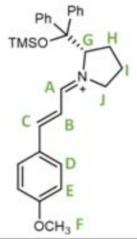

3c

Atom	δ_H	1D TOCSY	NOESY	COSY
A	8.36 (1H, d, J = 10.8)	B, C	B, C, G, TMS, Ph	B, G, J_2
B	6.88 (1H, dd, J = 10.8/15.1)		A, D, J_1, J_2	A, C
C	7.74 (2H, d, J = 15.1)		A, TMS	B
D	7.78 (2H, d, J = 8.8)		B, E	E
E	7.04 (2H, d, J = 8.8)		D, F	D
F	3.85 (3H, s)		E	-
G	5.17 (1H, d, J = 9.4)	H_1, H_2, I_1, I_2, J_1, J_2	A, H_1, H_2, TMS, Ph	H_1, H_2
H_1, H_2	2.42 (m), 2.07 (1H, m)		G, I_1, I_2, Ph	G, I_1, I_2
I_1, I_2	1.25 (1H, m), 1.76 (m)		H_1, H_2, J_1, J_2, Ph	H_1, H_2, J_1, J_2
J_1, J_2	3.76 (1H, m), 2.60 (1H, m)		B, I_1, I_2	A, I_1, I_2
TMS	-0.23 (9H, s)		A, C, G	-
Ph	7.31, 7.35		A	-

c

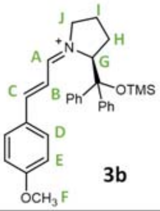

3b

Atom	δ_H	1D TOCSY	NOESY	COSY
A	8.16 (1H, d, J = 10.8)	B, C	C, J_1	B
B	7.00 (dd, J = 10.8/15.1)		D, G, Ph	A, C
C	7.55 (d, J = 15.1)		A, TMS	B
D	7.67		B, E	E
E	6.99		D, F	D
F	3.82 (s)		E	-
G	5.54 (1H, d, J = 9.0)	H_1, H_2, I_1, I_2, J_1, J_2	B, H_1, H_2, Ph	-
H_1, H_2	2.45 (m), 1.80 (m)		G, I_2	-
I_1, I_2	2.20 (m), 1.62 (m)		H_2	-
J_1, J_2	3.64 (1H, m), 2.78 (1H, m)		A	-
TMS	-0.31 (9H, s)		-	-
Ph	7.46		G	-

d

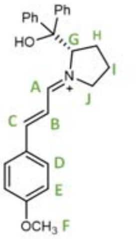

Atom	δ_H	1D TOCSY	NOESY	COSY
A	7.07 (d, J = 10.6)	B, C	C, G	B
B	6.85 (dd, J = 10.6/15.0)		D, J_1	A, C
C	7.22 (d, J = 15.0)		A, D	B
D	7.66		B, C, E	E
E	7.00		D, F	D
F	3.82 (s)		E	-
G	5.11 (1H, d, J = 8.6)	H_1, H_2, I_1, J_1, J_2	H_1, H_2, , Ph	H_1, H_2
H_1, H_2	2.43 (m), 2.16 (1H, m)		G	G
I_1, I_2	2.00 (m)[a]		J_1, J_2	J_1, J_2
J_1, J_2	4.0 (m), 3.79 (m)		B, I_1	I_1
Ph	7.37		G	-

Figure S2 a, Region of the 1H NMR spectrum showing the N-C-H proton (labeled G) of iminium ions formed in an equimolar reaction mixture of catalyst **1** and compound **2** in CD_3CN/FA-d_2. Blue and red spectra correspond to reaction times of 2 and 6 days, respectively. The insert shows the extracted-ion mobilogram (*m/z* 470) of the 2 days incubated sample recorded using ESI-IM-MS after dilution. The numbers above the peaks indicate the integrals relative to the largest peak. **b&c**, Summary of the NMR characterization of two iminium isomers that were annotated as ion **3c** and **3b**. As can be observed in (a), the relative intensities of their NMR signals are in good agreement with those in the extracted-ion mobilogram. Note that the isomer **3a** was impossible to detect by NMR, the third detected species with the increasing relative intensity with time (blue vs. red) is an iminium ion with hydrolyzed TMS ether. **d**, Summary of the NMR characterization of a third iminium ion (with hydrolyzed TMS ether). NOESY and COSY correlations of geminal protons are not shown, and integrals are only displayed for protons showing no or very limited overlap in the 1H NMR spectrum. It should be noted that some expected correlations (especially for COSY) were not detected due to the limited intensity of signals. NMR spectra used for the annotation are shown in Figure S15-S32.

Table S1 Relative energies and collisional cross sections (CCS) of DFT-optimized structures of four configurations intermediate 3. The DFT calculations confirm that the iminium ion is most stable in *trans* C=N configuration, and show that *trans-cis* isomerization of the C=C bond is unfavorable. The relative CCS values of the *trans* and *cis* isomer are in good agreement with the experimental ratio of 3c and 3b obtained by ion mobility spectrometry (1:0.98).

**Structure**	**Rel. energy (kcal/mol)**	**CCS (Å^2)**	**Relative CCS (-)**
3 (*trans* C=N, *trans* C=C)	0	203.181	1.00 (def.)
3 (*cis* C=N, *trans* C=C)	1.70	198.822	0.974
3 (*trans* C=N, *cis* C=C)	3.33	201.856	0.989
3 (*cis* C=N, *cis* C=C)	6.48	198.856	0.974

Experiments on the mass spectrometric detection of intermediate 4

The catalytic cycle of the reaction studied involves enamine 4 and iminium ion 5. After protonation of enamine 4 (during ESI), these two intermediates are both expected to appear at *m/z* 536. The detection of enamine intermediate 4 in ESI-MS experiments could, theoretically, occur after protonation of the nitrogen atom, or via protonation of the β-carbon atom (to iminium ion 5). To investigate which of these two events would occur in practice, we performed DFT optimizations of both N and C-protonated forms of intermediate 4. In addition, we recorded IRPD spectra of the ions with *m/z* 536 in and compared them to theoretical IR spectra of the optimized N and C-protonated intermediates. DFT optimization were performed for both structures with R and S configurations of the C_β atom.

As can be observed from Table S2, the relative energy of N-protonated enamines exceeds their C-protonated analogues by >10 kcal/mol. This suggests that C-protonation occurs exclusively upon ESI of enamines 4. A comparison between the theoretical IR spectra and experimental IRPD spectrum of *m/z* 536 (Figure S3) also indicates that no N-protonated enamines are detected in the ESI-MS experiments. Thus, two possible scenarios remain: i) enamines 4 are rapidly protonated to iminium ions 5 in solution or ii) a mixture of intermediates 4 and 5 is present in solution, but both intermediates are detected as iminium ions 5 upon ESI. As the former hypothesis seems to be incompatible with obtaining a good fit in the modelling of DRL experiments, we conclude that the latter (i.e. conversion of 4 into 5 during ESI) occurs in practice.

Table S2 Relative energies and collisional cross sections (CCS) of DFT-optimized structures of intermediate 4 after C or N-protonation. The DFT calculations indicate that protonation of enamine 4 occurs at the β-carbon, yielding iminium ion 5. The relative CCS values of the R and S isomer are in good agreement with the experimental ratio of 4/5b and 4/5c obtained by ion mobility spectrometry (1:1.013).

Structure	Rel. energy (kcal/mol)	CCS (Å^2)	Relative CCS (-)
(*R*)-4 C-protonated (= (*R*)-5)	0.2	216.855	1.00 (def.)
(*S*)-4 C-protonated (= (*S*)-5)	0	218.965	1.010
(*R*)-4 N-protonated	12.0	N.D	
(*S*)-4 N-protonated	10.0	N.D	

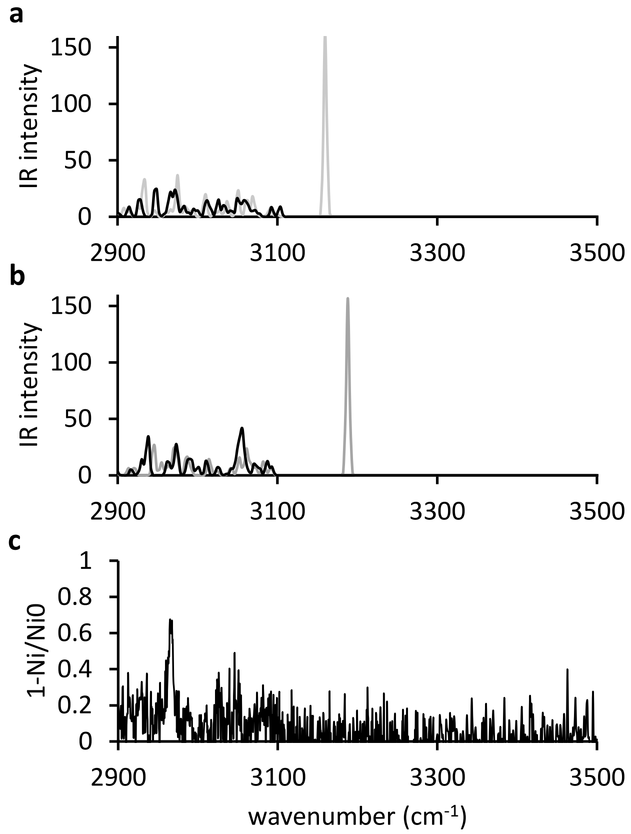

Figure S3 a&b, Theoretical IR spectra of C-protonated (black) and N-protonated (gray) structures of intermediate 4 with the CP moiety added in (*R*)-configuration (A) and (*S*)-configuration (B). **c**, IRPD spectrum of *m/z* 536 in a reaction mixture containing catalyst 1, compound 2 and CP (see page S3 for sample description). The distinct bands around 3160 cm^{-1} (**a**) and 3190 cm^{-1} (**b**) originate from the N-H vibrations of the N-protonated structures, and are not visible in the experimental IRPD spectrum (**c**).

Discussion on degradation of the catalyst TMS ether

As can be observed from Figure 2a, ions of *m/z* 236 were detected in the ESI-MS spectrum of the reaction mixture. This ion corresponds to the protonated catalyst **1** after the loss of TMS-OH (neutral loss = 90 Da). This elimination occurs during the electrospray ionization, because the ratio of the ions with m/z 236 and 326 is constant over time. In agreement, collision induced dissociation of mass-selected **1**H^+ (*m/z* 326) yields dominantly the ions with *m/z* 236.

In addition, relatively small intensities of *m/z* 398 and 401 were detected. These *m/z* values correspond to iminium ions formed between reactant **2**(‘) and catalyst **1** of which the TMS ether has been hydrolyzed. In the mass spectrum recorded with the tims-cell disabled, an ion with *m/z* 254 is detected (loss of 72 Da compared to the intact catalyst), confirming that the hydrolyzed catalyst is indeed present. Hydrolysis of TMS ethers in Jørgensen-Hayashi type catalysts has been reported previously by others.[6] The iminium ions formed via the free -OH catalysts have been suggested to exist in equilibrium with oxazolidine species, via intramolecular cyclization, resulting in a diminished reactivity.[7] We are currently investigating this phenomenon and the effect on the kinetics of the intermediates in more detail, using the novel method presented in this study.

Analytical fitting of $k_{-1} + k_2$

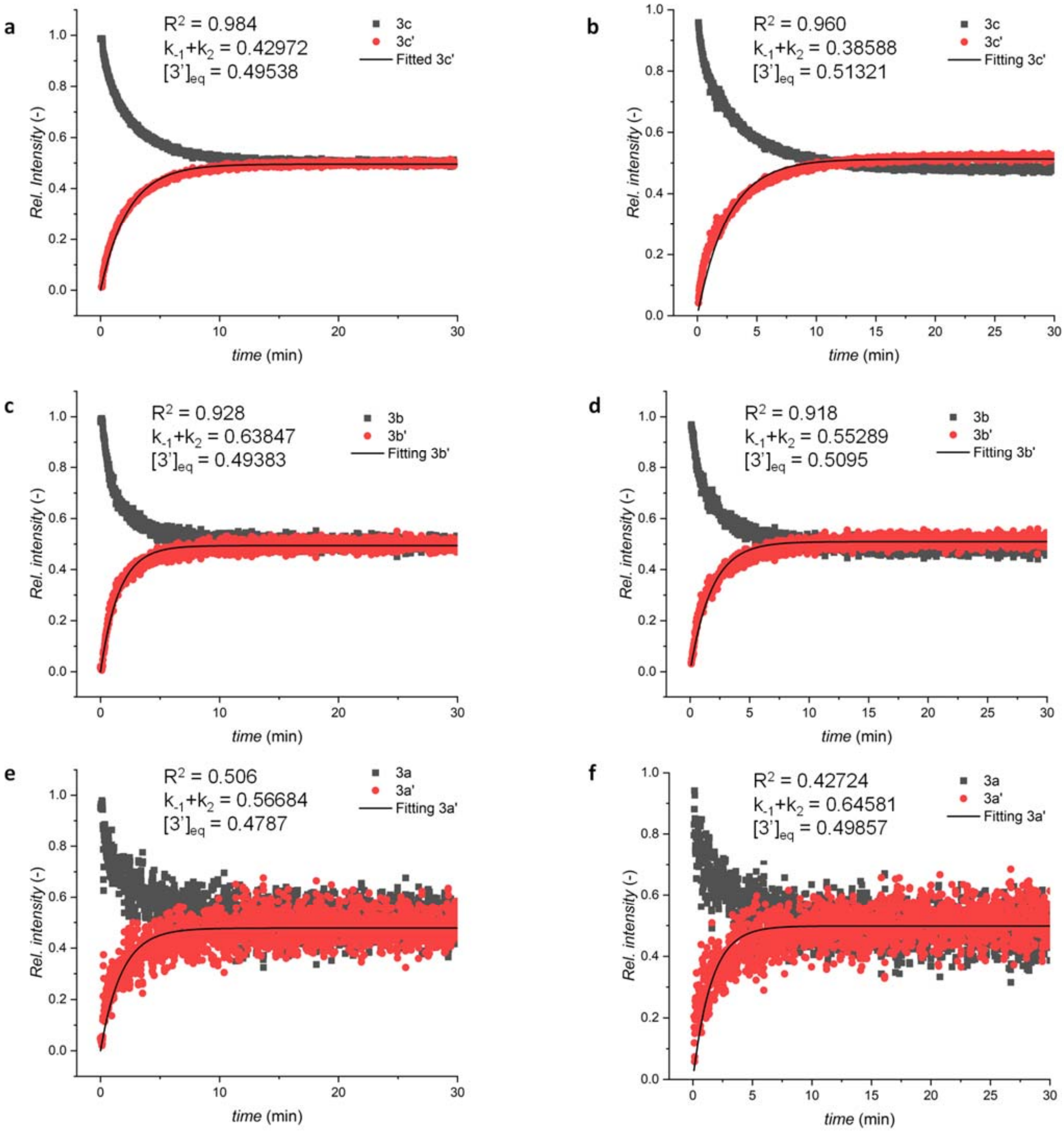

Figure S4 Ratios of 3c/3c' (**a&b**), 3b/3b' (**c&d**), and 3a/3a' (**e&f**) in two (duplicate) delayed reactant labeling experiments. Relative intensities of labeled and unlabeled intermediates are shown in red and black, respectively. The black lines represent the fits of 3c' according to equation 1 (see method section). The values of a and b in the boxes represent the fitted relative concentrations of the labeled ions at equilibrium, and the summed rate constants of the depletion of the intermediates (i.e. $k_{-1} + k_2$).

Results of the delayed reactant labeling experiment without cyclopentadiene

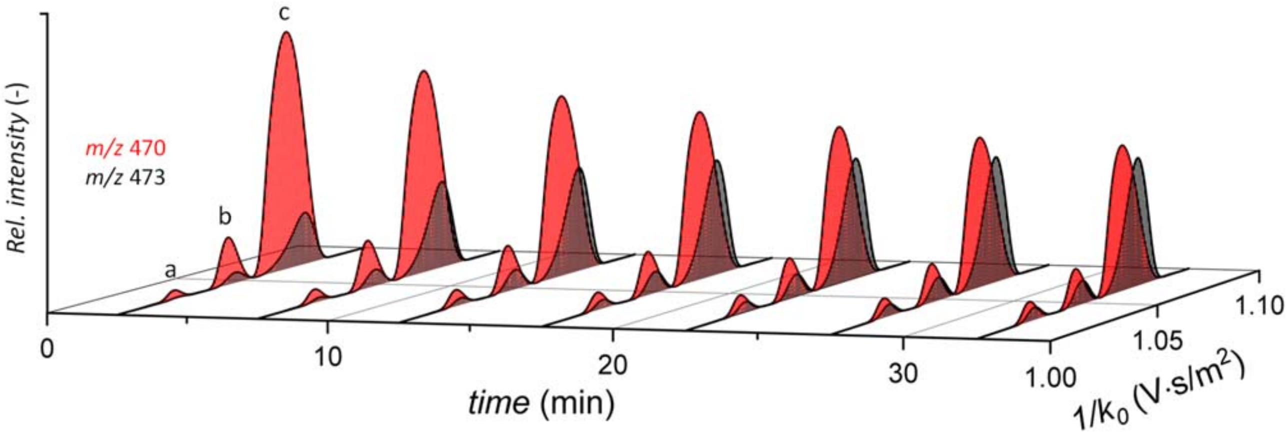

Figure S5 TIC-normalized extracted ion mobilograms illustrating the time evolution of individual intermediate isomers for 3 & 3' in a DRL experiment without CP. The equilibrium formation between labeled and unlabeled species is slow in comparison to the situation with CP (see Figure 4). The slower depletion of the iminium intermediates in absence of CP indicates that the rate of hydrolysis of intermediate 3 (to the reactant and catalyst) is slow in comparison to the nucleophilic attack by CP. Interestingly, the relative abundance of isomer a seems to increase over time. This might indicate slow isomerization of between the intermediate 3 isomers. As this process is slow, it is not observed in presence of CP, as all isomers rapidly react with CP.

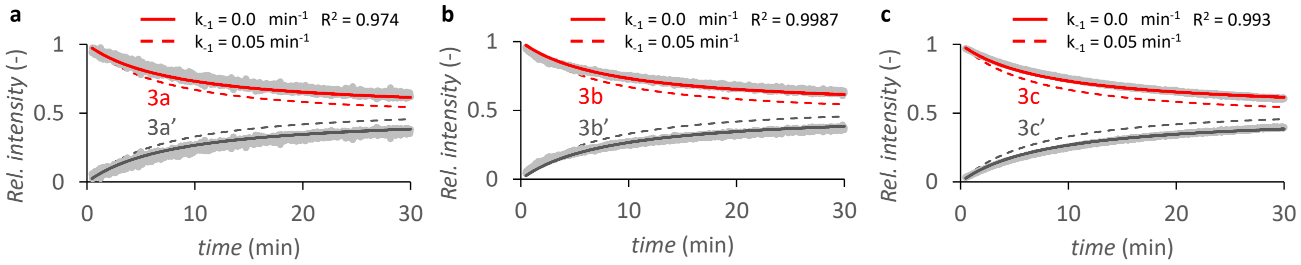

Figure S6 Time evolution of relative ion intensities of labeled vs. unlabeled iminium ions in the delayed reactant labeling experiment without CP for isomer 3a (**a**), 3b (**b**) and 3c (**c**). Experimental relative ion intensities are shown in light gray. Theoretical relative concentrations obtained by modelling (Euler method) are shown in red for ions **3** and in dark gray for ions **3'**. The displayed R^2 values are the squared correlation coefficients obtained for the relative intensity of the labeled ions. The dashed lines (with k_{-1}) are merely included to illustrate that increasing k_{-1} results in bad fitting.

TIC-normalized intensity profiles of isomeric intermediates in the delayed reactant labeling experiment

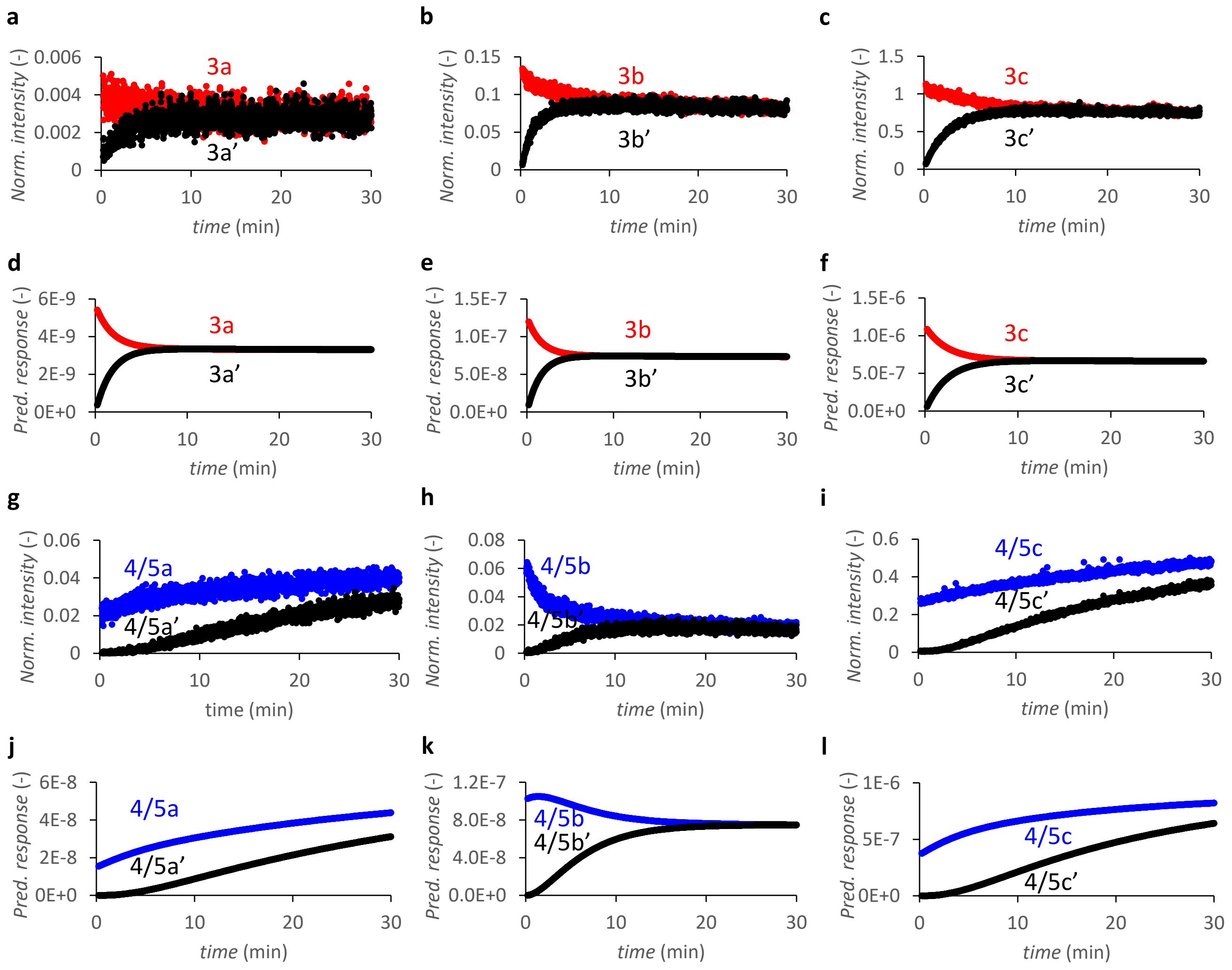

Figure S7 TIC-normalized intensity profiles of labeled and unlabeled intermediate ions in the DRL experiment at 45°C (**a-c** and **g-i**) and their predicted response by using the rate constants displayed in Table 1 (**d-f** and **j-l**). The predicted responses (Y-axes) in **d-f** and **j-l** were calculated by summing the predicted concentrations of ions 4 and 5 with a correction factor of 0.025 for ion 4 to account for its lower ionization efficiency.

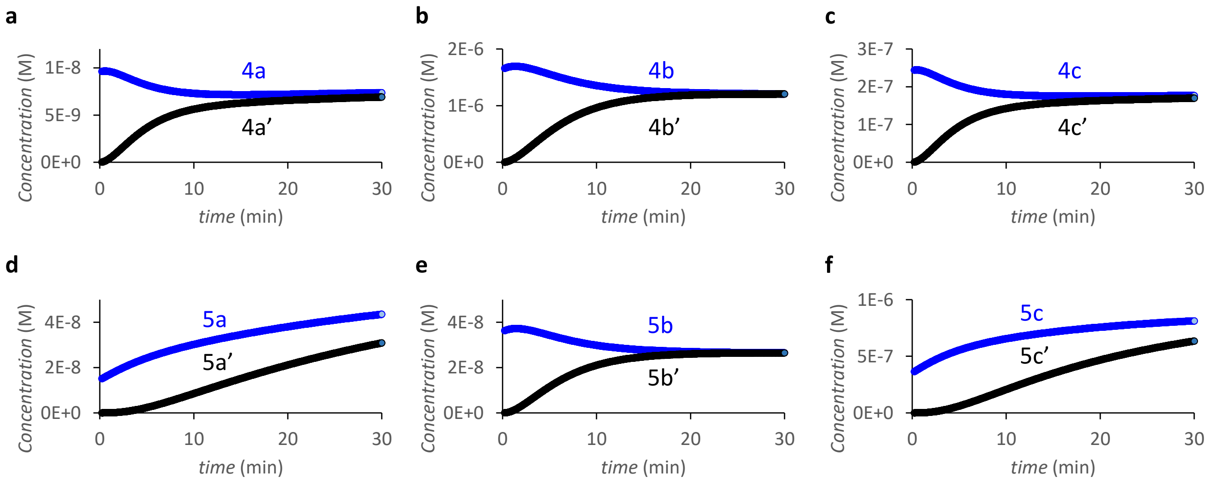

Figure S8 Predicted concentrations of all isomers of intermediates 4 and 5 during the DRL experiment at 45°C. As can be observed, within the time frame of the experiment, iminium ions 5a and c are predicted to slowly accumulate in solution, whereas 5b displays steady-state kinetics.

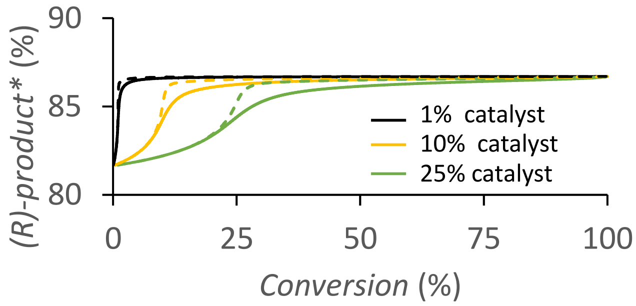

Figure S9 Predicted percentage of the major (*R*) enantiomer for various catalyst loadings at 45 °C. The solid lines were constructed using the average rate constants of the two duplicate experiments (see Table 1 and Table S3). For the construction of the dashed lines, k_4 of the major pathway was lowered to the average of k_4 of the minor pathway (0.06 min^{-1}). The graph illustrates that k_4 has no effect on the enantiomer ratio at full conversion, and only a small effect at low conversions. *Note that for the calculation of the enantiomer ratio, we assume that intermediates **4** and **5** are eventually fully hydrolyzed to product **6** (i.e. the Y-axis corresponds to the sum of **4c**, **5c** and ***R*-6**).

Duplicate of delayed reactant labeling experiment at 45°C

In order to check the reproducibility of the novel method, a duplicate of the DRL experiment was performed. Comparing Figure S10 with Figure 5 and Table 1 with Table S3 indicates that only small deviations are observed between the two duplicate experiments.

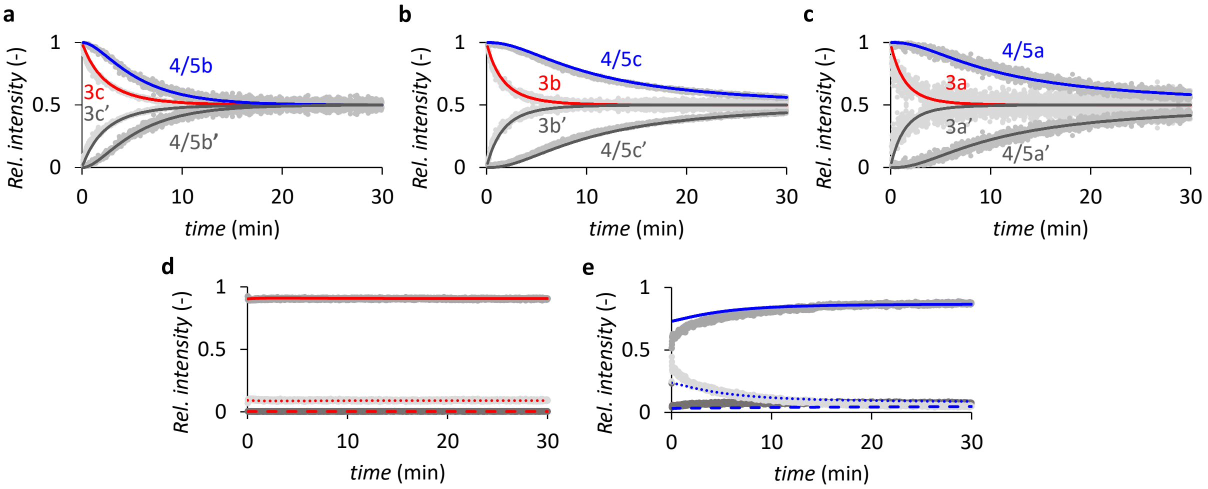

Figure S10 a-c, Replication of the DRL experiment displayed in Figure 5, showing time evolution of relative ion intensities of labeled vs. unlabeled intermediates in the delayed reactant labeling experiment for the three pathways shown in Figure 3. Experimental relative ion intensities are shown in light gray and dark gray for ion 3 & 3' and in gray for 4/5 & 4'/5'. Relative intensities obtained by modelling (see Table S3 for rate constants) are shown for ions 3 & 3' (red lines) and 4/5 & 4'/5' (blue). **d&e**, Time evolution of the relative ion intensities of the three isomers of intermediate 3 (**d**) and intermediate 4/5 (**e**). Both graphs display the experimental relative ion intensity of isomers a (dark grey), isomers b (light gray) and isomers c (gray), as well as the relative ion intensity obtain by modelling: isomer a (dashed line), isomer b (dotted line) and isomer c (solid line). The blue curves were constructed by summing the concentrations of enamine 4 and iminium ion 5, using a correction factor of 0.035 to account for the lower ionization efficiency of the enamine.

Table S3 Rate constants for individual isomeric intermediates obtained by simultaneously fitting the relative intensities of labeled and unlabeled intermediates (Figure S10a-c) and relative intensities of intermediate isomers (Figure S10d&e).

**Pathway**	**k_1**	**k_{-1}**	**k_2**	**k_{-2}**	**k_3**	**k_{-3}**	**k_4**
	M^{-1} min^{-1}	min^{-1}	min^{-1}	min^{-1}	min^{-1}	min^{-1}	min^{-1}
3C→4/5B	1.76	0.0	0.39	0.025	0.25	0.0	8
3B→4/5C	0.25	0.0	0.55	0.030	0.30	0.0	0.05
3A→4/5A	0.01	0.0	0.65	0.025	0.30	0.0	0.03

Delayed reactant labeling experiment at 25 °C

As the results presented in the main article are all obtained using the same reaction conditions, we repeated the delayed reactant labeling experiment at a temperature of 25 °C (instead of 45°C), to verify that the novel DRL method can also be used to investigate the effect of reaction conditions on the kinetics of individual reactions steps. An identical experimental procedure was used, the only adaptation being the temperature.

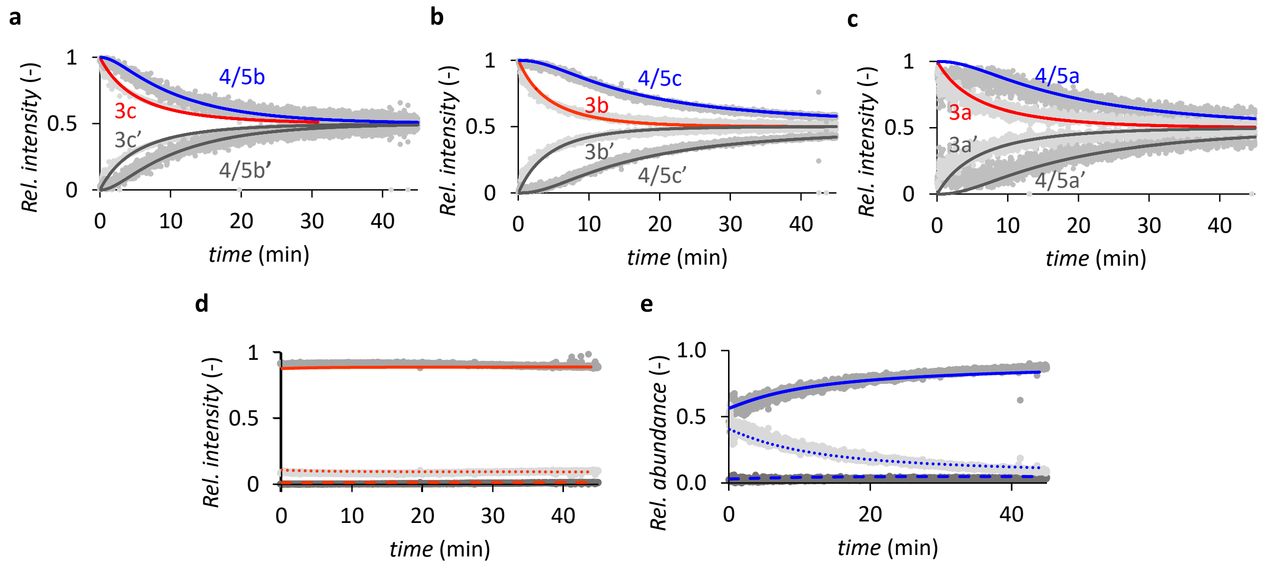

Figure S11 a-c, Time evolution of relative ion intensities of labeled vs. unlabeled intermediates in the delayed reactant labeling experiment for the three pathways shown in Figure 3. Experimental relative ion intensities are shown in light gray. Relative intensities obtained by modelling (see Table S4 for rate constants) are shown for ions **3** (red lines) **3'** (dark gray), **5** (blue) and **5'** (dark gray). **d&e**, Time evolution of the relative ion intensities of the three isomers of intermediate 3 (**d**) and intermediate 4/5 (**e**). Both graphs display the experimental relative ion intensity of isomers A (dark grey), isomers B (light gray) and isomers C (gray), as well as the relative ion intensity obtain by modelling: isomer A (dashed line), isomer B (dotted line) and isomer C (solid line). The blue curves were constructed by summing the concentrations of enamine 4 and iminium ion 5, using a correction factor of 0.04 to account for the lower ionization efficiency of the enamine.

Table S4 Rate constants for individual isomeric intermediates at 25°C obtained by simultaneously fitting the relative intensities of labeled and unlabeled intermediates (Figure S11a-c) and relative intensities of intermediate isomers (Figure S11d&e).

**Pathway**	k_1	k_{-1}	k_2	k_{-2}	k_3	k_{-3}	k_4	**Predicted % of product 6**[a]
	M^{-1} min^{-1}	min^{-1}	min^{-1}	min^{-1}	min^{-1}	min^{-1}	min^{-1}	
3C→4/5B	0.75	0.0	0.13	0.015	0.13	0.0	2.5	87.2 (*R*)
3B→4/5C	0.11	0.0	0.18	0.025	0.18	0.0	0.015	12.8 (*S*)
3A→4/5A	0.01	0.0	0.10	0.015	0.16	0.0	0.035	-

[a]Only the products formed via 3b and 3c are considered, since the product formed via 3a is only formed in negligible amounts (0.7% of the total product) and its identity is unknown.

Description and results of Monte-Carlo simulations

Two types of distinct Monte Carlo (MC) simulations were carried out for the kinetic parameters and the initial experimental conditions (i.e. concentrations) of the asymmetric reaction (see Fig S12a). A sequential MC approach was used in an attempt to fine-tune fitting of the kinetic parameters, and the fitted kinetic model and resulting residuals from the final MC sampled points were subsequently tested using a z-test for statistical significance in fitting. The parametric importance for each kinetic parameters of different product pathways were then deduced using the change in Pearson's correlation coefficient (R) relative to the change in parametric values. For the analysis of robustness in the model with respect to initial experimental conditions, a separate MC simulation was carried out and the initial conditions were analyzed with the model output (product yields) using principal component analysis (PCA). For visualization, the scores of PCA were projected on the loadings with Pearson's correlation coefficient as color hues. The stability and effects of the multivariate samples can be interpreted from a biplot.

For the main product pathway (3C→4/5B), the input distribution of the kinetic parameters for MC simulation is shown in Figure S12b. The kinetic parameters with high variances are k_3 and k_4, where the high variance of the former is caused by the indefinite in the starting point of the experiment (i.e. t_d), while the latter is due to significantly lowered concentration in the process of asymmetric reaction. MC sampled points were observed to give significantly higher absolute residual than the fitted kinetic model using sequential MC simulation for kinetic parameters (see Fig S12c). The implication is that further tuning of the kinetic parameters will not significantly improve the model's statistical performance ($z = 0.0944$, $p<0.001$). Further analysis using kernel density estimation of the principal components of the kinetic parameters (Fig S12d) shows that the fitted model has been densely search around its local space, ensuring that the model is situated within an optimal point.

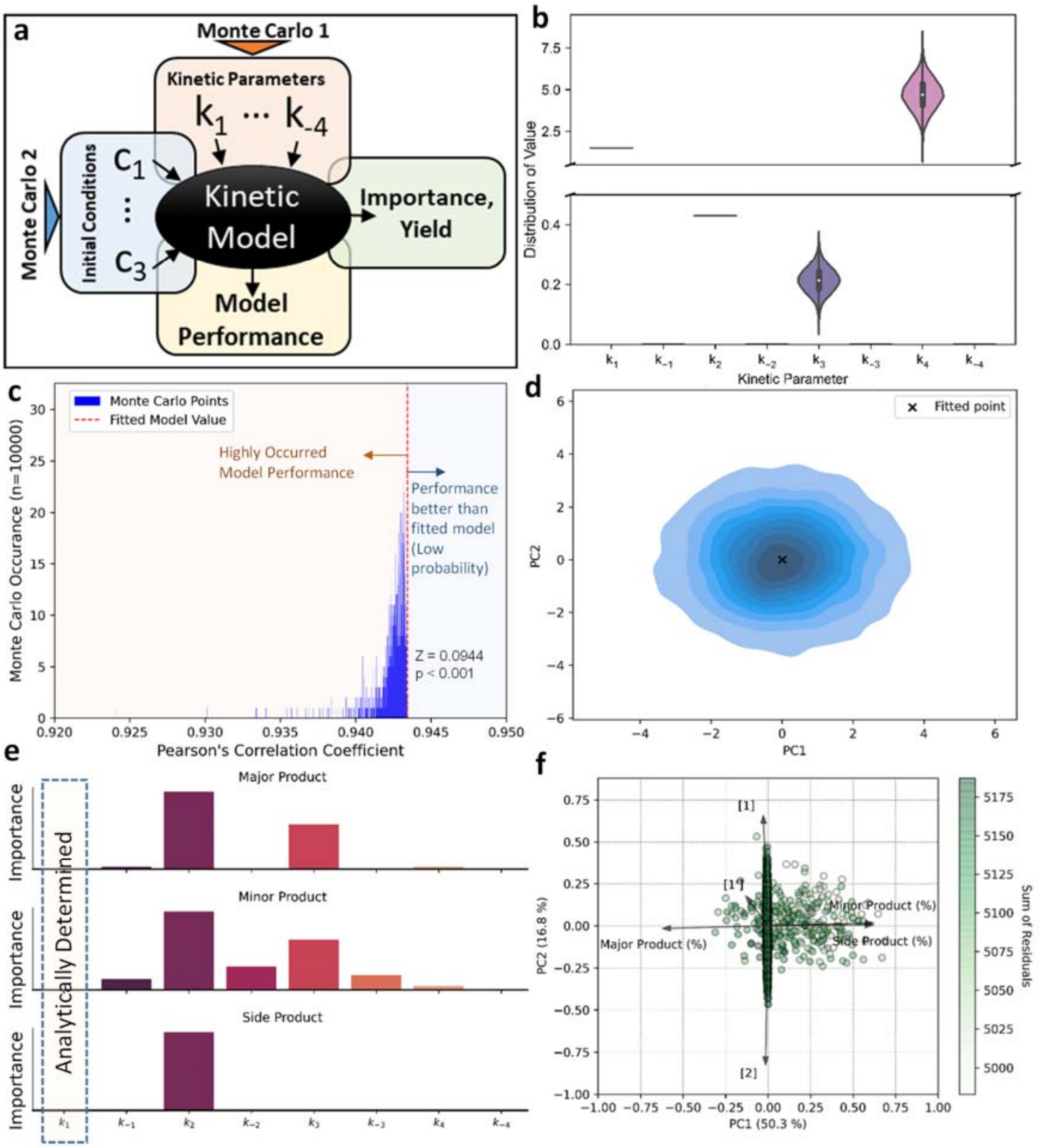

Figure S12 a, Concept of using two Monte Carlo strategies in analysis of asymmetric reaction. **b,** Monte Carlo distribution value of kinetic constants for the major product. **c,** Monte Carlo 1 resulting Pearson's correlation coefficient with respect to fitted kinetic model. **d,** Kernel density plot of the principal component scores of the kinetic parameters. **e,** Importance plot for the kinetic constants of the major product, minor product and side product in Monte Carlo simulation 2. **f,** Principal component analysis plot for Monte Carlo simulation 2 for the robustness of experimental initial conditions on kinetic model.

From analyzing the parametric importance of MC points in the second Monte Carlo simulation (see Figure S12e), it can be derived that the rate constants of the forward reaction are more important than those corresponding to the reverse reaction. An additional MC simulation was carried out on the initial experimental conditions (i.e. concentrations) while analyzed with the product yields in a principal component analysis scores projected on loadings. In Figure S12f, the initial experimental conditions for compound 1, 1' and 2 are represented in the PC2 axis, while the product yield (major, minor and side products) was represented in the PC1 axis. The clump of dark green points in the middle with stationary value in PC 1 with majority of MC sampled points imply that initial conditions were not significantly affecting the product yields of the asymmetric reaction. This demonstrates that uncertainty in initial experimental conditions does not significantly impact the model prediction. The inverse direction of the loading arrows for major product with respect to minor and side product suggests a competing mechanism between product pathways.

Using the same method as Monte Carlo simulation 1, two additional analyses were performed with slightly different sets of restrictions (see captions of Table S5-S6). The starting points of the Monte-Carlo simulations was determined using analytical fitting. From the results (Table S5-S6 and Figure S13-S14) it can be observed that the k_{-1} parameter within the reaction system is not crucial in giving accurate estimation of relative intensities over time. This confirms the findings from Figure S12e, showing that k_{-1} has low importance in characterizing the asymmetric reaction system. The simulations also demonstrate that the fundamental kinetic parameters that characterizes the asymmetric reaction system is mainly k_2 and k_3.

Table S5 Rate constants refined using sequential Monte-Carlo simulations, with the following restrictions: k_{-1} fixed at 0.025, k_2 fixed to values of Table 1, k_{-2} and k_{-3} fixed at 0. Other rate constants were optimized by the sequential Monte-Carlo simulation.

Pathway	k_1	k_{-1}	k_2	k_{-2}	k_3	k_{-3}	k_4
	M^{-1} min^{-1}	min^{-1}	min^{-1}	min^{-1}	min^{-1}	min^{-1}	min^{-1}
**3c→4/5b**	1.50	0.024	0.41	0.0	0.21	0.0	6.70
**3b→4/5c**	0.25	0.026	0.61	0.0	0.44	0.0	0.05
**3a→4/5a**	0.01	0.022	0.54	0.0	0.27	0.0	0.04

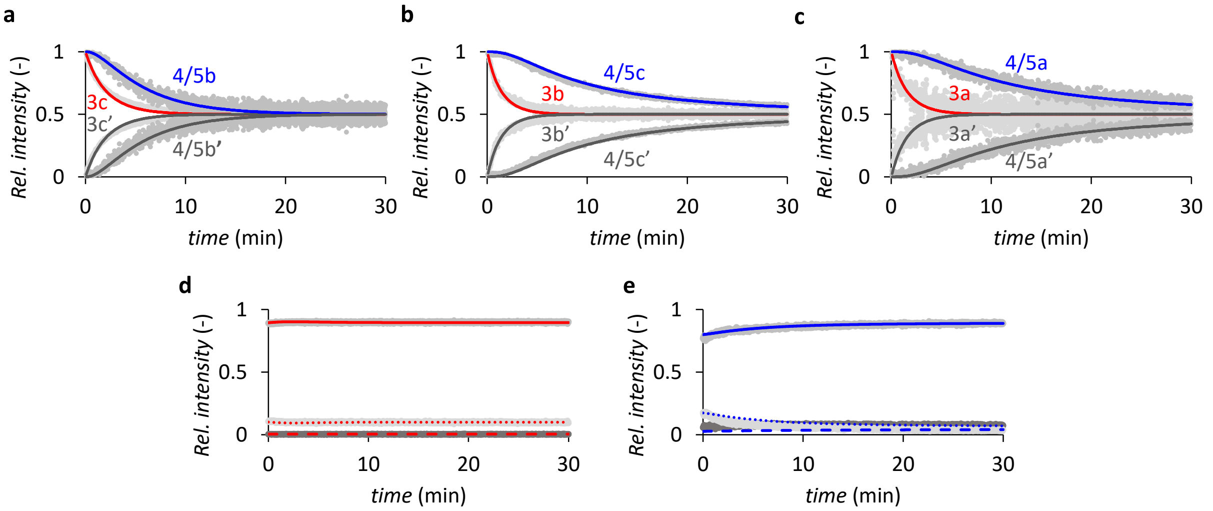

Figure S13 a&b, TIC-normalized extracted ion mobilograms illustrating the time evolution of individual intermediate isomers for **3** & **3'** (**a**), and **5** & **5'** (**b**). **c-e**, Time evolution of relative ion intensities of labeled vs. unlabeled intermediates in the delayed reactant labeling experiment for the three pathways shown in Fig 3. Experimental relative ion intensities are shown in light gray. Relative intensities obtained by modelling (see Table S5 for rate constants) are shown for ions **3** (red lines) & **3'** (dark gray) and **5** (blue) & **5'** (dark gray). **f&g**, Time evolution of the relative ion intensities of the three isomers of intermediate **3** (**f**) and intermediate **5** (**g**). Both graphs display the experimental relative ion intensity of isomers a (dark grey), isomers b (light gray) and isomers c (gray), as well as the relative ion intensity obtain by modelling: isomer a (dashed line), isomer b (dotted line) and isomer c (solid line). The blue curves were constructed by summing the predicted concentrations of enamine **4** and iminium ion **5**, using a correction factor of 0.021 to account for the lower ionization efficiency of the enamine.

Table S6 Rate constants refined using sequential Monte-Carlo simulations, with the following restrictions: k_1 and k_2 fixed to values of Table 1, k_{-1}, k_{-2} and k_{-3} fixed at 0. Other rate constants were optimized by the sequential Monte-Carlo simulation.

**Pathway**	**k_1**	**k_{-1}**	**k_2**	**k_{-2}**	**k_3**	**k_{-3}**	**k_4**
	M^{-1} min^{-1}	min^{-1}	min^{-1}	min^{-1}	min^{-1}	min^{-1}	min^{-1}
**3c→4/5b**	1.50	0.0	0.43	0.0	0.20	0.0	6.67
**3b→4/5c**	0.25	0.0	0.64	0.0	0.38	0.0	0.06
**3a→4/5a**	0.01	0.0	0.57	0.0	0.27	0.0	0.02

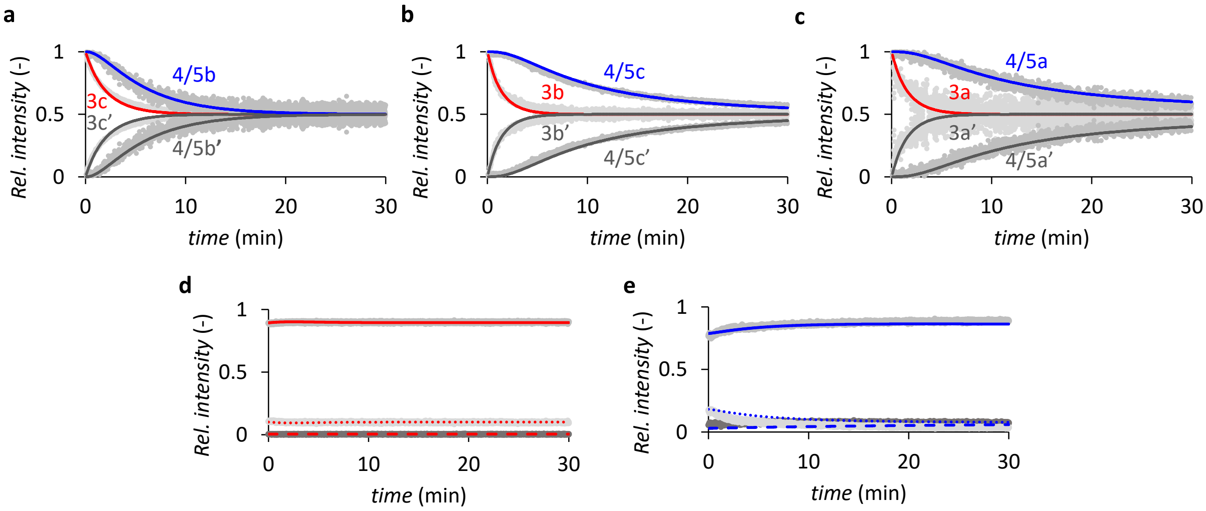

Figure S14 a&b, TIC-normalized extracted ion mobilograms illustrating the time evolution of individual intermediate isomers for **3** & **3'** (**a**), and **5** & **5'** (**b**). **c-e**, Time evolution of relative ion intensities of labeled vs. unlabeled intermediates in the delayed reactant labeling experiment for the three pathways shown in Fig 3. Experimental relative ion intensities are shown in light gray. Relative intensities obtained by modelling (see Table S6 for rate constants) are shown for ions **3** (red lines) & **3'** (dark gray) and **5** (blue) & **5'** (dark gray). **f&g**, Time evolution of the relative ion intensities of the three isomers of intermediate **3** (**f**) and intermediate **5** (**g**). Both graphs display the experimental relative ion intensity of isomers a (dark grey), isomers b (light gray) and isomers c (gray), as well as the relative ion intensity obtain by modelling: isomer a (dashed line), isomer b (dotted line) and isomer c (solid line). The blue curves were constructed by summing the predicted concentrations of enamine **4** and iminium ion **5**, using a correction factor of 0.021 to account for the lower ionization efficiency of the enamine.

NMR spectra

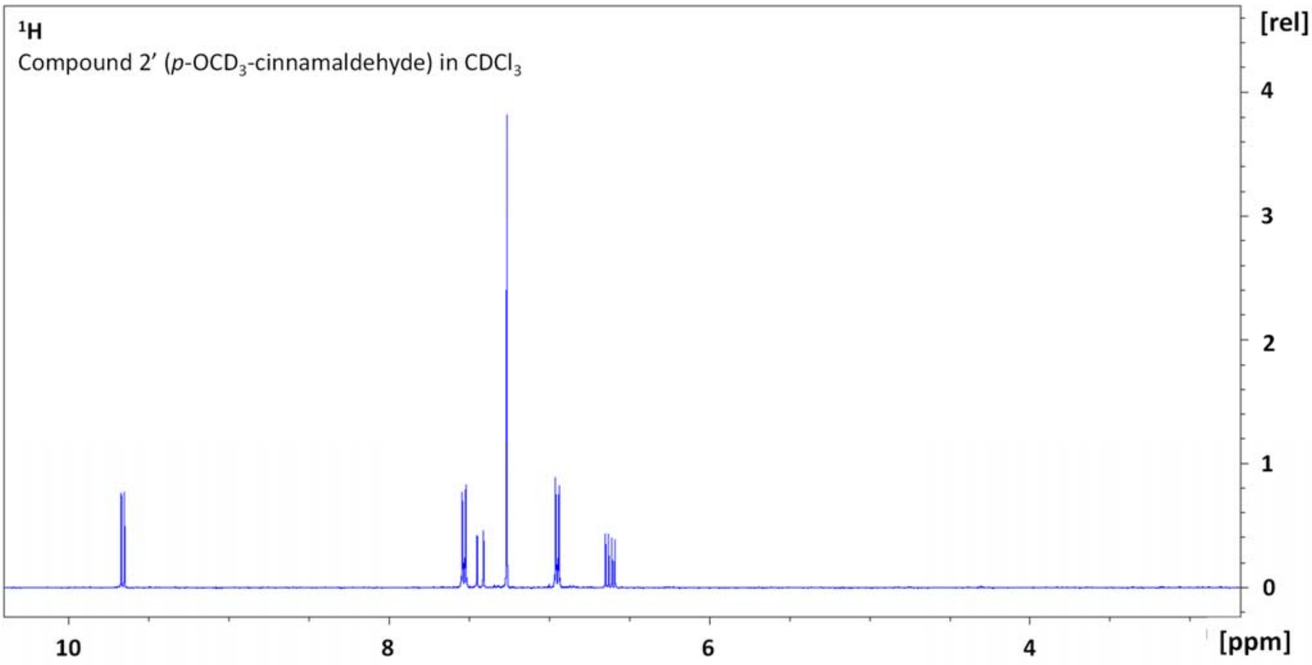

Figure S15 ^{1}H NMR spectrum (400 MHz, $CDCl_3$) of *p*-OCD_3-cinnamaldehyde (compound 2') used in DRL experiments.

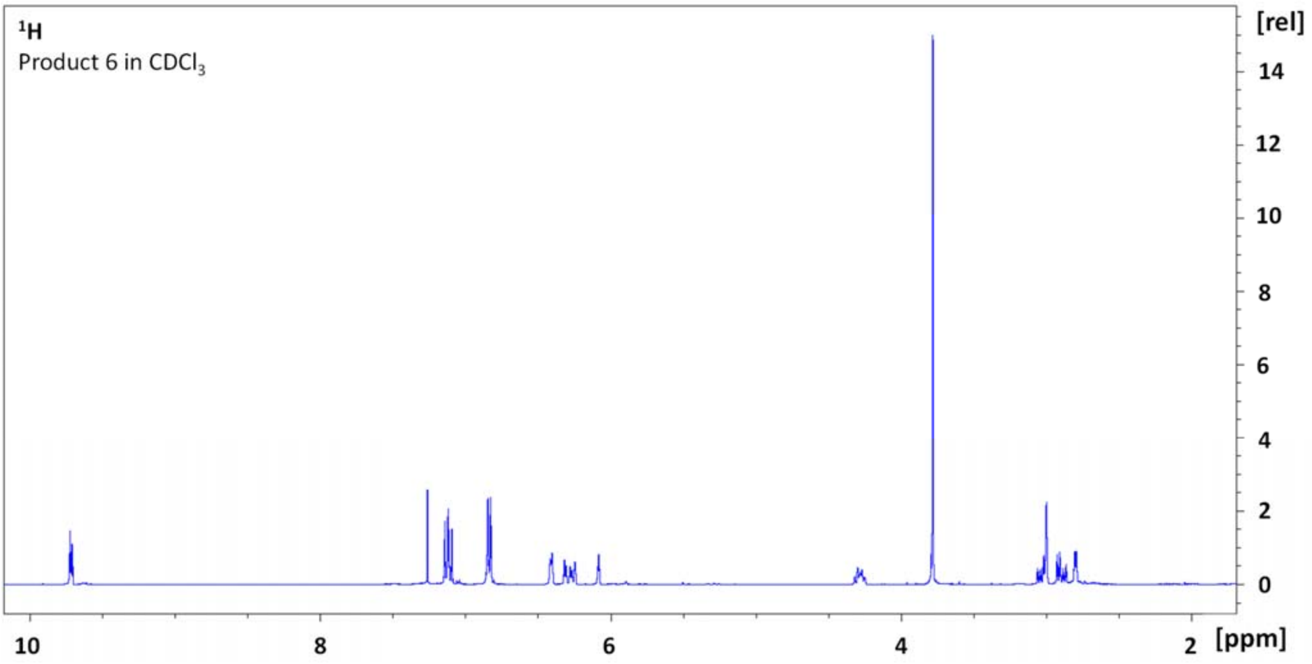

Figure S16 ^{1}H NMR spectrum (400 MHz, $CDCl_3$) of product 6.

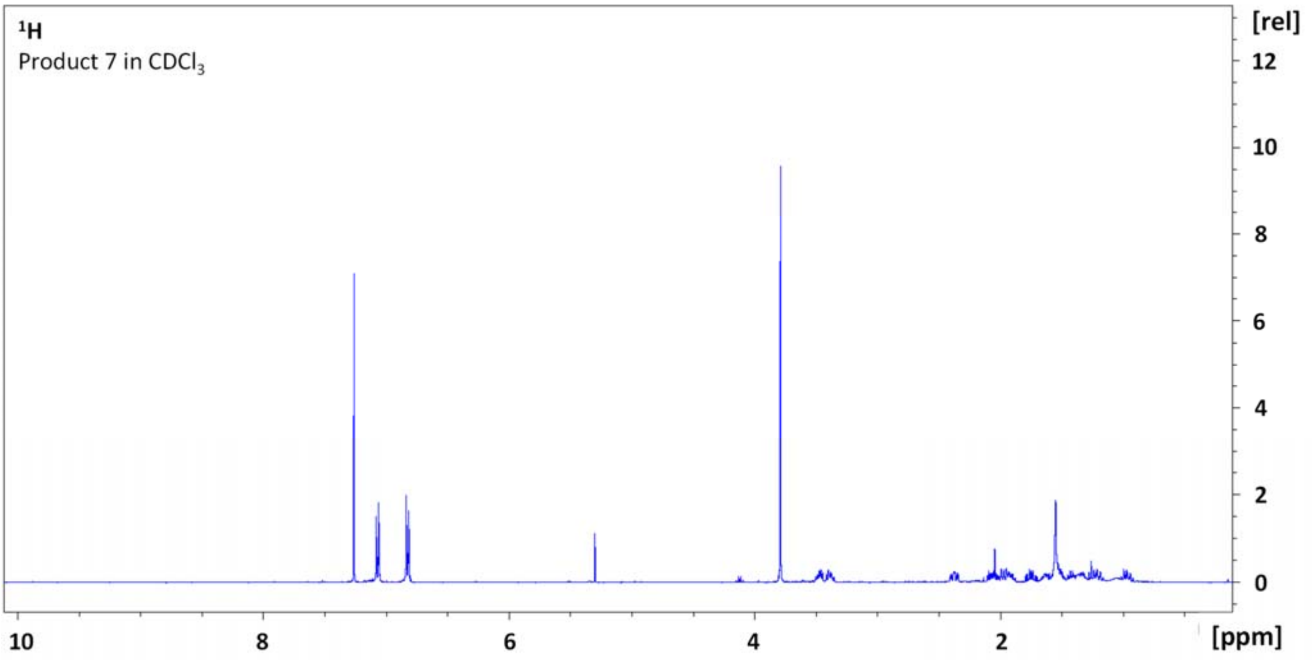

Figure S17 ^{1}H NMR spectrum (400 MHz, $CDCl_3$) of product 7.

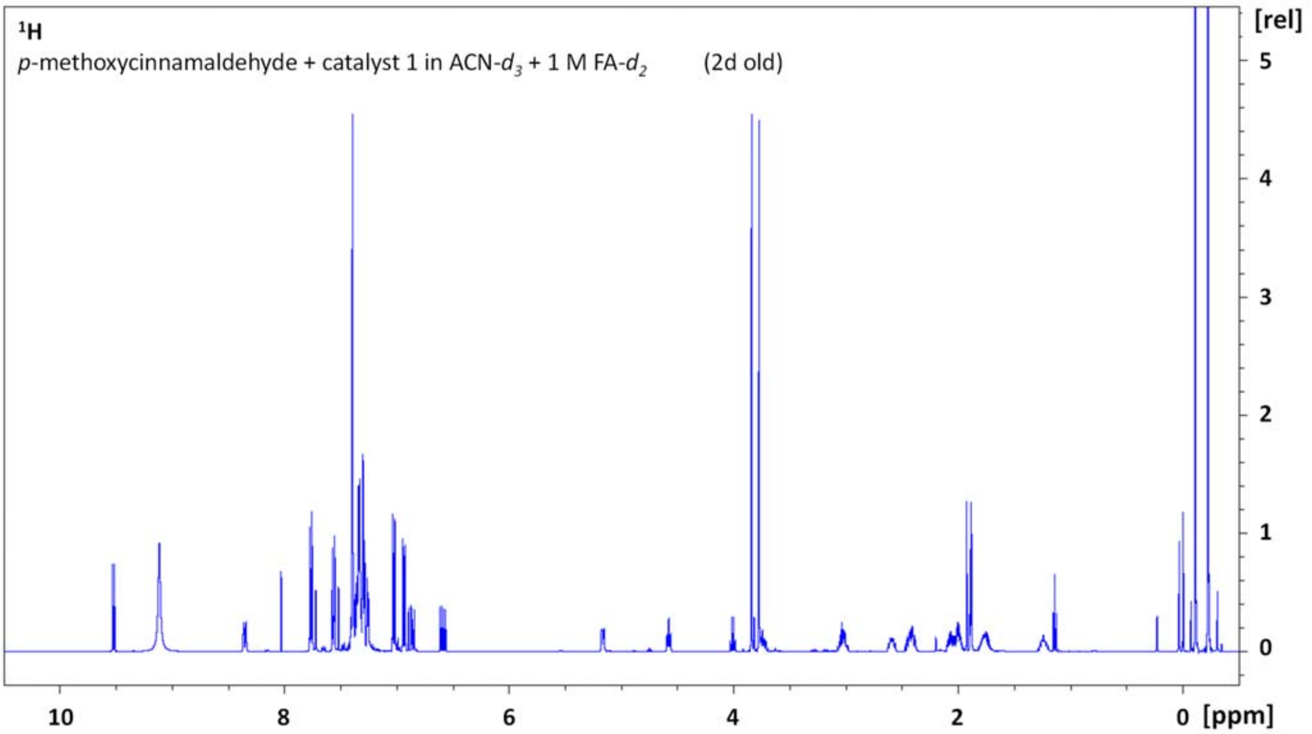

Figure S18 ^{1}H NMR spectrum (500 MHz, CD_3CN/FA) of an equimolar mixture of catalyst 1 and compound 2 after 2 days at room temperature.

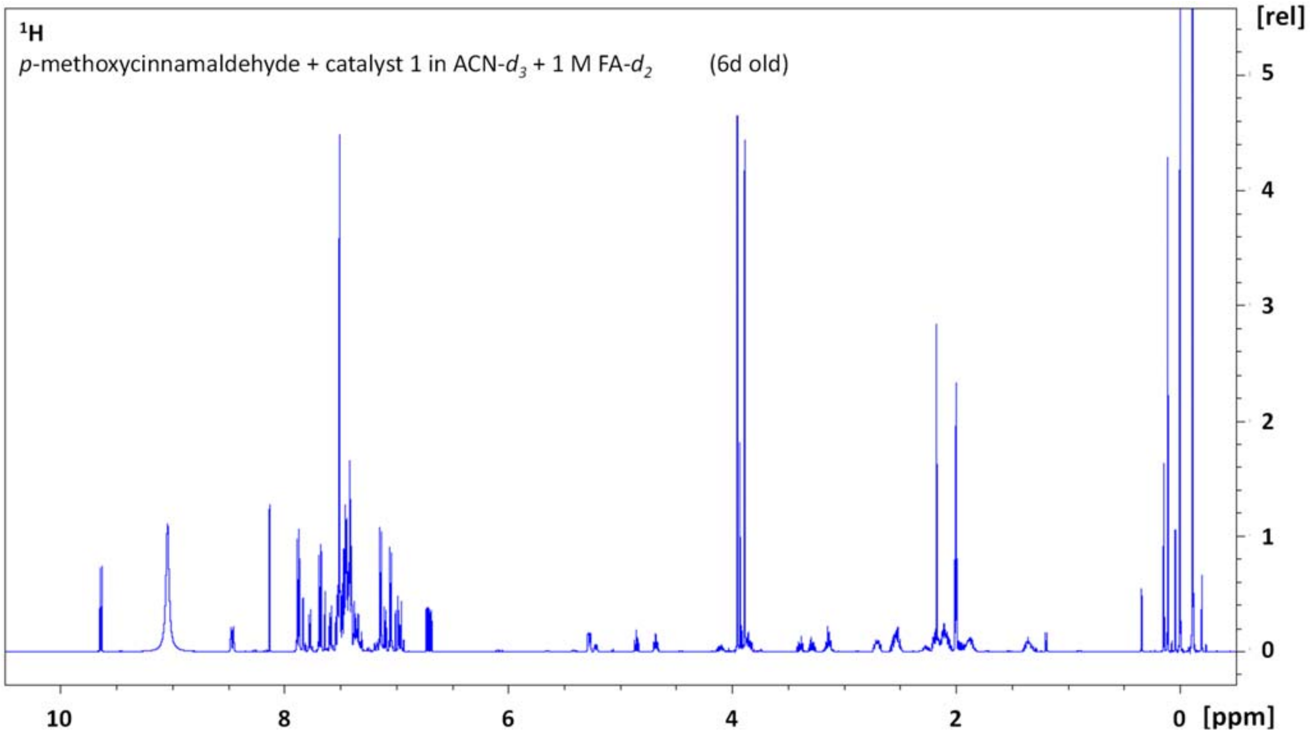

Figure S19 ^{1}H NMR spectrum (500 MHz, CD_3CN/FA) of an equimolar mixture of catalyst 1 and compound 2 after 6 days at room temperature.

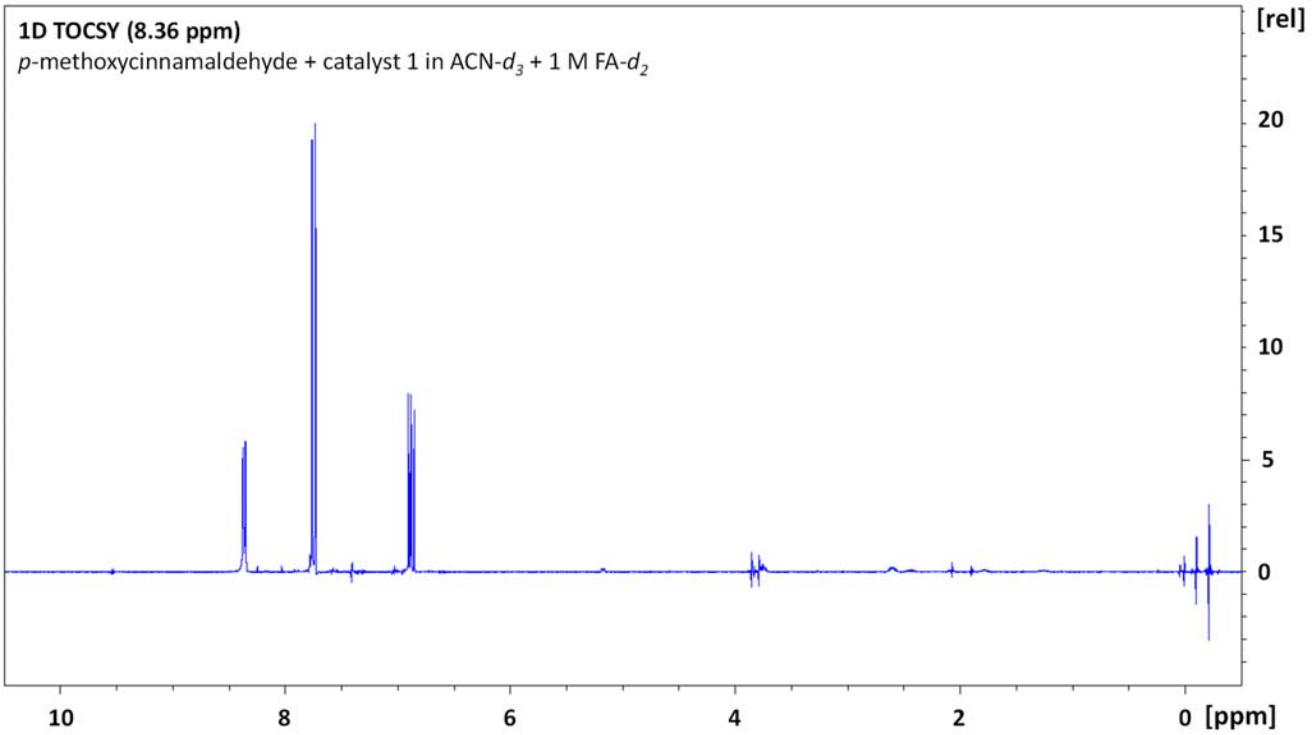

Figure S20 1D selective (8.36 ppm) TOCSY NMR spectrum (500 MHz, CD_3CN/FA) of an equimolar mixture of catalyst 1 and compound 2.

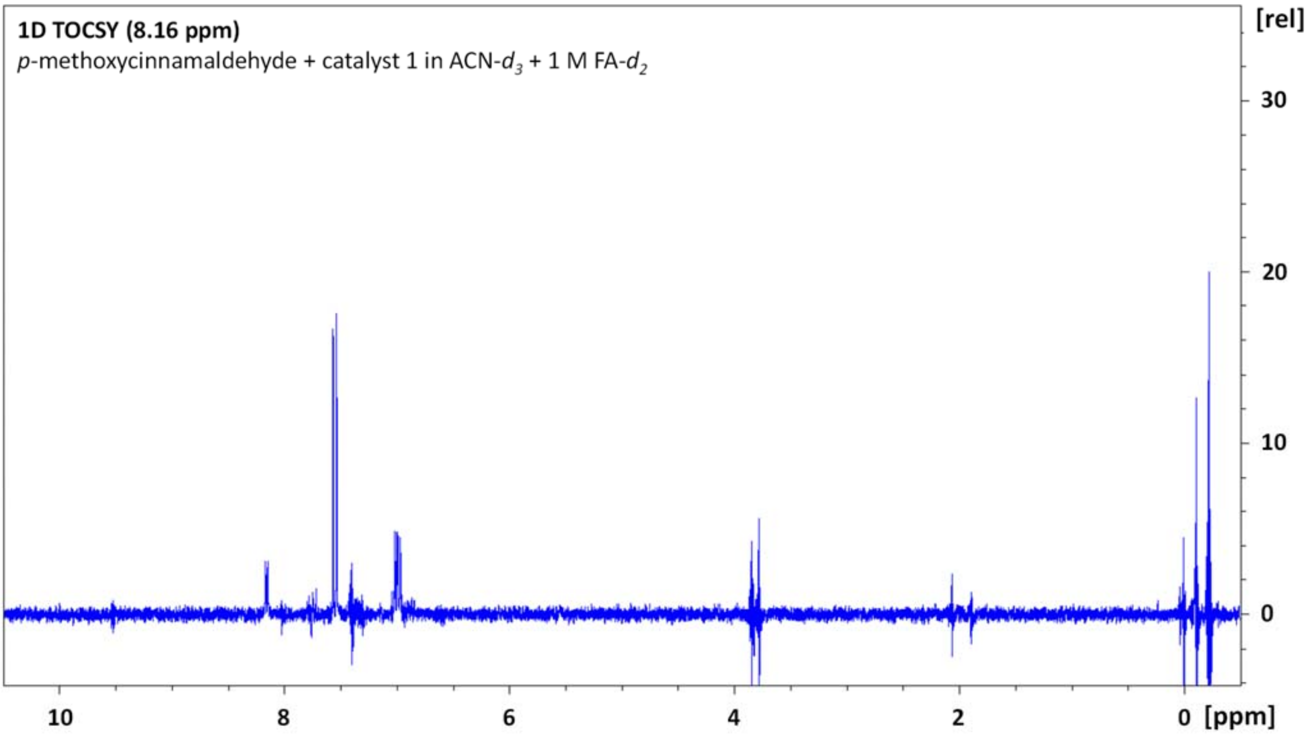

Figure S21 1D selective (8.16 ppm) TOCSY NMR spectrum (500 MHz, CD_3CN/FA) of an equimolar mixture of catalyst 1 and compound 2.

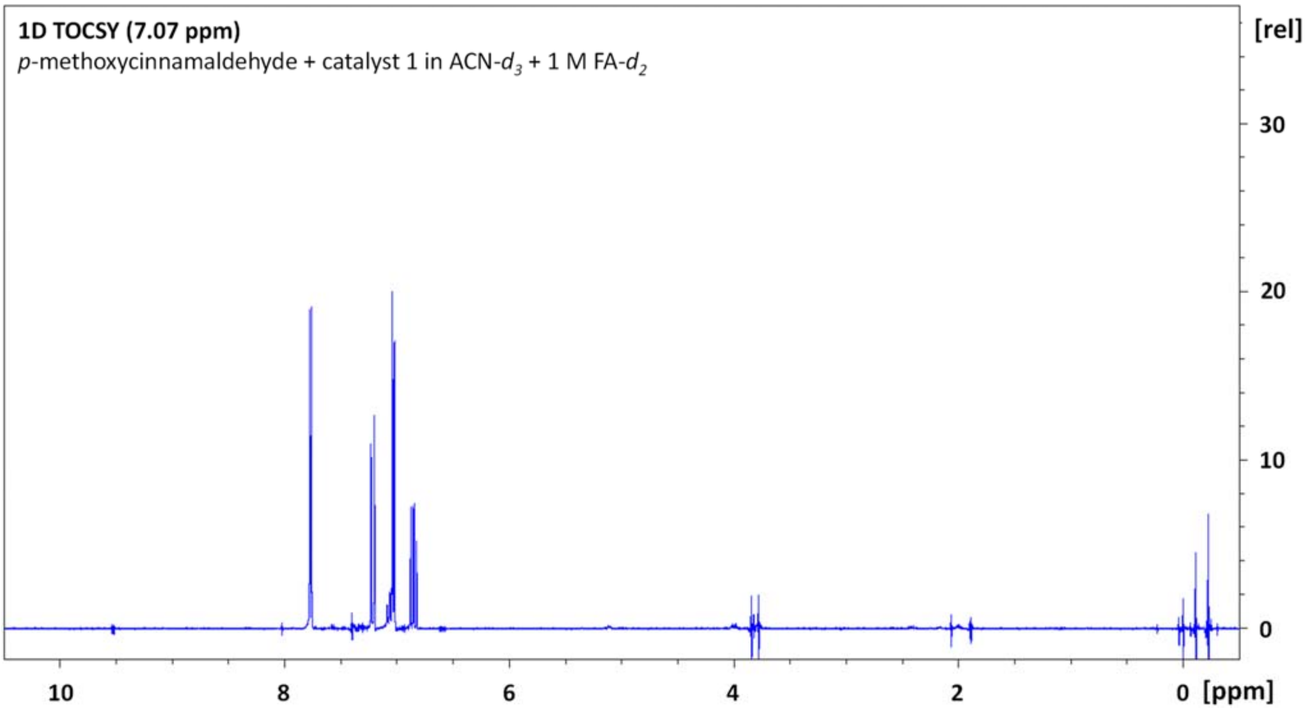

Figure S22 1D selective (7.07 ppm) TOCSY NMR spectrum (500 MHz, CD_3CN/FA) of an equimolar mixture of catalyst 1 and compound 2. Due to slight overlap of peaks, signals at 7.78 and 7.04 ppm are visible. These signals correspond to aryl protons (D and E) of the isomer assigned as 3c (see Fig S3).

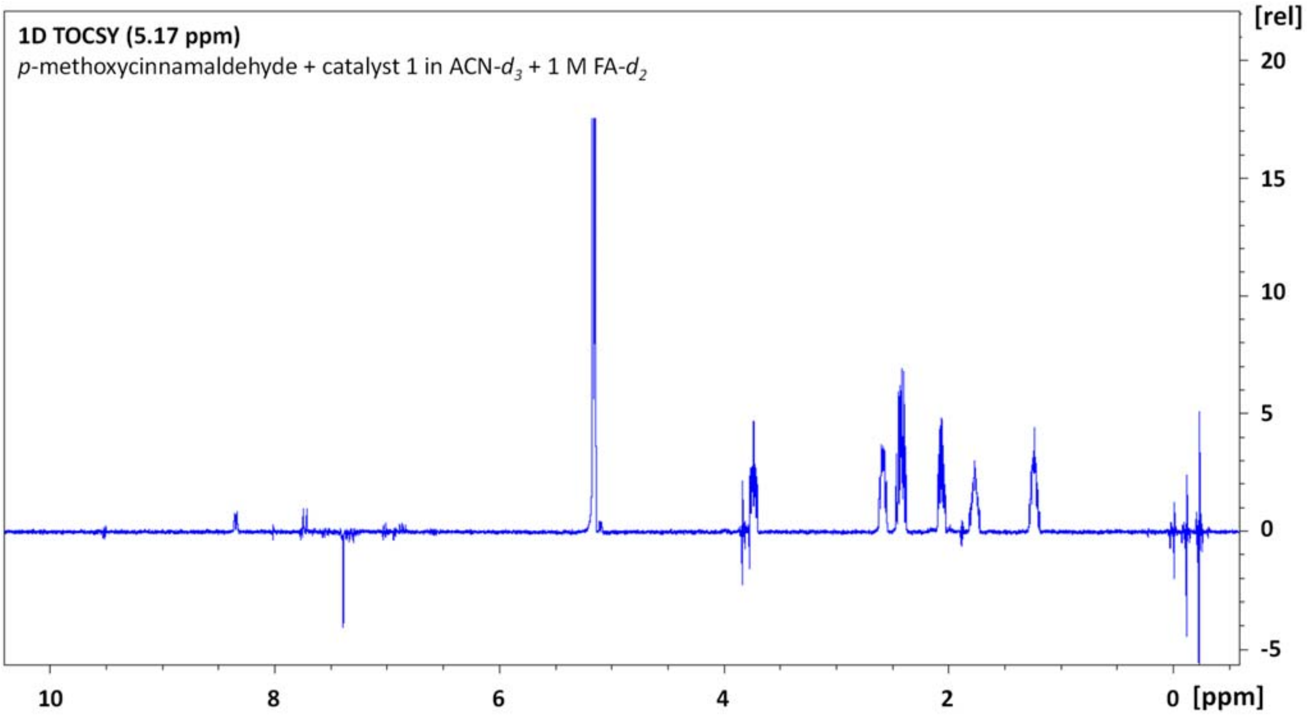

Figure S23 1D selective (5.17 ppm) TOCSY NMR spectrum (500 MHz, CD_3CN/FA) of an equimolar mixture of catalyst 1 and compound 2.

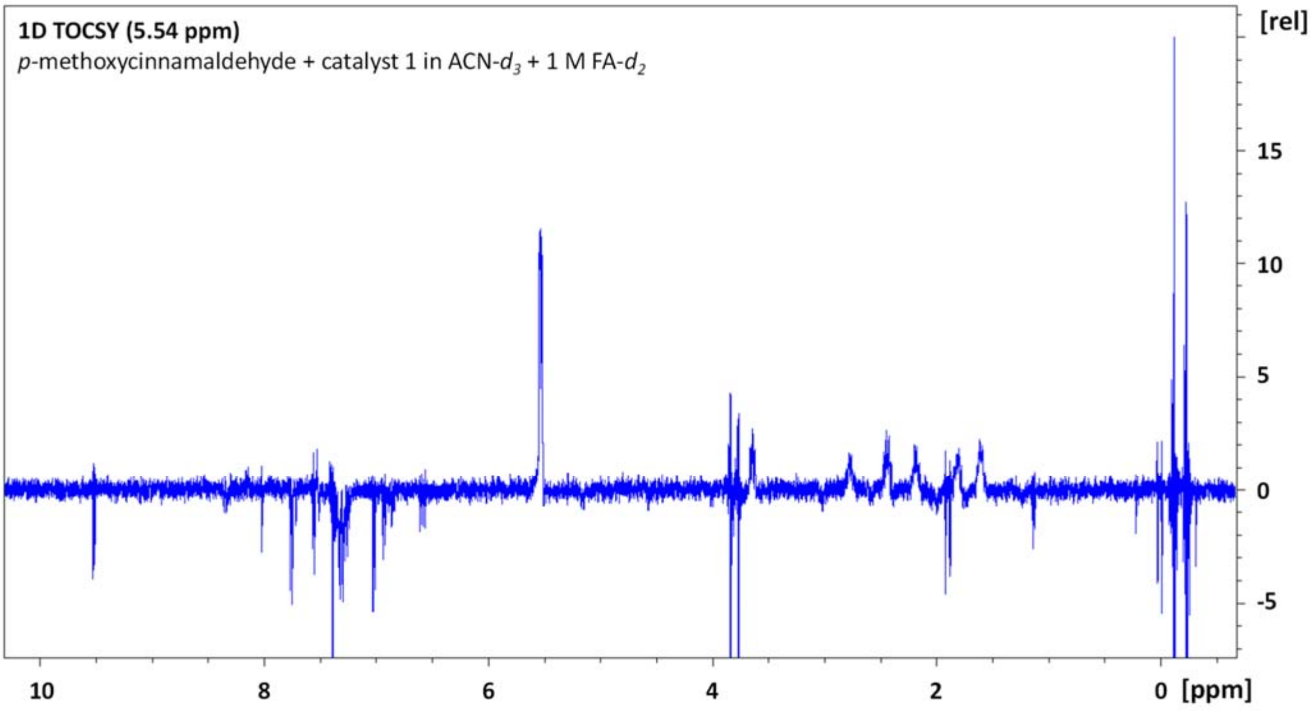

Figure S24 1D selective (5.54 ppm) TOCSY NMR spectrum (500 MHz, CD_3CN/FA) of an equimolar mixture of catalyst 1 and compound 2.

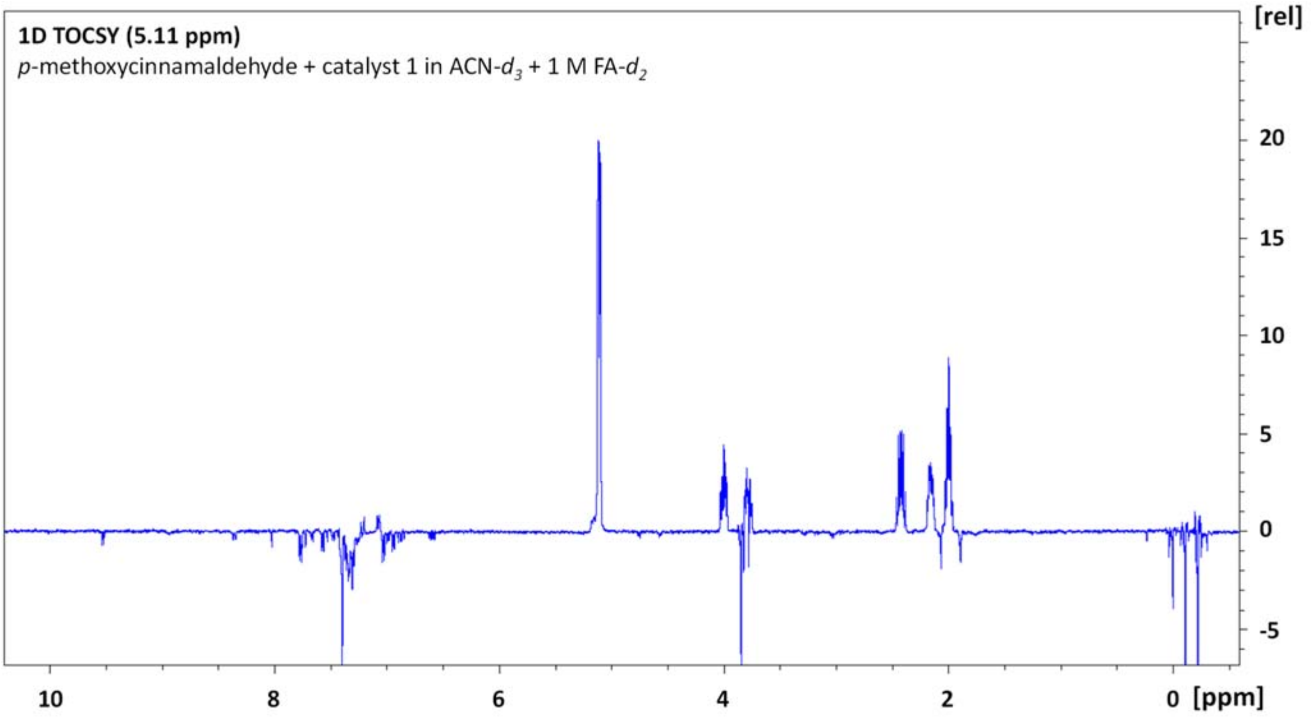

Figure S25 1D selective (5.11 ppm) TOCSY NMR spectrum (500 MHz, CD_3CN/FA) of an equimolar mixture of catalyst 1 and compound 2.

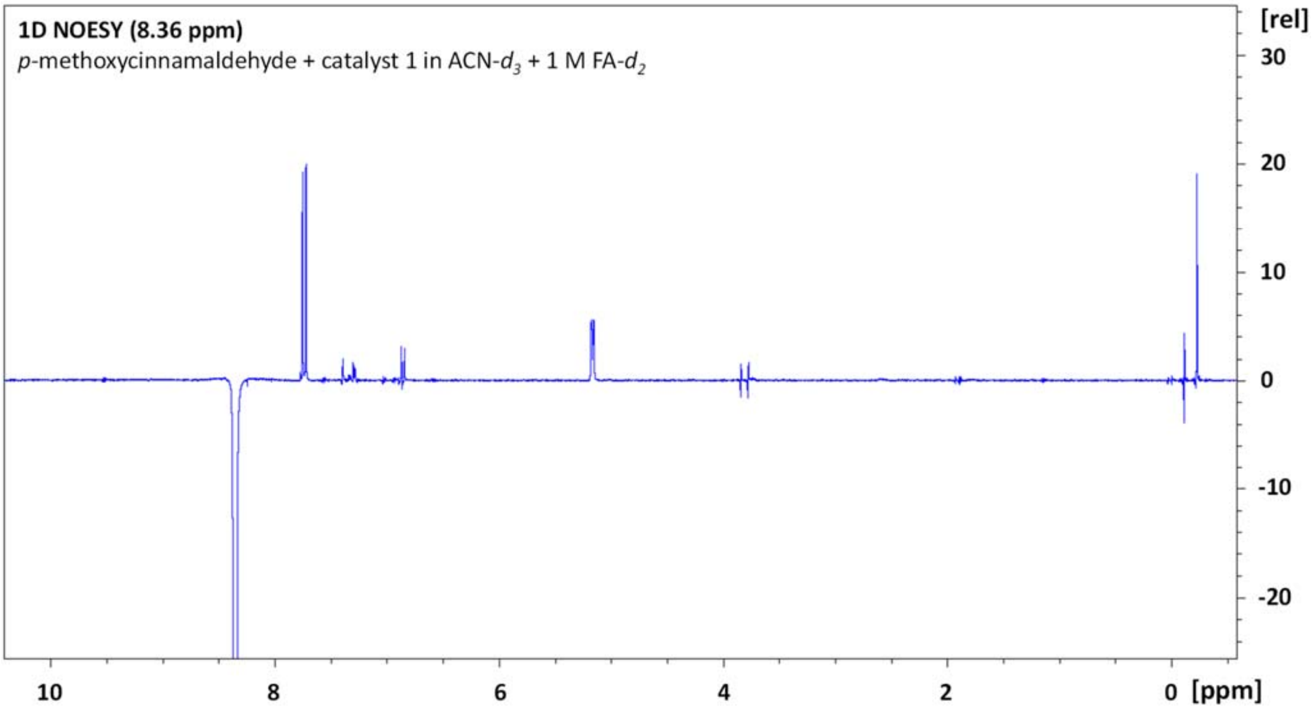

Figure S26 1D selective (8.36 ppm) NOESY NMR spectrum (500 MHz, CD_3CN/FA) of an equimolar mixture of catalyst 1 and compound 2.

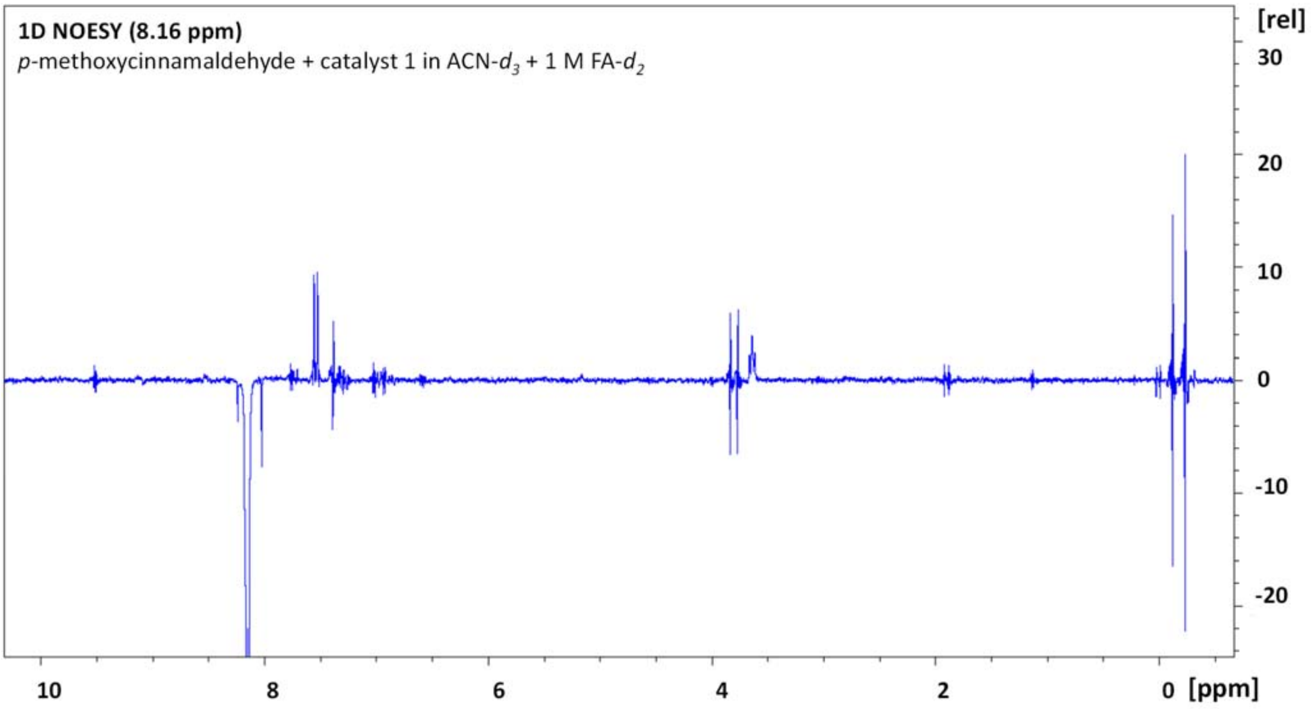

Figure S27 1D selective (8.16 ppm) NOESY NMR spectrum (500 MHz, CD_3CN/FA) of an equimolar mixture of catalyst 1 and compound 2.

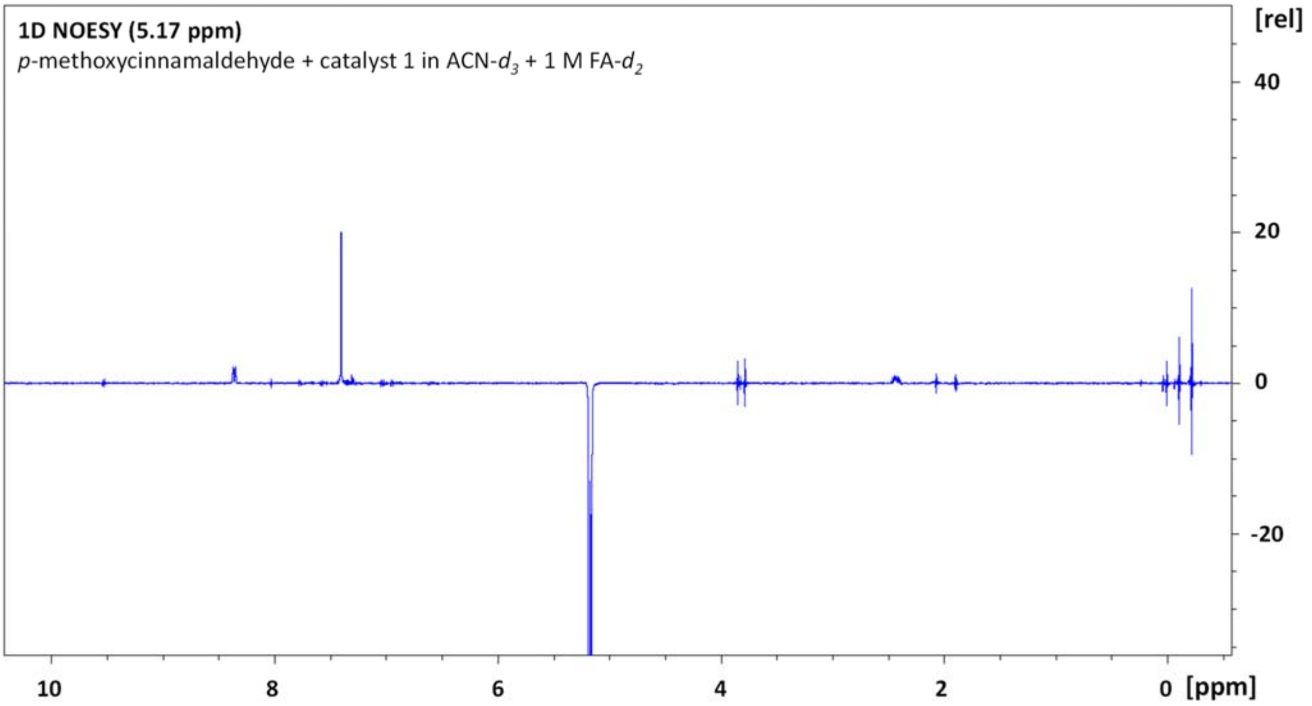

Figure S28 1D selective (5.17 ppm) NOESY NMR spectrum (500 MHz, CD_3CN/FA) of an equimolar mixture of catalyst 1 and compound 2.

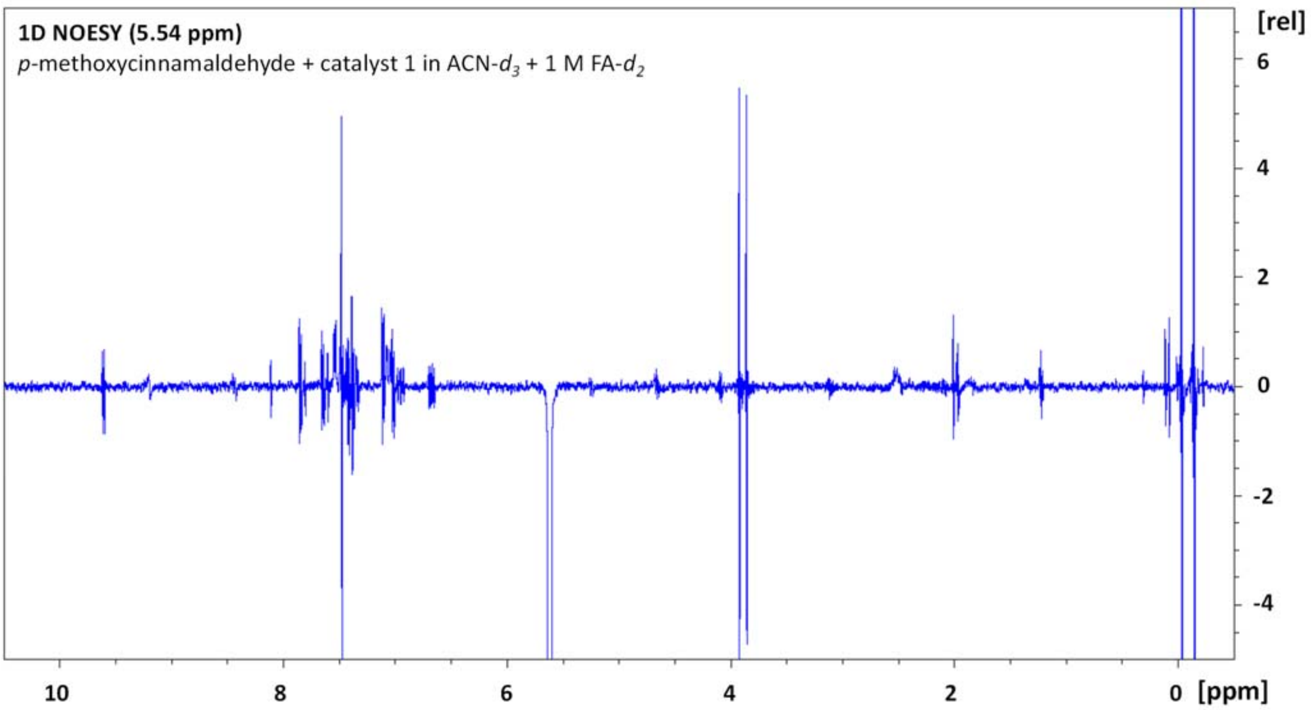

Figure S29 1D selective (5.54 ppm) NOESY NMR spectrum (500 MHz, CD_3CN/FA) of an equimolar mixture of catalyst 1 and compound 2.

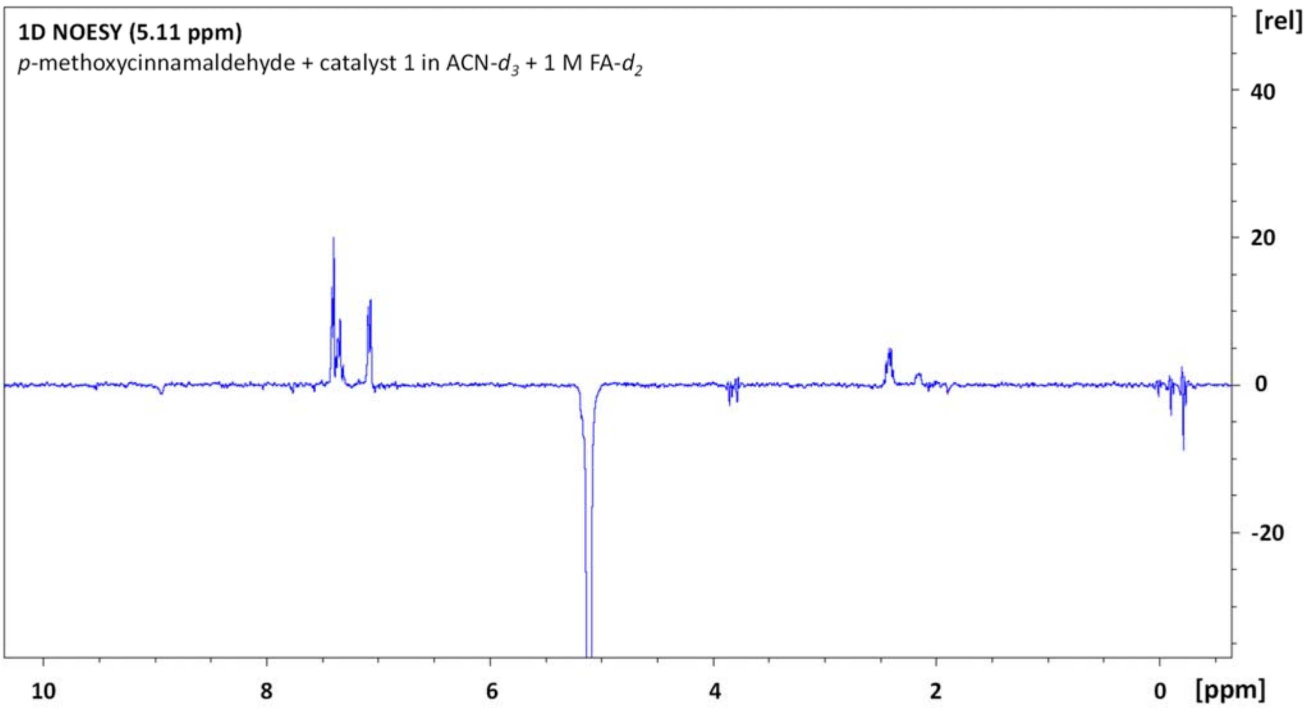

Figure S30 1D selective (5.11 ppm) NOESY NMR spectrum (500 MHz, CD_3CN/FA) of an equimolar mixture of catalyst 1 and compound 2.

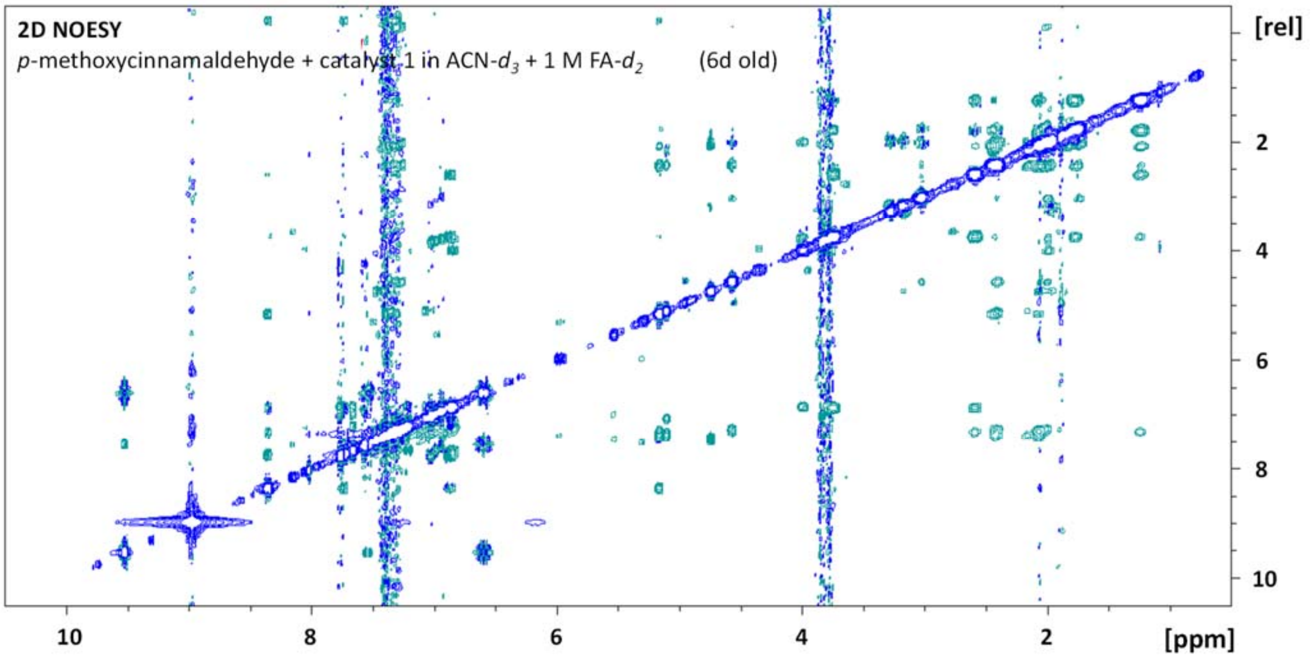

Figure S31 2D NOESY NMR spectrum (400 MHz, CD_3CN/FA) of an equimolar mixture of catalyst 1 and compound 2.

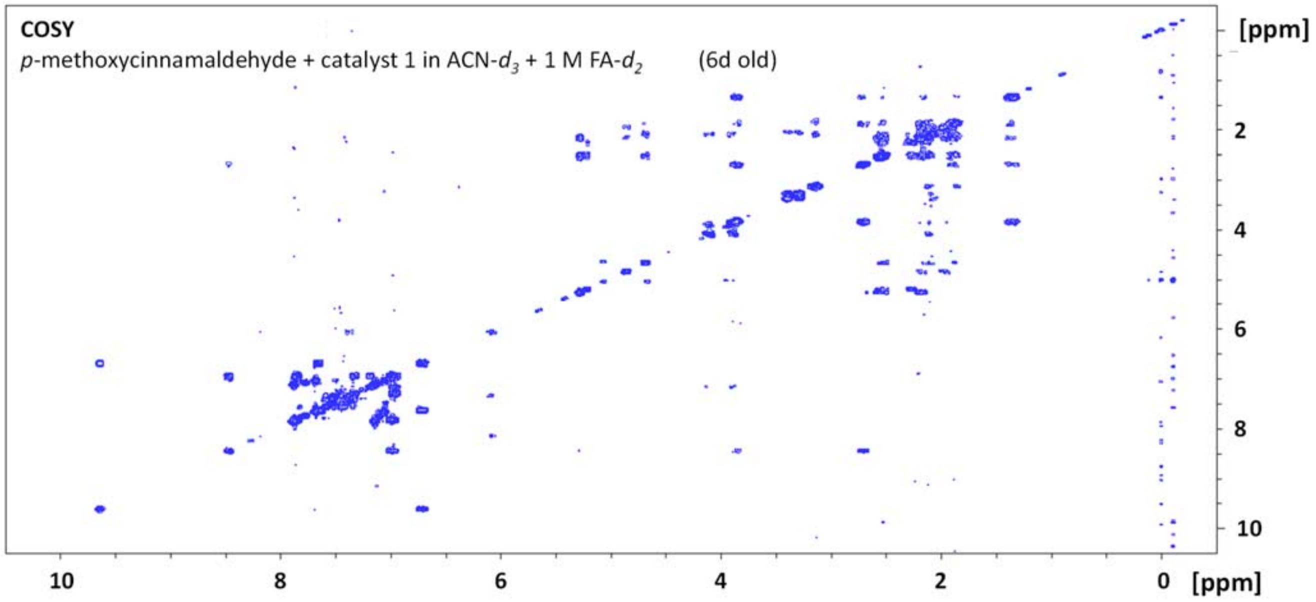

Figure S32 2D COSY NMR spectrum (400 MHz, CD_3CN/FA) of an equimolar mixture of catalyst 1 and compound 2.

Cartesian coordinates of DFT-optimized structures

(B3LYP/6-31G** with GD3 dispersion correction)

Intermediate 3 (*trans* C=N, *trans* C=C, exo)

Si	1.533382000	-2.433017000	0.737707000
O	1.412591000	-0.940205000	-0.062925000
N	0.294309000	1.233416000	-1.405604000
C	1.700000000	0.806159000	-1.600210000
C	2.379671000	2.077211000	-2.131441000
C	1.554576000	3.263808000	-1.587641000
C	0.142373000	2.703922000	-1.346640000
C	-0.700748000	0.386060000	-1.281315000
C	-2.042095000	0.723333000	-1.002541000
C	-2.968358000	-0.283815000	-0.863075000
C	-4.360862000	-0.169117000	-0.556860000
C	-5.021527000	1.074505000	-0.365789000
C	-6.363889000	1.125062000	-0.071995000
C	-7.113664000	-0.069179000	0.046053000
C	-6.482619000	-1.314202000	-0.138565000
C	-5.129999000	-1.349373000	-0.434720000
C	2.281415000	0.169088000	-0.276533000
C	2.720189000	-3.584247000	-0.147971000
C	1.994507000	-2.213209000	2.545139000
C	-0.240804000	-3.046805000	0.575450000
C	2.147161000	1.175990000	0.876343000
C	3.128235000	2.148725000	1.120438000
C	2.944996000	3.116594000	2.108523000
C	1.775555000	3.130716000	2.870772000
C	0.795430000	2.164463000	2.640659000
C	0.980734000	1.196332000	1.652295000
C	3.719735000	-0.331797000	-0.490170000
C	4.190431000	-0.728662000	-1.749491000
C	5.449875000	-1.311314000	-1.895371000
C	6.262494000	-1.518709000	-0.781223000
C	5.807273000	-1.130280000	0.479522000
C	4.551703000	-0.541341000	0.621019000
H	1.689969000	0.004067000	-2.339239000
H	3.427166000	2.125574000	-1.831648000
H	2.353626000	2.061889000	-3.224804000
H	1.972476000	3.625804000	-0.648988000
H	1.534881000	4.098553000	-2.290865000
H	-0.571256000	3.005369000	-2.120139000
H	-0.256614000	2.985186000	-0.368066000
H	-0.430267000	-0.658670000	-1.380089000
H	-2.320167000	1.763014000	-0.874545000
H	-2.601920000	-1.303151000	-0.987250000
H	-4.469476000	2.004359000	-0.452545000
H	-6.879822000	2.067208000	0.074766000
H	-7.038581000	-2.238870000	-0.052960000

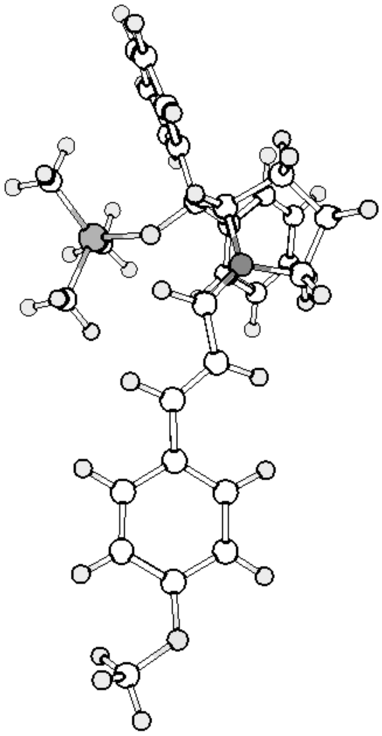

H	-4.646995000	-2.312379000	-0.576236000
H	2.663386000	-4.588758000	0.286864000
H	3.755263000	-3.241178000	-0.070948000
H	2.471717000	-3.667104000	-1.210976000
H	1.802621000	-3.142722000	3.093169000
H	1.409113000	-1.417454000	3.015560000
H	3.053354000	-1.972276000	2.670502000
H	-0.371540000	-4.017682000	1.064449000
H	-0.517250000	-3.171206000	-0.478158000
H	-0.944110000	-2.344106000	1.035984000
H	4.046353000	2.146894000	0.543005000
H	3.720435000	3.855534000	2.285182000
H	1.635194000	3.880797000	3.642665000
H	-0.112977000	2.157555000	3.235692000
H	0.218449000	0.445957000	1.481265000
H	3.584214000	-0.599840000	-2.638750000
H	5.791382000	-1.607668000	-2.882207000
H	7.240280000	-1.975971000	-0.894286000
H	6.428864000	-1.283544000	1.356204000
H	4.215088000	-0.243351000	1.606835000
O	-8.410721000	0.096661000	0.334769000
C	-9.257511000	-1.051394000	0.473556000
H	-8.917087000	-1.693345000	1.293218000
H	-10.246728000	-0.658249000	0.703523000
H	-9.297169000	-1.624714000	-0.458921000

Intermediate 3 (*cis* C=N, *trans* C=C exo)

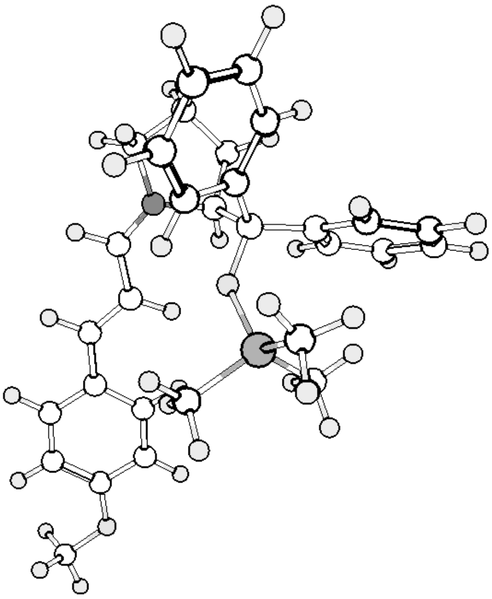

Si	-0.170739000	1.510733000	1.498489000
O	-0.681102000	0.308774000	0.403212000
N	-0.949575000	-2.125427000	-1.087123000
C	-1.495543000	-0.814191000	-1.502698000
C	-2.610554000	-1.207022000	-2.490043000
C	-3.058954000	-2.642331000	-2.119440000
C	-1.970473000	-3.200039000	-1.181579000
C	0.302062000	-2.381538000	-0.779367000
C	1.378029000	-1.472976000	-0.729239000
C	2.642665000	-1.929213000	-0.448667000
C	3.844672000	-1.155427000	-0.367537000
C	3.891557000	0.241487000	-0.626989000
C	5.069847000	0.942926000	-0.520464000
C	6.263480000	0.279727000	-0.151011000
C	6.244961000	-1.104525000	0.104702000
C	5.050693000	-1.797963000	-0.006705000
C	-1.916299000	0.074784000	-0.267684000
C	0.234235000	3.088981000	0.563760000
C	-1.422451000	1.832610000	2.863253000
C	1.378596000	0.738984000	2.230637000
C	-2.862931000	-0.732048000	0.635382000
C	-4.233834000	-0.841016000	0.359267000
C	-5.056268000	-1.661176000	1.132758000
C	-4.522500000	-2.387597000	2.198426000

C	-3.160757000	-2.282133000	2.486472000
C	-2.339270000	-1.463211000	1.710129000
C	-2.502494000	1.413841000	-0.753538000
C	-2.035474000	2.029847000	-1.924778000
C	-2.466727000	3.304433000	-2.290172000
C	-3.368664000	3.999211000	-1.483641000
C	-3.839012000	3.402598000	-0.314023000
C	-3.412890000	2.123236000	0.043573000
H	-0.689707000	-0.279125000	-2.003909000
H	-3.432101000	-0.490995000	-2.462889000
H	-2.202209000	-1.186845000	-3.503725000
H	-4.019315000	-2.635260000	-1.605014000
H	-3.163018000	-3.261792000	-3.012348000
H	-1.492639000	-4.104787000	-1.564803000
H	-2.358736000	-3.400631000	-0.180405000
H	0.510094000	-3.430333000	-0.571718000
H	1.178066000	-0.421519000	-0.874049000
H	2.759615000	-2.995535000	-0.252663000
H	2.991216000	0.772034000	-0.917881000
H	5.117578000	2.008215000	-0.715936000
H	7.147643000	-1.631176000	0.386209000
H	5.042046000	-2.865826000	0.193933000
H	0.711688000	3.814369000	1.232206000
H	-0.665025000	3.552481000	0.148677000
H	0.924214000	2.897259000	-0.265482000
H	-0.917527000	2.326198000	3.701687000
H	-1.862816000	0.904443000	3.238808000
H	-2.233207000	2.487700000	2.536581000
H	1.831397000	1.415208000	2.963851000
H	2.134634000	0.521122000	1.471380000
H	1.143396000	-0.198275000	2.745985000
H	-4.668492000	-0.272729000	-0.455888000
H	-6.115905000	-1.724871000	0.905407000
H	-5.163116000	-3.021374000	2.803398000
H	-2.735774000	-2.835079000	3.318775000
H	-1.280980000	-1.391616000	1.929845000
H	-1.317803000	1.532778000	-2.567605000
H	-2.091306000	3.756009000	-3.203154000
H	-3.701593000	4.993216000	-1.764814000
H	-4.541090000	3.930040000	0.324204000
H	-3.789926000	1.677919000	0.955940000
O	7.350991000	1.058417000	-0.076025000
C	8.610940000	0.480190000	0.287380000
H	8.917985000	-0.280239000	-0.438796000
H	9.323667000	1.303601000	0.277931000
H	8.567471000	0.042934000	1.290793000

Intermediate 3 (*trans* C=N, *cis* C=C, exo)

H	6.210907000	-0.161651000	1.448827000
C	4.599784000	-1.415874000	0.812293000
H	4.683380000	-2.059321000	1.683589000

C	3.584764000	-1.669677000	-0.137596000
C	3.534223000	-0.837213000	-1.286054000
H	2.846165000	-1.072327000	-2.090849000
C	4.403437000	0.222785000	-1.435703000
H	4.390326000	0.847172000	-2.321955000
C	5.363886000	0.500209000	-0.439529000
C	5.463463000	-0.339713000	0.686484000
O	6.147811000	1.562959000	-0.674995000
C	7.178431000	1.899703000	0.261215000
H	7.666620000	2.783713000	-0.146733000
H	6.755727000	2.134415000	1.244385000
H	7.907587000	1.087359000	0.352957000
C	2.667163000	-2.752691000	0.107800000
C	1.364064000	-2.894443000	-0.305841000
H	3.055852000	-3.557937000	0.730100000
H	0.854980000	-3.826996000	-0.087550000
C	0.607599000	-1.816345000	-0.831476000
H	1.015621000	-0.813914000	-0.793400000
N	-0.624323000	-1.886505000	-1.267995000
C	-1.440758000	-0.681167000	-1.575395000
H	-0.818784000	-0.029769000	-2.191939000
C	-2.652210000	-1.236330000	-2.353360000
H	-3.554068000	-1.164252000	-1.746998000
H	-2.830704000	-0.661002000	-3.262684000
C	-1.402986000	-3.119044000	-1.492441000
H	-1.962134000	-3.344923000	-0.577406000
H	-0.736748000	-3.950016000	-1.727388000
H	-3.214589000	-3.342273000	-2.697118000
H	-1.786036000	-2.812404000	-3.594750000
C	-2.323411000	-2.713892000	-2.646607000
C	-1.756493000	0.124999000	-0.251133000
C	-3.686224000	-1.214104000	0.802432000
H	-4.388559000	-0.706715000	0.150477000
C	-4.159964000	-2.197820000	1.671830000
H	-5.214773000	-2.454275000	1.665345000
C	-3.287086000	-2.839085000	2.551238000
H	-3.655789000	-3.601747000	3.229828000
C	-1.939053000	-2.477871000	2.560923000
H	-1.251600000	-2.957413000	3.251375000
C	-1.467495000	-1.494700000	1.690587000
H	-0.419696000	-1.222689000	1.705836000
C	-2.329808000	-0.851739000	0.791516000
C	-2.584996000	2.002804000	-1.782573000
H	-1.941413000	1.640825000	-2.576463000
C	-3.311253000	3.171801000	-2.006594000
H	-3.233477000	3.676305000	-2.964573000
C	-4.125021000	3.696548000	-1.002113000
H	-4.687022000	4.608965000	-1.174122000
C	-4.208898000	3.038184000	0.224975000
H	-4.836431000	3.435741000	1.016509000
C	-3.488105000	1.864083000	0.442922000
H	-3.565457000	1.366124000	1.401974000

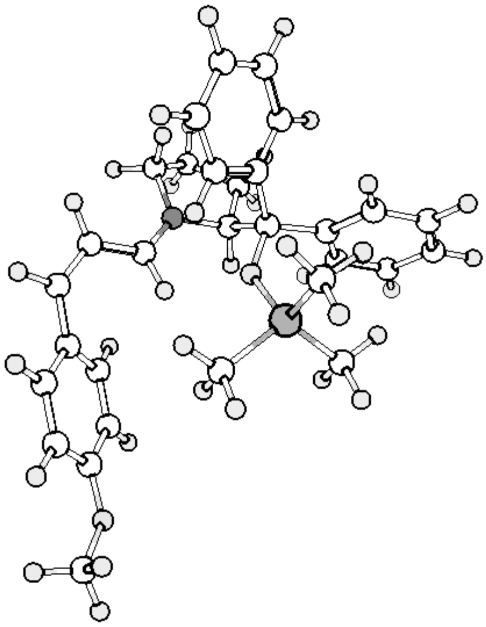

C	-2.665073000	1.325403000	-0.557264000
Si	0.067469000	1.869003000	1.136756000
C	-0.771718000	1.854401000	2.817400000
C	-0.172774000	3.514916000	0.268642000
C	1.895195000	1.458591000	1.304797000
H	-0.255785000	2.548007000	3.491258000
H	-0.748017000	0.860930000	3.274665000
H	-1.815412000	2.173156000	2.756682000
H	0.259024000	3.499473000	-0.737262000
H	0.325761000	4.311245000	0.833207000
H	-1.229899000	3.777825000	0.176564000
H	2.045517000	0.479141000	1.771211000
H	2.402849000	2.203690000	1.926736000
H	2.394302000	1.443456000	0.330699000
O	-0.469637000	0.582699000	0.157756000

Intermediate 3 (*cis* C=N, *cis* C=C, exo)

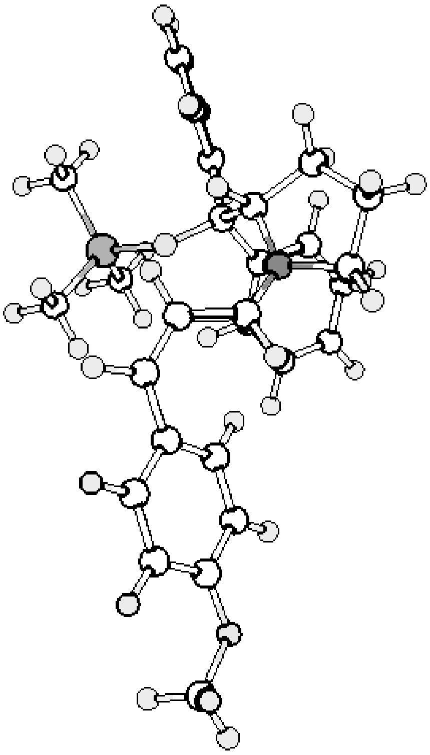

C	-3.580243000	0.120645000	0.096659000
C	-3.562053000	-0.892601000	-0.898973000
C	-4.792899000	-1.500608000	-1.235797000
C	-5.991656000	-1.078133000	-0.684345000
C	-5.988751000	-0.041002000	0.268844000
C	-4.765415000	0.539945000	0.664152000
C	-2.360821000	-1.354363000	-1.547279000
C	-1.169140000	-0.697831000	-1.728011000
C	-1.027219000	0.699478000	-1.568717000
N	0.083756000	1.395625000	-1.562592000
C	1.480914000	0.881510000	-1.590487000
C	2.312347000	2.111399000	-2.033738000
C	1.295697000	3.189453000	-2.452194000
C	0.083848000	2.876099000	-1.578106000
C	1.906585000	0.231812000	-0.205485000
O	1.229611000	-1.019528000	-0.192018000
Si	1.505869000	-2.456505000	0.686768000
C	1.647329000	-2.122857000	2.530249000
O	-7.081444000	0.442453000	0.879663000
C	-8.364966000	-0.114506000	0.572403000
C	1.383760000	1.135603000	0.925320000
C	2.053517000	2.302687000	1.324173000
C	1.487023000	3.171165000	2.260008000
C	0.238554000	2.891057000	2.815956000
C	-0.433346000	1.728080000	2.435172000
C	0.133923000	0.863093000	1.499589000
C	3.424649000	-0.012558000	-0.186324000
C	4.083142000	-0.449173000	-1.346285000
C	5.430409000	-0.802684000	-1.318969000
C	6.147148000	-0.742945000	-0.122527000
C	5.502582000	-0.326185000	1.041563000
C	4.155000000	0.036698000	1.008950000
C	-0.053866000	-3.436573000	0.308364000
C	3.020614000	-3.366017000	0.055114000

H	-4.785979000	1.295460000	1.441640000
H	-6.915952000	-1.554990000	-0.984359000
H	-4.803890000	-2.302101000	-1.969036000
H	-2.647647000	0.531504000	0.468860000
H	-9.076874000	0.434956000	1.186862000
H	-8.611181000	0.023403000	-0.486201000
H	-8.403893000	-1.178913000	0.828685000
H	-2.399181000	-2.376395000	-1.920787000
H	-0.302297000	-1.269458000	-2.032941000
H	-1.932536000	1.297834000	-1.509506000
H	0.223300000	3.228683000	-0.550838000
H	1.667446000	4.203427000	-2.292385000
H	2.999439000	1.848467000	-2.838526000
H	3.545499000	-0.542323000	-2.283645000
H	5.916983000	-1.135856000	-2.230288000
H	7.195494000	-1.022738000	-0.098367000
H	6.045392000	-0.282321000	1.980592000
H	3.671865000	0.351865000	1.925835000
H	-0.391419000	-0.037544000	1.207162000
H	-1.397867000	1.487194000	2.873063000
H	-0.198718000	3.563853000	3.546795000
H	2.029793000	4.062515000	2.558965000
H	3.035770000	2.529978000	0.925650000
H	-0.952610000	-2.901681000	0.631592000
H	-0.145287000	-3.637834000	-0.764382000
H	-0.034488000	-4.402687000	0.823669000
H	3.053301000	-4.370730000	0.492286000
H	2.995276000	-3.477914000	-1.033434000
H	3.949196000	-2.853121000	0.318410000
H	2.551103000	-1.558785000	2.774703000
H	0.788332000	-1.566713000	2.915779000
H	1.700638000	-3.076277000	3.068363000
H	1.514385000	0.080936000	-2.331678000
H	1.027270000	3.087356000	-3.508196000
H	-0.866530000	3.247887000	-1.964262000
H	2.922073000	2.467234000	-1.203520000

Intermediate 4 (*R*)

C	2.516192000	-1.255871000	-0.098557000
C	3.457772000	-0.272017000	0.246101000
C	4.808422000	-0.612547000	0.165946000
C	5.224239000	-1.879249000	-0.262271000
C	4.270319000	-2.840085000	-0.608159000
C	2.908018000	-2.519838000	-0.514961000
C	2.972109000	1.101311000	0.753499000
C	1.813014000	1.615376000	-0.074583000
C	0.549733000	1.652274000	0.390469000
N	-0.564096000	2.084053000	-0.291580000
C	-1.923567000	1.897930000	0.181605000
C	-2.727552000	2.907389000	-0.661365000
C	-1.918036000	3.088387000	-1.960067000

C	-0.461185000	2.816224000	-1.548333000
C	-2.475684000	0.408412000	0.077022000
O	-3.818929000	0.532259000	0.547702000
Si	-4.932561000	-0.579009000	1.154572000
C	-4.911270000	-0.556760000	3.036946000
O	4.554296000	-4.107868000	-1.040112000
C	5.916867000	-4.484271000	-1.147309000
C	-2.441347000	-0.022968000	-1.391776000
C	-1.218343000	-0.257757000	-2.044548000
C	-1.178748000	-0.543694000	-3.408217000
C	-2.358892000	-0.598653000	-4.152902000
C	-3.577245000	-0.363037000	-3.517264000
C	-3.615190000	-0.074541000	-2.151333000
C	-1.700186000	-0.531879000	1.023823000
C	-1.580269000	-0.175344000	2.377950000
C	-0.909488000	-0.990507000	3.287094000
C	-0.350221000	-2.198964000	2.866317000
C	-0.492845000	-2.585951000	1.535779000
C	-1.164627000	-1.763714000	0.627400000
C	-6.591398000	0.073588000	0.548641000
C	-4.633322000	-2.324240000	0.515612000
H	2.177596000	-3.278945000	-0.777065000
H	6.284290000	-2.097419000	-0.315652000
H	5.561699000	0.114535000	0.453041000
H	1.459384000	-1.025631000	-0.025068000
H	5.919796000	-5.515943000	-1.503416000
H	6.458653000	-3.854244000	-1.865828000
H	6.430605000	-4.437109000	-0.177464000
H	2.605667000	0.942846000	1.778360000
H	2.036243000	1.904858000	-1.097438000
H	0.344592000	1.306459000	1.399523000
H	0.067604000	2.228666000	-2.310597000
H	-2.235984000	2.362987000	-2.711753000
H	-2.780204000	3.852949000	-0.111959000
H	-2.030713000	0.745558000	2.731200000
H	-0.826293000	-0.681703000	4.325089000
H	0.181914000	-2.832671000	3.569094000
H	-0.076230000	-3.528767000	1.194347000
H	-1.272716000	-2.096194000	-0.397058000
H	-4.562679000	0.141625000	-1.675000000
H	-4.503683000	-0.395053000	-4.083610000
H	-2.327355000	-0.820847000	-5.215472000
H	-0.220382000	-0.721039000	-3.887693000
H	-0.288768000	-0.200329000	-1.490525000
H	-7.420195000	-0.488968000	0.992935000
H	-6.716039000	1.126582000	0.821907000
H	-6.684093000	-0.001934000	-0.539851000
H	-5.484686000	-2.965107000	0.773295000
H	-4.518037000	-2.335458000	-0.572432000
H	-3.733355000	-2.763850000	0.953565000
H	-3.991093000	-0.993720000	3.433897000
H	-4.991643000	0.467694000	3.416281000

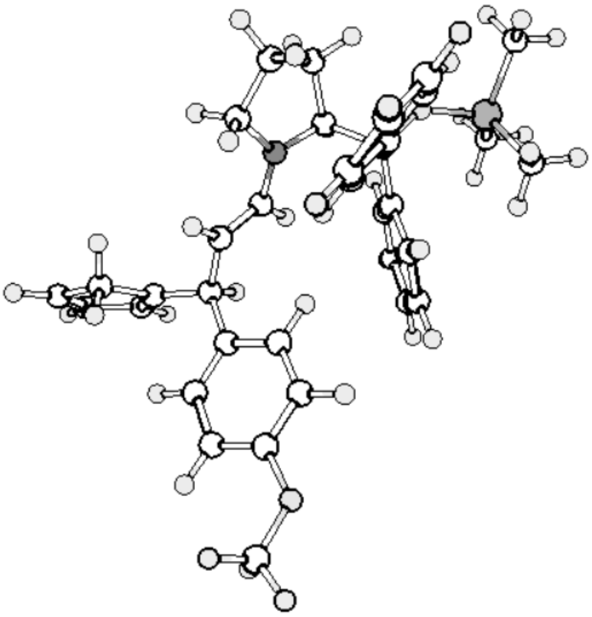

H	-5.758428000	-1.127314000	3.435145000
H	-1.997626000	2.158600000	1.242686000
H	-2.040041000	4.086867000	-2.388455000
H	0.119489000	3.737489000	-1.387518000
H	-3.748965000	2.566149000	-0.833641000
C	4.082034000	2.118967000	0.835913000
C	4.696420000	2.800761000	-0.367226000
C	5.766141000	3.678736000	0.226584000
C	5.754994000	3.533296000	1.567264000
C	4.714862000	2.567519000	1.942586000
H	5.094996000	2.073556000	-1.088299000
H	3.946967000	3.390760000	-0.913250000
H	6.412487000	4.324803000	-0.354898000
H	6.402799000	4.041536000	2.273596000
H	4.495712000	2.260675000	2.960232000
C	2.516192000	-1.255871000	-0.098557000

Intermediate 4 (*S*)

C	4.640799000	0.068431000	-1.765479000
C	4.137613000	0.178414000	-0.459433000
C	5.009030000	-0.079411000	0.598732000
C	6.345427000	-0.432662000	0.379633000
C	6.827695000	-0.531587000	-0.928576000
C	5.963122000	-0.277774000	-2.004056000
C	2.660129000	0.512620000	-0.238845000
C	1.782863000	-0.715903000	-0.379538000
C	0.598291000	-0.633080000	-1.015099000
N	-0.371474000	-1.600064000	-1.131796000
C	-1.743629000	-1.299219000	-1.507970000
C	-2.361475000	-2.696199000	-1.704316000
C	-1.544157000	-3.635011000	-0.794829000
C	-0.155621000	-2.977646000	-0.704147000
C	-2.551662000	-0.385151000	-0.476268000
O	-3.846609000	-0.297083000	-1.074941000
Si	-5.129430000	0.774248000	-0.837660000
C	-5.165094000	2.056289000	-2.216904000
O	8.111773000	-0.862256000	-1.267509000
C	9.028114000	-1.133370000	-0.220201000
C	-2.615944000	-1.106748000	0.873611000
C	-3.796077000	-1.730037000	1.295401000
C	-3.836466000	-2.479804000	2.472724000
C	-2.689612000	-2.630517000	3.251109000
C	-1.501924000	-2.025556000	2.835795000
C	-1.463683000	-1.276348000	1.660941000
C	-1.925421000	1.025565000	-0.427890000
C	-1.430096000	1.636886000	0.728794000
C	-0.821902000	2.894050000	0.679473000
C	-0.717106000	3.579157000	-0.528842000
C	-1.245577000	3.003424000	-1.687618000
C	-1.841813000	1.746183000	-1.633322000
C	-6.668503000	-0.302525000	-0.979679000

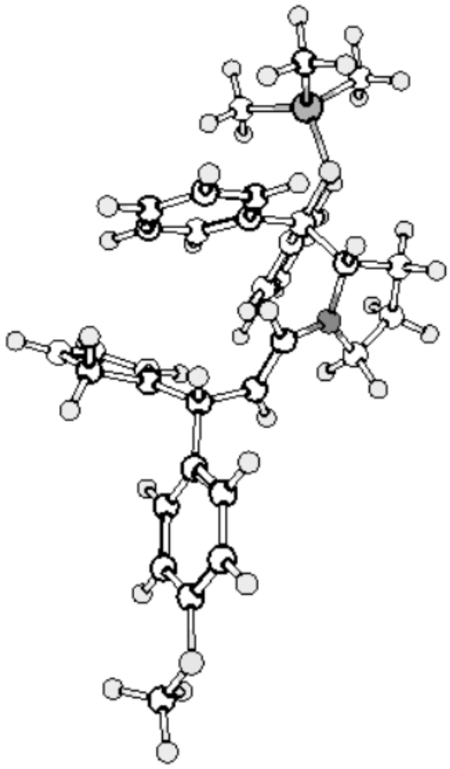

C	-5.052272000	1.619436000	0.841924000
H	6.989691000	-0.622096000	1.229969000
H	6.355463000	-0.358498000	-3.012581000
H	3.976848000	0.251770000	-2.606811000
H	4.643012000	0.001317000	1.618184000
H	9.977124000	-1.374078000	-0.702333000
H	9.168152000	-0.262631000	0.434747000
H	8.707879000	-1.988400000	0.390566000
H	2.375966000	1.210592000	-1.040246000
H	2.106654000	-1.623033000	0.121753000
H	0.327304000	0.309216000	-1.480310000
H	0.242114000	-3.018605000	0.319002000
H	-1.995979000	-3.690320000	0.197749000
H	-2.242216000	-2.984439000	-2.753810000
H	-0.524760000	-0.837698000	1.346919000
H	-0.595805000	-2.139881000	3.424433000
H	-2.718426000	-3.215113000	4.165902000
H	-4.766853000	-2.952857000	2.773883000
H	-4.683244000	-1.653360000	0.681180000
H	-2.254698000	1.316692000	-2.539319000
H	-1.190911000	3.531529000	-2.635310000
H	-0.239316000	4.553770000	-0.568721000
H	-0.410639000	3.318442000	1.590191000
H	-1.502752000	1.136874000	1.684784000
H	-5.078324000	1.577468000	-3.198333000
H	-6.112427000	2.607624000	-2.198126000
H	-4.350319000	2.778370000	-2.118419000
H	-7.574000000	0.314406000	-0.998634000
H	-6.644480000	-0.889749000	-1.903695000
H	-6.761086000	-1.001353000	-0.142007000
H	-4.956234000	0.889504000	1.651157000
H	-4.201863000	2.303652000	0.904914000
H	-5.968296000	2.197350000	1.011468000
H	-1.765120000	-0.742352000	-2.450911000
H	-1.489282000	-4.651931000	-1.192648000
H	0.587193000	-3.454392000	-1.360218000
H	-3.428536000	-2.698728000	-1.478413000
C	2.420907000	1.249135000	1.060664000
C	2.792521000	2.701776000	1.248059000
C	2.362126000	3.005369000	2.658778000
C	1.826518000	1.889359000	3.196719000
C	1.861929000	0.804261000	2.208802000
H	3.866199000	2.870887000	1.086407000
H	2.265760000	3.330664000	0.515105000
H	2.477364000	3.970507000	3.137089000
H	1.424472000	1.789845000	4.199580000
H	1.481077000	-0.196107000	2.376389000
C	4.640799000	0.068431000	-1.765479000

Intermediate 5 (*R*) (=C$_\beta$-protonated intermediate 4)

C	2.799304000	1.164550000	1.182122000

C	2.798604000	0.420453000	-0.009102000
C	2.518762000	1.101425000	-1.196227000
C	2.210201000	2.462173000	-1.213220000
C	2.182428000	3.178322000	-0.010751000
C	2.491126000	2.519506000	1.189340000
C	3.148526000	-1.061188000	-0.051379000
C	2.413063000	-1.876279000	1.088921000
C	0.945849000	-1.839738000	0.893337000
N	0.076331000	-1.185306000	1.590607000
C	-1.417970000	-1.227661000	1.323741000
C	-2.029350000	-0.561991000	2.569514000
C	-0.949658000	-0.673336000	3.654387000
C	0.356694000	-0.496470000	2.882305000
C	-1.961183000	-0.703822000	-0.072389000
O	-3.300693000	-1.158704000	-0.021474000
Si	-4.874528000	-0.598520000	0.291321000
C	-5.638377000	-1.998416000	1.283072000
O	1.857459000	4.492205000	0.105889000
C	1.580590000	5.230545000	-1.083762000
C	-1.883204000	0.802994000	-0.337095000
C	-1.108703000	1.708296000	0.389429000
C	-1.124779000	3.073563000	0.085187000
C	-1.899681000	3.546026000	-0.970844000
C	-2.635076000	2.641653000	-1.743581000
C	-2.618018000	1.286167000	-1.432897000
C	-1.252494000	-1.475283000	-1.198102000
C	-1.586571000	-2.818405000	-1.424459000
C	-0.870953000	-3.579480000	-2.349083000
C	0.183808000	-3.006623000	-3.066474000
C	0.505732000	-1.664733000	-2.861697000
C	-0.208235000	-0.901416000	-1.933522000
C	-4.922952000	1.009116000	1.268466000
C	-5.770276000	-0.397861000	-1.347106000
H	2.495129000	3.095576000	2.108708000
H	1.986069000	2.944315000	-2.156162000
H	2.544117000	0.560253000	-2.137742000
H	3.062744000	0.690535000	2.123404000
H	1.368142000	6.248963000	-0.758570000
H	2.444521000	5.237618000	-1.758639000
H	0.707684000	4.823461000	-1.608179000
H	2.783787000	-1.466419000	-1.001920000
H	2.725648000	-1.509727000	2.066464000
H	0.548339000	-2.399672000	0.052639000
H	0.587663000	0.550377000	2.673216000
H	-1.056759000	0.076727000	4.440383000
H	-2.955411000	-1.061083000	2.853184000
H	-2.414960000	-3.256297000	-0.878426000
H	-1.141372000	-4.617521000	-2.516444000
H	0.737448000	-3.598673000	-3.788372000
H	1.307550000	-1.204998000	-3.432036000
H	0.055082000	0.137347000	-1.776812000
H	-3.173452000	0.584860000	-2.044979000

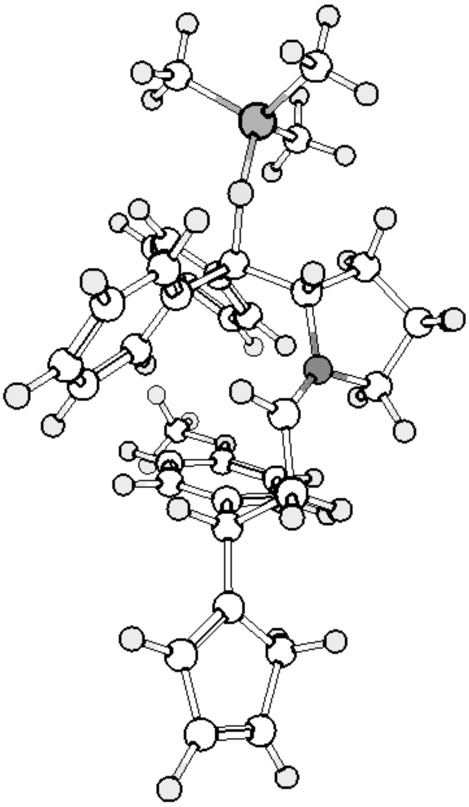

H	-3.219718000	2.992820000	-2.588155000
H	-1.924144000	4.607049000	-1.199194000
H	-0.515125000	3.757759000	0.664989000
H	-0.468828000	1.374894000	1.193525000
H	-5.962090000	1.356594000	1.307493000
H	-4.587387000	0.883868000	2.301840000
H	-4.328731000	1.799915000	0.803708000
H	-6.853309000	-0.375538000	-1.181947000
H	-5.497304000	0.529697000	-1.858379000
H	-5.554460000	-1.235241000	-2.018565000
H	-5.601919000	-2.940624000	0.726745000
H	-5.120650000	-2.152449000	2.236169000
H	-6.688975000	-1.786271000	1.509259000
H	-1.672905000	-2.289044000	1.308882000
H	-0.969096000	-1.661505000	4.125254000
H	1.220950000	-0.950576000	3.364841000
H	-2.265896000	0.482900000	2.366542000
C	4.627749000	-1.321027000	0.016233000
C	5.556138000	-0.792422000	1.085850000
C	6.898875000	-1.339930000	0.681168000
C	6.763060000	-2.061495000	-0.450142000
C	5.356427000	-2.049921000	-0.860062000
H	5.555578000	0.305828000	1.111714000
H	5.271957000	-1.120015000	2.097234000
H	7.812830000	-1.154683000	1.231046000
H	7.553602000	-2.570592000	-0.988521000
H	4.968050000	-2.547406000	-1.743191000
H	2.733804000	-2.918366000	0.993855000

Intermediate 5 (*S*) (=C_β-protonated intermediate 4)

C	-0.674779000	1.589822000	-1.215693000
C	-1.756346000	0.704828000	-1.320416000
C	-2.695086000	0.919344000	-2.341258000
C	-2.559358000	1.990088000	-3.223217000
C	-1.480897000	2.870321000	-3.103762000
C	-0.538986000	2.666295000	-2.097486000
C	-1.948176000	-0.532198000	-0.405821000
C	-1.297751000	-0.375429000	0.981620000
C	-0.721309000	-1.439907000	1.689292000
C	-0.280811000	-1.285168000	3.005477000
C	-0.412773000	-0.056248000	3.651166000
C	-0.983221000	1.015418000	2.962040000
C	-1.414008000	0.856457000	1.646673000
C	-1.512265000	-1.821289000	-1.221571000
N	-0.053047000	-2.205109000	-1.206067000
C	0.097992000	-3.694164000	-1.172112000
C	-1.314170000	-4.217644000	-1.434792000
C	-2.221654000	-3.132164000	-0.845266000
C	0.933545000	-1.378641000	-1.204863000

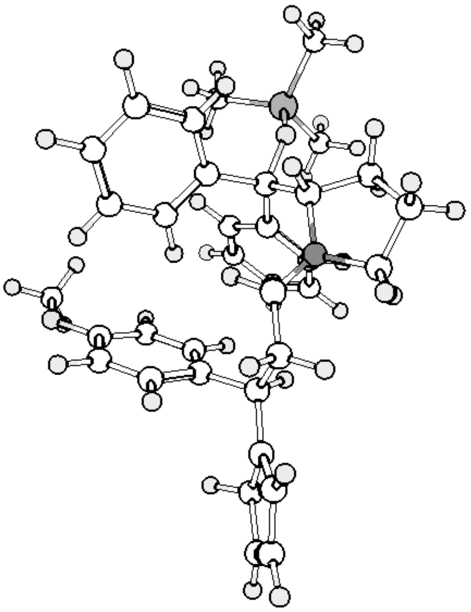

C	2.367577000	-1.739768000	-1.133459000
C	3.065158000	-1.062570000	0.091099000
C	2.828574000	0.444106000	0.106476000
C	3.278625000	1.251788000	-0.952107000
C	3.006023000	2.610139000	-0.978220000
C	2.283768000	3.205927000	0.070808000
C	1.871585000	2.423722000	1.155704000
C	2.140402000	1.050753000	1.156415000
O	2.020237000	4.529835000	-0.081402000
C	1.266788000	5.192204000	0.930719000
O	-3.336881000	-0.787208000	-0.272872000
Si	-4.589272000	-0.300261000	0.770228000
C	-4.742909000	1.574307000	0.833082000
C	-6.086520000	-1.055595000	-0.068790000
C	-4.332522000	-1.040080000	2.478423000
H	3.338390000	3.238521000	-1.797406000
H	1.320980000	2.853702000	1.982446000
H	1.781019000	0.449696000	1.986081000
H	3.858286000	0.810213000	-1.758547000
H	1.170067000	6.226949000	0.602450000
H	0.268148000	4.749188000	1.037153000
H	1.783043000	5.165271000	1.897908000
H	2.593108000	-1.473210000	0.994967000
H	2.836904000	-1.347575000	-2.046162000
H	0.684653000	-0.323227000	-1.248955000
H	0.465166000	-3.969637000	-0.178347000
H	-1.467185000	-5.199416000	-0.983045000
H	-3.228106000	-3.145064000	-1.258536000
H	-3.544205000	0.250403000	-2.428321000
H	-3.302114000	2.141375000	-4.000511000
H	-1.381421000	3.708835000	-3.785758000
H	0.296729000	3.347828000	-1.975110000
H	0.057165000	1.478300000	-0.425321000
H	-1.854205000	1.700635000	1.131795000
H	-1.099759000	1.978178000	3.450161000
H	-0.079403000	0.063568000	4.676971000
H	0.153504000	-2.132167000	3.527575000
H	-0.623696000	-2.418517000	1.239745000
H	-5.202075000	-0.829695000	3.111531000
H	-4.223975000	-2.128383000	2.420994000
H	-3.448217000	-0.637100000	2.978950000
H	-5.765069000	1.848791000	1.117049000
H	-4.068861000	2.029173000	1.563584000
H	-4.539075000	2.019095000	-0.146198000
H	-6.236651000	-0.633381000	-1.067705000
H	-5.977505000	-2.140009000	-0.173346000

H	-6.995252000	-0.867124000	0.512882000
H	-1.737781000	-1.602375000	-2.269217000
H	-1.481117000	-4.314296000	-2.512243000
H	0.833056000	-4.004163000	-1.915582000
H	-2.317228000	-3.227478000	0.238241000
C	4.529770000	-1.408199000	0.114115000
C	5.287856000	-2.033663000	-0.816040000
C	6.679402000	-2.104940000	-0.355417000
C	6.775431000	-1.522706000	0.856364000
C	5.419496000	-1.020961000	1.272637000
H	4.949484000	-2.433271000	-1.766271000
H	7.485359000	-2.558926000	-0.919728000
H	7.668120000	-1.415213000	1.459252000
H	5.412755000	0.065045000	1.439054000
H	5.082601000	-1.474824000	2.216105000
H	2.537932000	-2.816434000	-1.106042000

Intermediate 4 (*R*) N-protonated

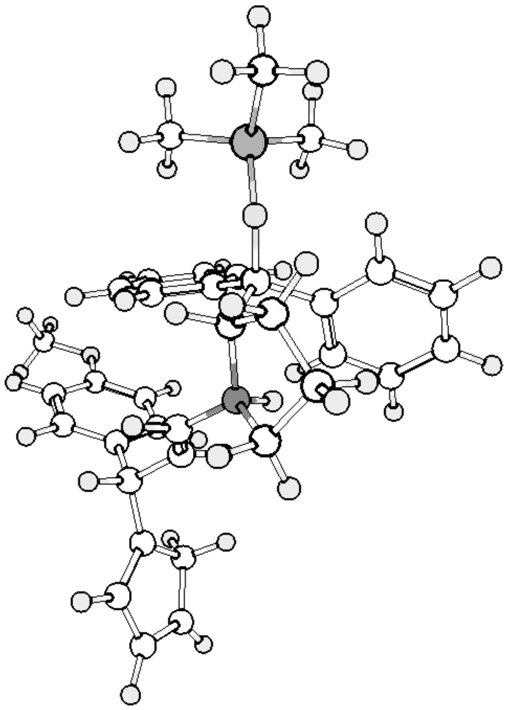

C	3.152448000	0.551908000	0.972038000
C	3.341835000	0.200708000	-0.374523000
C	3.838550000	1.174524000	-1.241659000
C	4.134618000	2.467346000	-0.802099000
C	3.922409000	2.803683000	0.539803000
C	3.439295000	1.829703000	1.427318000
C	3.024314000	-1.199581000	-0.898257000
C	1.615728000	-1.591944000	-0.511805000
C	0.625043000	-1.745368000	-1.382599000
N	-0.723929000	-2.138227000	-0.970534000
C	-1.871418000	-1.197122000	-1.407760000
C	-3.008569000	-2.155025000	-1.833806000
C	-2.623539000	-3.541587000	-1.295603000
C	-1.108639000	-3.540551000	-1.422995000
C	-2.260128000	-0.160950000	-0.279362000
O	-3.413791000	0.430318000	-0.843578000
Si	-4.196117000	1.934281000	-0.624728000
C	-3.161930000	3.347972000	-1.297687000
O	4.120937000	4.037980000	1.075028000
C	4.681190000	5.054993000	0.248280000
C	-2.606418000	-0.963904000	0.988404000
C	-1.597911000	-1.420202000	1.859369000
C	-1.896585000	-2.282265000	2.917918000
C	-3.211919000	-2.692813000	3.133737000
C	-4.224185000	-2.221586000	2.295107000
C	-3.924040000	-1.369040000	1.231334000

C	-1.176362000	0.906744000	-0.035676000
C	-0.357966000	1.381239000	-1.069714000
C	0.512016000	2.451394000	-0.868390000
C	0.569378000	3.087099000	0.371295000
C	-0.248869000	2.637901000	1.407480000
C	-1.107927000	1.558686000	1.206169000
C	-5.750003000	1.688953000	-1.645067000
C	-4.619222000	2.214693000	1.185902000
H	3.284485000	2.108028000	2.463927000
H	4.519130000	3.193503000	-1.507582000
H	4.005942000	0.927142000	-2.286840000
H	2.773165000	-0.177764000	1.681540000
H	4.783095000	5.934526000	0.884075000
H	5.668221000	4.764203000	-0.131080000
H	4.023769000	5.294147000	-0.597388000
H	3.068505000	-1.162677000	-1.992818000
H	1.427176000	-1.745345000	0.549235000
H	0.722120000	-1.614758000	-2.454527000
H	-0.565713000	-4.257525000	-0.805203000
H	-2.918140000	-3.648862000	-0.247037000
H	-3.065976000	-2.176981000	-2.925563000
H	-0.392667000	0.935599000	-2.056905000
H	1.152696000	2.782569000	-1.677951000
H	1.255309000	3.911234000	0.534329000
H	-0.217944000	3.124077000	2.377487000
H	-1.739261000	1.229873000	2.022926000
H	-4.714104000	-1.008637000	0.584502000
H	-5.254010000	-2.518006000	2.468702000
H	-3.449166000	-3.357419000	3.958055000
H	-1.104403000	-2.612748000	3.582627000
H	-0.584059000	-1.039416000	1.757288000
H	-6.367507000	2.593471000	-1.634622000
H	-5.505762000	1.463897000	-2.688161000
H	-6.359467000	0.866359000	-1.256639000
H	-5.324670000	3.049556000	1.267771000
H	-5.091524000	1.336728000	1.636842000
H	-3.739363000	2.471533000	1.781412000
H	-2.274754000	3.539987000	-0.688920000
H	-2.833783000	3.149413000	-2.322902000
H	-3.764613000	4.263441000	-1.314520000
H	-1.502258000	-0.641094000	-2.266295000
H	-3.074364000	-4.358937000	-1.861959000
H	-0.780550000	-3.639773000	-2.460859000
H	-3.970097000	-1.802714000	-1.467944000

C	3.994478000	-2.267782000	-0.444975000
C	4.429829000	-2.502688000	0.983785000
C	5.366518000	-3.675776000	0.874716000
C	5.466160000	-4.045229000	-0.418366000
C	4.615044000	-3.173884000	-1.233717000
H	4.934090000	-1.618198000	1.396254000
H	3.587094000	-2.711267000	1.660016000
H	5.877339000	-4.117078000	1.721129000
H	6.077455000	-4.848017000	-0.813592000
H	4.513798000	-3.246752000	-2.311964000
H	-0.762583000	-2.155547000	0.059908000

Intermediate 4 (*S*) N-protonated

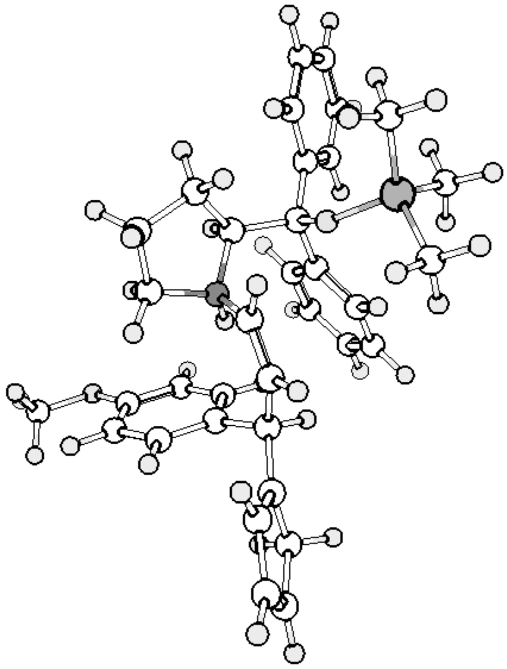

C	-2.519120000	-0.959273000	1.167072000
C	-2.980908000	-0.048145000	0.204414000
C	-3.877796000	-0.509992000	-0.763674000
C	-4.283686000	-1.845182000	-0.802926000
C	-3.783662000	-2.755514000	0.142463000
C	-2.897328000	-2.298523000	1.131342000
C	-2.471323000	1.395831000	0.186626000
C	-1.390808000	1.648631000	-0.849604000
C	-0.471069000	0.847453000	-1.383032000
N	-0.323459000	-0.591901000	-1.086734000
C	1.129616000	-1.118827000	-1.020007000
C	1.500866000	-1.230324000	-2.506324000
C	0.170371000	-1.501765000	-3.259856000
C	-0.924710000	-1.452165000	-2.187615000
C	2.040483000	-0.302961000	-0.044292000
O	2.202754000	0.968969000	-0.636739000
Si	3.342038000	2.228375000	-0.450450000
C	4.543948000	2.182550000	-1.892923000
O	-4.093029000	-4.073912000	0.191828000
C	-5.046268000	-4.589923000	-0.735825000
C	1.275059000	-0.218340000	1.295840000
C	0.857181000	1.010608000	1.814679000
C	0.199130000	1.082435000	3.045806000
C	-0.059953000	-0.077549000	3.776711000
C	0.314098000	-1.315831000	3.249424000
C	0.959634000	-1.384964000	2.014658000
C	3.417579000	-1.005047000	0.104542000
C	3.929092000	-1.441104000	1.334309000
C	5.207083000	-1.997201000	1.425833000
C	6.006867000	-2.124167000	0.292222000

C	5.522613000	-1.670619000	-0.935486000
C	4.248849000	-1.112959000	-1.023330000
C	2.281685000	3.777098000	-0.574905000
C	4.240574000	2.119091000	1.192919000
H	-4.987525000	-2.163276000	-1.562307000
H	-2.541816000	-3.003231000	1.875508000
H	-1.859665000	-0.616469000	1.956961000
H	-4.274460000	0.189548000	-1.493880000
H	-5.156432000	-5.646390000	-0.492173000
H	-6.016351000	-4.089562000	-0.632093000
H	-4.694093000	-4.491443000	-1.770418000
H	-1.991749000	1.577386000	1.161993000
H	-1.320379000	2.680337000	-1.185337000
H	0.262532000	1.205310000	-2.087881000
H	-1.868826000	-1.010256000	-2.496020000
H	-0.005166000	-0.741568000	-4.024267000
H	2.224818000	-2.031173000	-2.650133000
H	1.262039000	-2.356774000	1.635986000
H	0.107186000	-2.229066000	3.798563000
H	-0.555688000	-0.020087000	4.740359000
H	-0.099818000	2.050656000	3.435778000
H	1.043712000	1.914370000	1.251905000
H	3.924819000	-0.717806000	-1.975900000
H	6.139686000	-1.738672000	-1.826133000
H	6.999697000	-2.556401000	0.365147000
H	5.577049000	-2.320587000	2.393754000
H	3.352308000	-1.327530000	2.242039000
H	4.019026000	2.060983000	-2.846910000
H	5.101445000	3.124498000	-1.944453000
H	5.269052000	1.370745000	-1.794561000
H	2.912433000	4.672237000	-0.599092000
H	1.687545000	3.770176000	-1.495319000
H	1.591812000	3.885217000	0.268160000
H	3.547903000	2.038963000	2.035876000
H	4.913532000	1.258316000	1.225931000
H	4.842362000	3.023175000	1.339801000
H	1.008745000	-2.120793000	-0.600414000
H	0.166521000	-2.471629000	-3.760584000
H	-1.123601000	-2.427306000	-1.736710000
H	1.959449000	-0.298422000	-2.841958000
C	-3.612337000	2.373193000	0.045232000
C	-4.594895000	2.623283000	1.164027000
C	-5.576646000	3.590482000	0.560103000
C	-5.215432000	3.849516000	-0.712526000

C	-4.001010000	3.090347000	-1.034094000
H	-5.079021000	1.693065000	1.493677000
H	-4.100337000	3.039931000	2.053852000
H	-6.424941000	4.000174000	1.093475000
H	-5.722848000	4.510467000	-1.405319000
H	-3.509186000	3.112170000	-2.001061000
H	-0.810557000	-0.794521000	-0.203933000

References

[1] J. Jašík, J. Žabka, J. Roithová, D. Gerlich, *Int. J. Mass spectrom.* **2013**, *354*, 204-210.

[2] M. Frisch, G. Trucks, H. Schlegel, G. Scuseria, M. Robb, J. Cheeseman, G. Scalmani, V. Barone, G. Petersson, H. Nakatsuji, Gaussian, Inc. Wallingford, CT, **2016**.

[3] S. A. Ewing, M. T. Donor, J. W. Wilson, J. S. Prell, *J. Am. Soc. Mass. Spectrom.* **2017**, *28*, 587-596.

[4] L. Jasǐková, M. Anania, S. Hybelbauerová, J. Roithová, *Journal of the American Chemical Society* **2015**, *137*, 13647-13657.

[5] H. Gotoh, R. Masui, H. Ogino, M. Shoji, Y. Hayashi, *Angew. Chem. Int. Ed.* **2006**, *45*, 6853-6856.

[6] M. H. Haindl, M. B. Schmid, K. Zeitler, R. M. Gschwind, *RSC advances* **2012**, *2*, 5941-5943.

[7] B. S. Donslund, T. K. Johansen, P. H. Poulsen, K. S. Halskov, K. A. Jørgensen, *Angew. Chem. Int. Ed.* **2015**, *54*, 13860-13874.
